# Advanced Functional Materials for Intelligent Thermoregulation in Personal Protective Equipment

**DOI:** 10.3390/polym13213711

**Published:** 2021-10-27

**Authors:** Alireza Saidi, Chantal Gauvin, Safa Ladhari, Phuong Nguyen-Tri

**Affiliations:** 1 Department of Chemistry, Biochemistry and Physics, Université du Québec à Trois-Rivières (UQTR), 3351 Boulevard des Forges, Trois-Rivières, QC G8Z 4M3, Canada; 2 Laboratory of Advanced Materials for Energy and Environment, Université du Québec à Trois-Rivières (UQTR), 3351 Boulevard des Forges, Trois-Rivières, QC G8Z 4M3, Canada; safa.ladhari@uqtr.ca; 3 Institut de Recherche Robert-Sauvé en Santé et en Sécurité du Travail (IRSST), 505 Boulevard de Maisonneuve Ouest, Montréal, QC H3A 3C2, Canada; gauvin.chantal@irsst.qc.ca

**Keywords:** thermoregulation, personal protective equipment, smart textiles, performance, productivity

## Abstract

The exposure to extreme temperatures in workplaces involves physical hazards for workers. A poorly acclimated worker may have lower performance and vigilance and therefore may be more exposed to accidents and injuries. Due to the incompatibility of the existing standards implemented in some workplaces and the lack of thermoregulation in many types of protective equipment that are commonly fabricated using various types of polymeric materials, thermal stress remains one of the most frequent physical hazards in many work sectors. However, many of these problems can be overcome with the use of smart textile technologies that enable intelligent thermoregulation in personal protective equipment. Being based on conductive and functional polymeric materials, smart textiles can detect many external stimuli and react to them. Interconnected sensors and actuators that interact and react to existing risks can provide the wearer with increased safety, protection, and comfort. Thus, the skills of smart protective equipment can contribute to the reduction of errors and the number and severity of accidents in the workplace and thus promote improved performance, efficiency, and productivity. This review provides an overview and opinions of authors on the current state of knowledge on these types of technologies by reviewing and discussing the state of the art of commercially available systems and the advances made in previous research works.

## 1. Introduction

### 1.1. Thermal Stress in the Workplace

Thermal stress is among the most common physical hazards in various work sectors. In fact, any worker exposed to a high heat load through a combination of his or her metabolic heat during work, environmental factors (air temperature, humidity, air movement, heat transfer by radiation), and the clothing requirements of his or her job can suffer health problems [1]. In addition, exposure to extreme temperatures in workplaces involves physical hazards for workers. Workers in firefighting, construction, mining, smelting and primary metal processing, metal product manufacturing, forestry, agricultural, food manufacturing, and police services are among the most exposed sectors to heat-related hazards. Workers in construction, agriculture, fishing, logging, forestry, and other outdoor activities are at risk of cold stress [2].

Indeed, the exposure to extreme temperatures can lead the worker to a state of heat stress, which occurs when the body is unable to maintain its temperature between 36 and 37 °C [3]. Heat syncope, heat exhaustion, heat stroke, dehydration, heat cramps, miliary eruptions, hyponatremia, and rhabdomyolysis are among the diseases or health disorders due to heat exposure. Hypothermia, immersion feet, and frostbite are the most significant injuries and illnesses caused by exposure to extreme cold [2]. Therefore, the prevention of thermal stress risks should be a priority in order to avoid any negative effects on workers’ health and safety [4]. Adequate prevention of heat stress risks not only provides a sense of comfort for the worker toward his work environment, but it can also have a positive impact on the productivity rate and result in a decrease in the employer’s number of injuries [5].

In addition to being a direct cause of serious injuries in the workplace, thermal stress can indirectly lead to accidents and other types of injuries. A poorly acclimated worker may have reduced performance and alertness and therefore may be at greater risk of accidents and injuries [6,7,8]. One of the main risks indirectly related to working in extreme cold is the decreased manual function, which can quickly impair task performance and increase the risk of accidents or intensify a hazardous situation [9]. Research has shown that manual dexterity is impaired during work in cold storage warehouses [10]. Cold can also reduce alertness and impair cognitive performance, increasing the risk of inappropriate mental actions leading to accidents. Indeed, one study was able to demonstrate that reaction time and signal detection decreased in workers exposed to a temperature of -20 °C for more than 45 min [11].

Exposure to extreme temperatures can also temporarily reduce work capacity and affect productivity [2]. As a result, thermal stress can directly alter operational capacity, both by decreasing work tolerance and by requiring changes in work schedules, such as longer rest and recovery breaks [2]. For some professions, such as firefighters, the interaction between high physical exertion and heat is the main cause of death [12]. According to studies conducted in the United States, thermal and physiological stress during interventions is associated with an increased risk of cardiovascular accidents, which are the most common cause of death among firefighters [13]. In addition to the impact of heat on cardiovascular behavior, the thermoregulatory mechanisms of the human body under thermal stress and the physiological changes they imply can alter the functions of several organs related to the absorption and chemical metabolism. Heat exposure has been shown to be associated with increased pulmonary and dermal absorption of xenobiotics [14].

**Figure 1:**
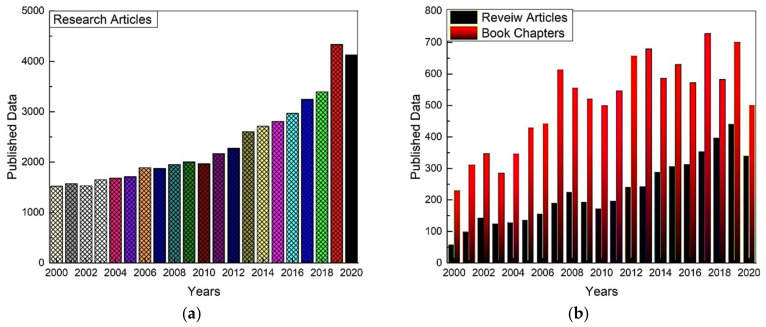
Published data for personal thermal management from 2000 to 20 June 2020. (**a**) Research articles published during the last two decades. (**b**) Review articles and book chapters published during the last two decades. Reproduced with permission [3]. Copyright 2020, Elsevier.

The protection of workers against thermal risks becomes even more important since, according to experts, the current climate change context will contribute to emphasizing the impact of thermal constraints in the workplace [15,16,17]. Over the past few decades, many research studies related to thermal management have been witnessed, as shown in Figure 1.

As a result of the importance of preventing the risks of thermal stress, recommendations and measures have been planned by the authorities. These regulations recommend redesigning the workstation, reducing the workload, and wearing appropriate personal protective equipment (PPE) to ensure that thermal stress thresholds are not exceeded. However, some studies have shown that despite compliance with these regulations, some workers may be subject to thermophysiological constraints depending on their age, sex, physical fitness, or state of health [12]. Moreover, these types of measures against thermal stress are sometimes far from being applicable in certain environments such as agriculture [18,19]. Regulations are sometimes very cautious and sometimes overestimate the level of thermal stress, while for heavy work in indoor workplaces, they may underestimate exposure [20]. Prevention measures remain unclear and sometimes unrealistic in the face of reality [21].

### 1.2. Personal Protective Equipment Design Challenges

In addition to several gaps in the established regulations to counter the risks of thermal stress in the workplace, PPE can accentuate the impact of thermal stress, as many of these items of equipment lack comfort [22]. PPE is designed primarily to protect workers against external hazards such as chemical, biological, thermal, and mechanical. Various polymeric materials are commonly used for the fabrication of PPE [23]. For instance, protective gloves can be made with polymers (nitrile, latex, neoprene, poly(vinylacetate), polyvinyl chloride (PVC)), with woven or knitted textiles materials (aramid fibers (Kevlar^®^), high-performance polyethylene (HPPE)), coated or not with polymers, in single or multiple layers [24,25,26,27]. Depending on the protection required, different synthetic materials can be used also in the fabrication of protective clothing, such as meta-aramide (Nomex^®^), para-aramide (Kevlar^®^), polybenzimidazole (PBI), melamine (Basofil^®^), polyphenylene benzobisoxazole (Zylon^®^), and polyimide for heat and flame hazard, polyurethane (PU), chlorinated polyethylene (CPE), polytetrafluoroethylene (PTFE), PVC, and polyvinylidene chloride (PVDC) as impermeable layers and moisture barriers [26,28], or activated carbon impregnated foam, fluoro-polymer coatings, polyurethane nonporous membrane, or elastomers for chemical protection [23,29,30,31]. Being a multidisciplinary field calling for several technological knowledge, the materials used in the design of protective equipment has been the subject of several technical reviews. While some of these studies have been devoted to a global state of the art of materials used and the evolution of associated needs [23,26,32,33], others analyzed specific developments and needs to counter a particular type of risk, for example, reviews specifically dedicated to advances and applications of materials for chemical protective clothing [25,30]. Some contemporary research studies have even evoked a potential application of nanofiber materials in protective clothing. These materials obtained from nanoparticles mixed with polymer solutions can offer greater breathability, a selective filtration potential along with an improved liquid chemical and aerosol particle retention capability compared with current commercially available membranes [25,34,35].

However, the materials used in the design of several types of PPE tend to avoid the adequate dissipation of body heat [36]. Thus, workers such as firefighters or metal fabricators may be exposed to more thermal and physiological stresses due to their type of protective equipment [37]. As reported by occupational health and safety experts, workers often find protective equipment uncomfortable, too hot, or too bulky, which does not encourage them to wear it regularly, thus accentuating potential risk situations [38].

Given the existing shortcomings in the prevention of thermal stress in workplaces due to conventional conception in the design of protective equipment and the inefficiency of the established standards and recommendations, it is essential to develop new tools and equipment to ensure thermal risk management adapted to the individual situation of the worker and his or her work environment. In such a context, smart textile technologies integrated into personal protective equipment have a very great potential to respond to many issues related to thermal risks. Thus, using them in the development of PPE presents great potential for the field of occupational health and safety [22,39,40,41].

Being based on textronic (e-textiles), conductive textiles, functional textiles, and flexible and extensible electronics, smart textiles can contribute to the development of thermal regulation systems [42,43] to better protect workers against the risks of thermal stress while offering them greater comfort. They can also be used in the development of tools for measuring external and internal garment temperatures, as well as body temperature [39,40]. In addition to being the basic textile material, polymeric materials are also widely used in the production of smart textiles whether in the design of sensors or actuators, their methods of integration into textiles, conductive yarns fabrication, conductive polymers coating, functional coating, or embedding conductive fillers [44,45,46].

Recent technical reviews often report on knowledge in the area of smart textiles [44,45,47], including a number of studies that mention their potential use in PPE design [40,48,49]. Although some other studies have made reviews of the heat stress state in conventional PPE [50,51], to our knowledge, no reviews are specifically related to the analysis of smart textile technologies for the prevention of thermal stress risks while wearing PPE. In fact, despite the studies that have separately reviewed heating, cooling, or thermal sensor technologies integrated into clothing [44,45,48], no study exists on a complete analysis of all the technologies that facilitate intelligent thermal management in PPE. Furthermore, the continuous evolution of smart textile technologies in an increasingly connected world, both at the societal and industrial levels, requires an update of knowledge to better support the adaptation of such technologies to occupational health and safety applications.

In spite of the recent technological progress, a preliminary analysis has shown that most of the current commercial solutions are dedicated to the fields of sport and leisure, and very few are related to occupational protective equipment [52,53]. Indeed, heating systems integrated into different types of clothing and accessories have emerged in recent years [54]. However, these systems suffer from a lack of comfort and are difficult to use in a work context. 

While some integrated systems have presented risks of overheating [55], others suffer from a lack of temperature control [56]. Integrated cooling systems are usually based on passive devices composed of multilayer structures or functional coatings, which limits their reactivity to temperature variations [57]. Moreover, active integrated cooling systems remain cumbersome and energy consuming [43] and sometimes not very efficient in extreme climatic conditions [58]. The development of self-regulating temperature systems using functional materials of phase change materials types [59,60] has attracted the attention of many research groups [59]. However, these materials in their current state remain limited by their overall enthalpy of phase change or thermal window. They are active during their phase change period but cease to function when the phase change is completed [61]. Despite the emergence of commercial products incorporating smart textile technologies, garments with integrated sensors capable of detecting thermal stressors in order to mitigate the risk of contact and prolonged exposure to extreme temperatures in workplaces are also rare. Although isolated cases have been developed for some trades in a few countries [62], most work remains limited to research [63].

Using the potential of advanced materials both in the design of conductive textiles and in the development of thermal sensors and actuators to be integrated in protective equipment can provide a reliable solution to fill current lacks in the design of intelligent thermal management tools in the context of occupational health and safety. Therefore, the present study aims to present a review of current knowledge of these technologies facilitating smart thermoregulation in personal protective equipment.

## 2. Temperature Sensor

This part of the study focuses on systems that provide data on the body temperature of an active person. It also discusses the sensors that can be integrated into PPE in order to facilitate the acquisition of the temperature of the microclimate under the clothing or the outside temperature with the objective of warning the worker in case of prolonged exposure to extreme temperatures.

Real-time monitoring of body temperature is very important in order to prevent in time the occurrence of disorder in many organs during exposure to high thermal stress [64]. The calculation of body temperature is commonly based on the measurements of the core body temperature (T_c_) and the skin temperature (T_s_). While T_c_ is adjusted by thermoregulatory mechanisms of the body, T_s_ is affected by blood circulation and is related to heart rate (HR) and metabolic rate [64]. Therefore, temperature sensors used for body temperatures (T_s_ and T_c_) must operate efficiently over a temperature range of 35 to 40 °C and ideally offer a measurement accuracy of 0.1 °C [65].

### 2.1. Methods to Measure Body Temperature

**Figure 2:**
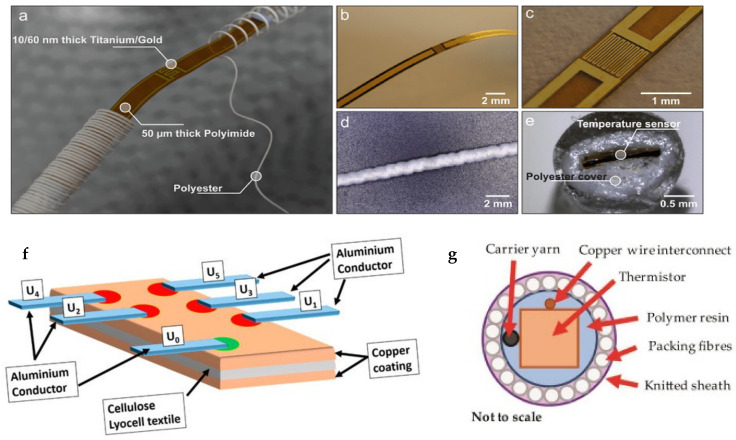
Temperature sensors: (**a**) Concept of the flexible temperature sensor embedded within the fibers of a textile yarn; (**b**) Bending of the uncovered flexible resistance temperature detectors RTD; (**c**) RTD Close-up sensing area. (**d**) Resistance temperature detectors embedded within a braided polyester yarn; (**e**) Cross-section of the braided temperature-sensing yarn ((**a**–**e**) [7]); (**f**) Lightweight and flexible conductor materials in a thermocouple array with copper-coated cellulose textiles [8]; (**g**) A cross-sectional schematic of encapsulation for a thermistor within a yarn. The standard encapsulation is composed of three layers: a polymer resin, packing fibers, and a knitted sheath [9].

Various types of analog electrical sensors have been deployed in recent years to measure body temperature (T_s_ and T_c_). These sensors are generally based on thermistors, resistance temperature detectors (RTDs) [66] (Figure 2a–e), or thermocouples [64] (Figure 2f,g).

Rectal thermometry is the most accurate method for measuring body temperature, and its value is recognized as the most representative of core body temperature [64]. It has been widely used as the standard measurement in many heat stress studies, including work on the development of heat stress indices [67,68,69,70]. However, rectal thermometry is an intrusive method that requires private arrangements and is therefore unsuitable for the continuous monitoring of workers with high physical activity [65]. Although heart rate can be used for indirect inference of T_c_ [71,72], some other studies have also proposed an estimation of T_c_ from T_s_ [73,74].

Thus, in order to contribute to the protection of individuals against thermal aggressors, the scientific community has been interested in the development of temperature sensors that can be integrated into personal protective equipment [75]. These sensors could measure T_s_ and monitor the microclimate temperature between the body and the clothing or the outside temperature during exposure to thermal aggressors. While much work has been dedicated to the development of temperature sensors based on smart textile technologies and flexible electronics, a very limited number of studies have been devoted to the systems integrated into clothing.

In fact, the main motivation for the development of textile or flexible sensors has been to overcome the obstacles that hinder portable temperature detection despite the progress made [76]. Most thermistors or thermocouples used in wearable technologies [77] are sensitive to deformation, which can impair temperature sensing with bending or twisting of the sensor [76]. To counter the strain dependence of this type of sensor, some researchers have proposed a hybrid approach based on the integration of a small rigid thermistor embedded in a flexible and extensible matrix [78]. In one of these selected works, an NTC-type thermistor (having a negative temperature coefficient) in association with conductive textile threads was integrated in a bamboo belt to monitor the body temperature of newborns. Despite an encouraging detection accuracy of 0.1 °C of the prototype tested in a hospital setting, the concept lacked mechanical strength due to the use of knots to ensure the connection between the sensor and the signal-transmitting conductive textile threads [79]. In more recent work, the aspect of mechanical strength could be improved by encapsulating a standard thermistor in a polymer resin microcapsule, then embedding it in the fibers of a yarn, and then incorporating it into a textile structure [78,80,81,82,83]. As part of this work, ongoing optimizations have been made, including encapsulating the commercial thermistor in a microcapsule of thermally conductive resin to improve the sensitivity of the sensor [82] or connecting the sensor leads to a microcontroller and a Bluetooth module for wireless transmission of the collected data [78,80]. However, the proposed concepts still require further optimization, particularly in terms of detection accuracy, as differences of 0.5 to 1 °C were observed between the reading and the actual temperature of the sample surfaces [80,82].

Temperature sensors can also be manufactured from textile materials composed of conductive fibers or yarns using conventional textile manufacturing technologies such as weaving, knitting, or embroidery [65]. Depending on their operating principles, these types of sensors can be classified as thermocouples or RTD-type detectors [84].

**Textile thermocouples**: They exploit the Seebeck effect, which is based on the development of a corresponding potential difference between the junctions of two different metal structures due to the temperature difference between the junctions [65]. Structures with textile electrode pairs consisting of graphite fiber/antistatic fibers, non-woven graphite/silver-coated yarns, or hybrid knitted steel/alloy constantan wire composition have been used to design textile thermocouples [85,86]. However, these thermocouples exhibit a non-linear relationship between potential change and temperature and are characterized by low accuracy and sensitivity compared to conventional wire thermocouples [65]. In addition, they are also sensitive to changes in environmental relative humidity [86].

**Textile RTDs**: They use the temperature dependence of materials with electrical resistivity to determine temperature. These sensors can be developed by incorporating wires or conductors with a high temperature resistance coefficient into the fabric [65].

Therefore, fibrous sensors of RTD types could be developed by inserting metal wires (copper, nickel, and tungsten) in a knitted structure [87], by integrating metallic filaments in the middle of a double-knitted structure with different densities of metallic wire incorporation [88], by using cotton yarns coated with a PEDOT-PSS conductive polymer solution and a polystyrene encapsulation layer embeddable in a textile structure by weaving or stitching [89], by embroidering chromium–nickel austenitic stainless steel threads on a textile substrate [90], or by embroidering a hybrid thread composed of polyester fibers and a stainless steel micro thread on a fabric [91], which could be inserted in the outer layer of firefighters’ clothing [92]. This last work was able to demonstrate that textile RTDs offer increased accuracy and sensitivity, shorter response time, and better linearity with temperature compared to thermocouples [65]. However, these sensors could not provide localized temperature measurements, as the measurement is instead performed over the entire area of the textile [78,92].

**Figure 3:**
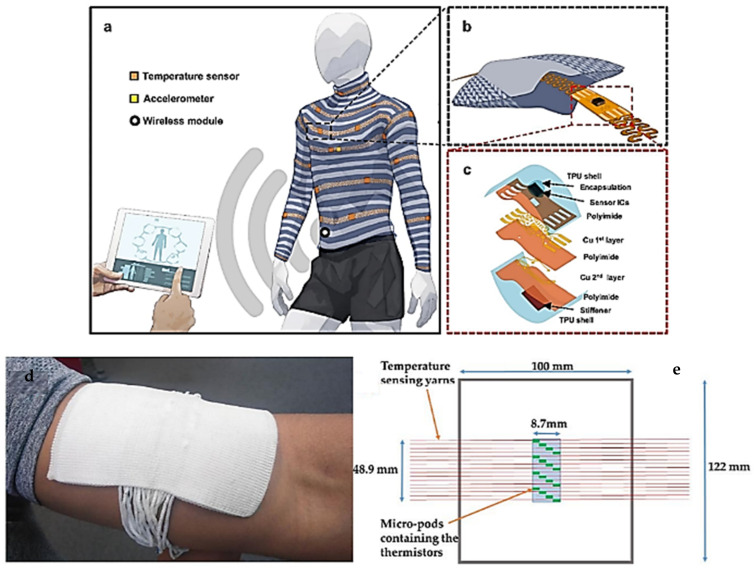
Thermal detection of smart textiles. (**a**) Illustration of spatiotemporal sensor mapping of the body with temperature and accelerometer (heart beat and respiration); (**b**) Wearable textile with embedding stretchable–flexible electronic strips; (**c**) Exploded view of a sensor island. Reproduced with permission [98]. Copyright © 2021, Wicaksono et al. (**d**) Health monitoring textile with temperature-sensing yarns; (**e**) A schematic of the textile thermograph (**d**,**e**) [78].

Some studies, on the other hand, have reported an optical sensing approach for measuring body temperature by integrating optical fibers into the textile structure [93]. As a result, a distributed Bragg reflector with the ability to reflect light of specific wavelengths and transmit it to other wavelengths has been used [94]. The Bragg reflector was encapsulated with a polymeric substance and then woven into the fabric structure [95]. The authors have also analyzed mathematically the transmission of heat from the skin to the environment via the Bragg reflector and used a weighted coefficient model to estimate body temperature considering the wavelength shift as a function of temperature. They have also reported a high accuracy of ±0.18 °C in a range of 33 to 42 °C [95]. A new method of integrating optical fibers constituting a Bragg reflector into a hollow double-walled fabric structure has also been proposed in a recent study [96]. Despite the high accuracy provided by Bragg reflectors, the concept is far from being applicable to the design of a wearable device, as it requires connection to at least one amplified broad-spectrum light source and an optical spectrum analyzer [96]. The design of a textile heat flux sensor has also been proposed by investigating a method of inserting a constantan yarn into three different textile structures (polyamide-based knitted fabric, non-woven aramid, and aramid-based woven fabric), which is followed by several treatment and post-treatment steps including the electrochemical deposition of copper on the constantan yarn to obtain a thermoelectric yarn [97]. Figure 3 shows some examples of integrated flexible sensors in textiles and yarns.

### 2.2. Flexible Temperature Sensors

Although these studies are still at a very preliminary stage, some research groups have attempted to develop shape memory textile sensors. The concept is based on the use of shape memory polymers sensitive to external stimuli such as light or temperature. Recently, the innovation of sol gels, conductive polymers, and copolymers as biomaterials enabled the miniaturization of biological analyses in an integrated chip with new generation sensors using a Si light source with a wide visible wavelength range as an optical biosensor [99].

Temperature sensing functionality can be obtained by spinning shape memory polymer fibers, such as polyurethane fibers, with other types of fibers to make textile fabrics, or by coating shape memory polymer emulsions on a woven or knitted fabric [100]. Other configurations of shape memory materials applicable to fabrics include silicon [101], nanofibers, and shape memory foams. In order to facilitate the characterization of the thermal sensitivity of textile shape memory sensors, a shape memory coefficient based on the change of deformation angle with temperature variation was suggested [102].

**Figure 4:**
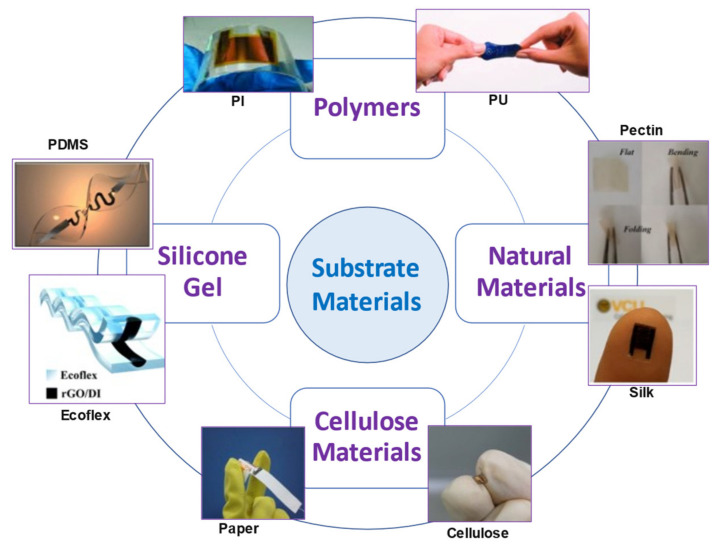
Schematic illustration of flexible sensors materials. Clockwise from the right top: polyimide (PI) [103], polyurethane (PU) [104], pectin [105], silk [106], cellulose [107], paper [108], ecoflex [109], polydimethylsiloxane (PDMS) [110].

Many researchers have also worked on the development of flexible temperature sensors with the deposition of materials that facilitate temperature detection on flexible polymeric substrates using printing, coating, and lamination techniques [65] (Figure 4).

If they maintain their mechanical strength, these types of sensors can then be attached to fabrics or integrated into textile structures [100]. In this context, several studies investigated the development of flexible temperature sensors based on graphene as a highly conductive material from an electrical and thermal point of view [111,112]. Therefore, electrical resistance temperature-sensing layers have been developed by printing a graphene oxide formulation on polyimide and polyethylene terephthalate substrates, which is followed by infrared firing to obtain a material with a negative temperature coefficient [113]. A layer with an RTD property having a positive temperature coefficient (PTC) was also developed by deploying the plasma-assisted chemical vapor deposition method of graphene nanosheets on a polydimethylsiloxane (PDMS) substrate [114]. In addition, a stretchable thermistor was designed by integrating a graphene-based dispersion in a PDMS-based matrix as a detection channel, which was associated with electrodes formed from silver nanofilaments in polycarbonate membranes [111]. Thanks to the use of graphene, temperature sensitivities very close to those of metal oxide materials used in classical sensors have been obtained in a flexible structure [113]. However, the stretchable structure based on graphene has shown strong variations in its thermal behavior as a function of mechanical deformation [114], which may constitute a limitation for their integration in textile structures.

Printing techniques were also used to design flexible temperature sensors [115]. The most notable works include the screen printing of a carbon-based ink on a polyimide sheet to obtain a PTC thermistor-type structure [43], the screen printing of various resistive inks on polyethylene naphthalene being protected by a passivation layer of dielectric ink and plasma post-treatment to improve the temperature resistance coefficient of the printed layer [116], the ink-jet printing of a dispersion based on nanoparticles of nickel oxide in the space between two silver-printed electrodes using a polyimide substrate to develop an NTC thermistor [117], a 100 × 100 pixel array all-CMOS (Complementary metal–oxide–semiconductor) monolithic microdisplay system has proven possible to create a high-optical power efficiency all-CMOS microdisplay [118], and the ink-jet printing of a silver complex dispersion on a polyimide substrate to obtain a layer with PTC thermistor behavior [119]. Overall, the printed thermosensitive structures were able to offer high temperature sensitivity, while having very low hysteresis during heating and cooling cycles [116,117,119]. Screen printing of PEDOT-PSS conductive polymer and carbon nanotubes dispersion on polyimide substrates and the use of silver-based printed electrodes has also allowed the development of RTD layers. Then, the printed RTD layers were combined with radio signal transmittances to design a label [120] or bandage [121] to be placed on an individual’s skin to communicate with an external reader device [120]. Printed temperature sensors have also been developed on paper substrates [122,123]. In their current state, these types of development are rather intended for the packaging field and require work to reformulate the inks used to make them compatible with non-porous polymeric substrates with surface properties different from those of paper [64].

The formation of composite layers on flexible substrates has also been another method for the design of flexible temperature sensors. In this register, a composite film with RTD properties could be obtained by coating a mixture of poly o-methylaniline and manganese oxide (Mn_3_O_4_) on a solid substrate [124]. In addition, a composite film based on tellurium nanofilaments in a poly-3-hexylthiophene matrix deposited on a flexible substrate was used to obtain RTD behavior [125]. The deposition of graphite particles dispersed in a PDMS matrix on inter-digitalized copper electrodes prefabricated on a polyimide substrate was also deployed to obtain a composite film demonstrating RTD properties [126]. The dispersion of multiwall carbon nanotubes in a toluene solution of polystyrene–ethylene–butylene–styrene (SEBS) deposited on gold electrodes fabricated on a polyimide substrate resulted in a composite film showing NTC-type thermoelectric characteristic of a sensitivity comparable to the highest values for metals [127]. In a similar study, a mixture of multiwall carbon nanotubes and a polyvinyl benzyl derivative with trimethylamine coated on a pair of gold electrodes fabricated on a polyimide film led to the formation of a composite film with RTD behavior and a sensitivity comparable to that of metals [128]. The combination of a binary composite film of polyethylene and polyethylene oxide loaded with nickel microparticles with a passive RFID antenna has led to the design of a portable RTD temperature sensor. Despite the portability of this prototype sensor, it had three times the sensitivity of similar commercial sensors and a significant measurement error of ±2.7 °C [85]. In this framework, an array of 16 RTD-type temperature sensors was also fabricated with narrow serpentine gold traces using a microlithography technique on thin layers of polyimide to design an electronic skin to be fixed to the skin by the action of Van der Waals forces [129].

### 2.3. Radio-Frequency Identification (RFID)

As part of the development of flexible temperature sensors, other work has opted for radio-frequency identification (RFID) tags to be placed on the skin to measure T_s_. For example, these studies have contributed to the development of a passive ultra-high frequency (UHF) RFID tag, which is based on the temperature dependence of the ring oscillator frequency and allows data to be sent to a reader at 868 MHz with a range of 2 m [130]. Similar work has developed a flexible RFID tag comprising a commercial microchip providing direct thermal reading and an antenna designed with copper adhesive transferred onto a polycaprolactone membrane to be attached to the individual’s arm or abdomen with hypoallergenic cosmetic glue. The label allowed the data collected to be sent in a band of 780–950 MHz and a range of 30–80 cm to a nearby reading device [131]. According to the analyses of this study, the label placed on the skin requires that the label itself does not alter the locally measured T_s_ and must allow the natural perspiration of the skin to be preserved [131]. In a similar work, a modular patch with two detachable components, including a reusable inner part housing electronic element (the antenna, the integrated circuit, and the battery) and a disposable cover encapsulating the sensor associated with a medical-grade adhesive ensuring adhesion to the skin surface, made it possible to develop a real-time epidermal temperature sensor using UHF-type RFID communication [132]. In addition to a deviation of 0.6 °C from reference measurement methods, the influence of human variability and environmental conditions on the sensitivity of this sensor remains to be clarified [132].

Advanced materials have also been applied to the optimization of certain types of portable devices such as portable in-ear devices, which is a new technological trend in recent years to measure body temperature and other physiological parameters through sensors that hold. A dispersion based on graphene, as a highly conductive material known for its strong optical absorption in the infrared range, has been coated on the silicon substrate of the lens of IR thermopiles used in portable in-ear devices with the aim of increasing the accuracy of measurements in such a thermopile [133].

### 2.4. Textile Prototypes with Flexible Temperature Sensors

The overall analysis of the research on temperature sensors integrated in textile structures, textile sensors, and flexible temperature sensors has shown that the vast majority of these studies remain at the level of proof-of-concept of components that are still to be integrated in clothing, although some work is dedicated to temperature sensors integrated in work clothing. In one of these studies, the ambient temperature and heat flux through the garment could be measured by a modified PTC grade sensor network integrated in the outer and inner side of the firefighters’ protective clothing with the transmission of the collected data to an external reader device using the Zigbee communication protocol. The prototype, tested on a thermal manikin in the laboratory, had yet to be validated in an operational environment [134]. A work jacket for oil workers operating in extreme cold was also developed using an embedded IR temperature sensor and two combined humidity/temperature sensors. The jacket consisted of one humidity/temperature sensor on the outside of the jacket, a second pair of sensors placed on the opposite side of the jacket on the inner side, and the IR sensor, which was integrated on the inside of the sleeve for non-contact measurements of T_s_ at the wrist [135]. This jacket equipped with temperature sensors could be optimized by, among other things, placing a layer of heat-reflecting film in the lining of the jacket on the inside to reduce the influence of the person’s heat on the outside temperature measurements and adding a layer of elastomeric material around the outside sensor to reduce the heat flow through the jacket in the vicinity of the sensor [136].

A smart glove and an armband each comprising two electrodes made of conductive textiles to measure the galvanic skin response and a sensor from a commercial digital thermometer detecting T_s_ were developed to assess the conditions of soldiers in real time. Both were tested on about 40 subjects, but the assembly remained cumbersome, and the main signal transmission lines were fabricated with electrical wires that could be damaged during use or maintenance [137]. A thermistor microencapsulated in a wire [78,82] has been integrated into a cuff, glove, and sock for measuring T_s_ [138]. The cuff contained four wires each with a thermistor, while the glove and sock were based on a set of five wires each containing a thermistor. The contact pressure on the hands was found to influence the measurements due to the deformation of the sensor wire structure in the glove. In addition, the fit of the sock can also affect the measurements, as can the wearing of a shoe or walking, which appear to strongly influence the temperature measurements. These measurement errors seem to show that monitoring the foot skin temperature by sensors integrated in the textiles could be challenging for applications where accurate measurements are required. According to this study, fabrics containing sensor yarns should be manufactured according to the contact pressure exerted at the temperature measurement emplacement [138].

### 2.5. Commercial Textile with Temperature Sensors

Due to the need to monitor patient health or athlete performance, more and more portable products with temperature sensors have appeared on the market in recent years. Some integrate temperature sensors into their structure and others are based on the deployment of advanced materials. Among the commercial devices for biometric sign detection in the form of portable accessories in recent years, Biofusion (by Biopeak, Ottawa, Canada) and QardioCore (by Qardio, San Francisco, US) offer integrated systems that use contact RTD-type temperature sensors to measure T_s_ from the chest.

Based on printable electronics techniques, flexible temperature sensors have also been produced and have entered the market to serve areas such as transportation, logistics, food supply chain, and home appliances. Thanks to their flexibility, their integration into textile structures seems conceivable. However, their adaptation to textile structures still requires a certain number of technical challenges to be taken up, especially in terms of durability in wear or maintenance, especially in washing [139]. These types of flexible sensors such as those proposed by the company PST sensors (Cape Town, South Africa) are mainly printed thermistors associated with an electronic chip. The conductive ink used in these types of development is based on a composition that, once printed, demonstrates RTD properties [113,120].

Then, circuits containing these types of printed thermistors can be combined in a hybrid system with wireless data transmission protocols [140]. According to the manufacturers of these types of flexible thermistors, the sensors developed provide measurement accuracy ranging from ±0.1 to ±0.25 °C. While providing a very low response time of 100 to 250 ms, these flexible temperature sensors have the advantage of operating with low working powers in the nano or micro watt range. Graphene conductive layers with RTD characteristics, demonstrating a very high sensitivity to temperature changes [111], have recently been successfully used in the design of a connected insole based on an integrated thermistor to continuously monitor temperature changes in patients’ feet and detect early signals of foot ulcers in diabetics (Smart Insole by Flextrapower, New York City, NY, USA). These types of products for the medical field may be of interest for knowledge transfer toward an occupational health and safety application.

Regarding products marketed in the form of temperature sensors integrated into clothing, a very limited number of products exist on the market. These products were mainly developed to help protect firefighters [39]. In this context, the companies Ohmatex (Aarhus, Denmark) and Viking (Esbjerg, Denmark) jointly presented a firefighter suit containing thermal sensors integrated inside and outside the firefighter’s clothing to monitor environmental and near-body heat, respectively. The sensors are connected to LED displays on the sleeve and shoulder of the jacket. Above a certain temperature threshold detected on the outside or inside the jacket, the flashing of the display alerts the user. Despite the presence of an integrated electronic device, this garment had the advantage of withstanding at least 25 wash cycles.

The Balsan fire jacket (by TeckniSolar Seni, Saint-Malo, France) was also equipped with temperature and humidity sensors. A temperature sensor on the outside of the jacket measures the environmental temperature and a pair of temperature/humidity sensors on the inside of the jacket measures microclimatic conditions close to the body. When parameters exceed a certain level, an audible and visual alarm alerts the firefighter [62,141].

### 2.6. Apparels Measuring Thermal Stress

The review of research literature for measuring body temperature tools and sensors that can be integrated into protective equipment to assess the microclimate under the clothing or the environmental temperature in order to develop warnings in case of very high thermal stress are presented in Table 1.

### 2.7. Temperature Sensors Challenges

Concerning the studies dedicated to temperature sensors that can be integrated in textiles, the present state of the art has found that a lot of work is dedicated to the design of temperature sensors based on smart textiles and flexible electronics [53,93,157], and a very limited number of studies on sensors integrated in clothing has been identified. A hybrid approach has been proposed to integrate rigid thermistors in a flexible matrix in the textile structure. Despite several works related to integrated thermistors, some prototypes lack mechanical strength, while others require optimizations regarding detection accuracy. Another method has been to design fibrous sensors such as RTDs or thermocouples. According to the studies analyzed, fibrous thermocouples require significant optimization effort, because in addition to low sensitivity and low measurement accuracy, they have proven to be sensitive to environmental humidity. Although the textile RTDs developed in analyzed studies have provided better accuracy, higher sensitivity, and shorter response time compared to textile thermocouples, these sensors were not able to provide localized temperature measurements. Therefore, the use of textile RTDs to measure temperatures in micro or macro environments remains to be validated. The integration of Bragg reflector-type optical fibers to measure body temperature, which has provided high accuracy, is far from being applicable to a portable device, as such concepts require connection to fixed optical systems. The same observation is valid for concepts that have integrated heat flow sensors in textile structures. Being intended to be eventually integrated in clothing, textile temperature sensors need to be validated for mechanical or wash resistance in future work.

In addition, experts in flexible electronics have shown great interest in the development of temperature sensors on flexible polymeric substrates. Graphene layers deposited on flexible substrates have demonstrated RTD properties of very high temperature sensitivity. However, in an extensible configuration, the RTD graphene layers have shown thermal properties sensitive to mechanical deformations. Layers with RTD properties have also been developed on flexible substrates by depositing different types of dispersion (based on carbon, nickel oxide, silver complex, and mixing PEDOT-PSS with carbon nanotubes) using printing techniques. These heat-sensitive printed layers were able to ensure high temperature sensitivity while demonstrating low hysteresis in the heat–cooling cycles. The formation of composite layers on flexible substrates also allowed the fabrication of flexible temperature sensors. Among the various developments, composite layers based on carbon nanotubes have made it possible to obtain thermal sensitivities comparable to those of metals. However, in many studies on composite layers, electrodes based on precious metals such as gold have been used. Despite the advantages of some concepts for flexible temperature sensors, significant efforts are required to integrate them into clothing. From a general point of view, work on textile-integrated temperature sensors, textile sensors, and flexible temperature sensors seems to remain at the level of proof of concept with very few connected device demonstrators and even fewer prototypes of garments equipped with temperature sensors. In addition, the influence of various environmental parameters on the performance of these types of sensors remains unknown. Among the few studies on the design of garments with integrated temperature sensors, very few were dedicated to protective equipment, and almost all the work was carried out in the laboratory with tests on very few subjects. The effectiveness of these concepts has yet to be validated in operational environments. In addition, in most of these studies, conventional electrical wires were used for electrical connections or to ensure the transmission of collected signals. These types of structures containing electronics can be vulnerable to mechanical constraints during use and maintenance. The use of structures based on conductive textiles is to be expected in order to ensure a better mechanical resistance in use. Clothing equipped with temperature sensors that incorporate rigid thermistors embedded in textile fibers also require optimization efforts in order to reduce the impact of mechanical stresses on the quality of the sensor reading. The literature also mentions the influence of the fibrous structure surrounding the sensor on the reading [78]. Not only have few studies been carried out in this area, but an in-depth knowledge of the influence of the multilayer structures of various types of protective equipment on the performance of integrated sensors remains to be developed.

Among the commercially available products, flexible temperature sensors seem to be able to ensure high measurement accuracy and very short response times. Being mainly based on a very thin printed structure, this type of sensor requires relatively low power supplies of the order of microwatts. These products, which are currently manufactured on flexible polymeric substrates, are mainly dedicated to the fields of warehousing and logistics. In order to extend their application to clothing, research is still needed to ensure their reliability and durability in use. Very few products including garments with integrated temperature sensors currently exist on the market. These products are mainly dedicated to the protection of firefighting workers. These types of protective equipment, which include temperature sensors incorporated into their structure, can warn the firefighter when predefined temperature thresholds inside or outside the garment are exceeded. 

## 3. Heated Actuator

In recent years, the textile industry has proposed multiple solutions to offer better protection against the cold during outdoor winter activities. The use of various types of textile materials has made it possible to reduce heat loss from the body while ensuring the transmission of moisture from sweat through a garment that must remain water and wind resistant [158].

Despite technological advances in textile materials engineering, these types of garments still need to be improved. Indeed, most garments against extreme cold remain bulky by being based on multilayer fibrous structures taking advantage of the thermal resistance of textile materials, which depends mainly on their content of air trapped inside. In addition, highly insulating garments can sometimes limit body and arm movement and reduce manual dexterity, thus affecting individual performance. A feeling of discomfort may be particularly accentuated when clothing against the cold is worn in combination with other clothing [39,159]. In addition, it has always been difficult to correctly estimate the optimal clothing or number of layers to wear for sustained physical activity under varying environmental conditions [55].

### 3.1. Heating Garment Technologies

During intense activities in the cold, excessive perspiration, and consequently the humidification of the inner layers of the garment, can lead to a considerable decrease in thermal insulation, thus increasing the risk of cold-related injuries [160,161]. To offer a better level of comfort and higher endurance during activities in extreme cold, warm personal clothing has been proposed by actors of the textile industry. These types of garments also aim to offer more personalized solutions to individuals, incorporating additional technologies to their basic textile structure. The development of personal warming garments is of particular interest in a work context in order to protect workers against injuries directly or indirectly related to prolonged exposure to extreme cold [161]. These garments can be presented under four main categories according to their principle of operation: (1) Electric heating garment; (2) Fluid-flow-based heating garment; (3) Phase change material heating garment; and (4) Chemical heating garment [55].

#### 3.1.1. Electric Heating Garment

Among the different categories of personal heat garments, this study has mainly focused on electric heat garments, as they can provide heat in a sustained and durable manner throughout the performance of tasks in extreme cold, depending on the endurance of their portable power source. In addition, their structure incorporating a heating element could provide heat distribution in a space-saving, thinner cold protective garment [162,163]. The integration of electronic modules in combination with electric heating elements facilitates the creation of garments with adjustable heating levels that can even be adjusted to the individual’s personal situation [164].

#### 3.1.2. Fluid-Flow and Airflow Based on a Tubing System

In contrast to electric heating garments, fluid-flow-based heating garments are very bulky. Almost every example of this type of heating garment, based on a flexible tubing system for circulating liquid or hot air, requires an external energy source and fluid supply. In addition, the tubing system integrated into the garment makes it rigid, which may limit its usability during activities [55]. Nevertheless, due to their thermodynamic efficiency in heating the human body and the heat exchange capacity of specific areas, airflow-based heating garments have been successfully applied for medical surgery [165].

#### 3.1.3. Phase Change Material Heating Garment

Heating garments based on phase change material (PCM) also have important limitations despite a very interesting potential and many dedicated efforts. The most important limitation of this technology is its temporary heating effect. Although it is active during its phase change period, the release of heat ceases when the PCM, initially in a liquid state, solidifies with exposure to cold. Thus, in order to recover its heat source based on a phase change mechanism, it is necessary for the PCM to move away from the cold environment to reach its liquid state again [59]. It has also been reported in the literature that the integration of microencapsulated PCM into garments by coating and fiber-spinning techniques shows a low heating effect due to their low mass. In addition, their effect may gradually disappear when clothes are washed several times [60].

Since the thermal regulation capacity of textiles incorporating PCM is highly dependent on the amount of material deployed, the incorporation of PCM pockets in clothing generally leads to heavy clothing and may only be suitable for people for whom, depending on the activities, the extra weight is not a problem [60]. To address these problems, a great deal of research is underway. However, significant efforts still seem necessary to optimize the global enthalpy of phase change and the thermal window of the PCMs to ensure a sustained heat release effect [59,61] to meet the requirements of continuous hours of activity in cold weather.

#### 3.1.4. Chemical Heating Garment

Chemical heating garments are mainly based on chemical energy converted into thermal energy by oxidation during the reaction of chemical substances and are mostly used in diving suits to protect divers in cold water. The integration method remains primitive, because the reactive material placed in cushion-like packages is glued by an adhesive to the inner surface of the garment. The heat-generating chemicals are kept in separate compartments inside the cushion. When the user presses the pad, the barrier between the substance’s breaks, and the reagent mixes is generating heat. Although this system can use a mass of selected reagents to provide a highly exothermic chemical reaction free of gaseous by-products, the released temperature is difficult to control and of limited duration [166,167].

#### 3.1.5. Power Source

Despite durable heating throughout the duration of cold work, the low capacity of the batteries to ensure the proper functioning of the integrated heating system during long exposures to cold remains one of the major drawbacks of textile structures incorporating electric heating elements [38]. The rapid development of telephones and laptops has led to the availability of powerful and durable batteries that can also be used for auxiliary heating. However, these batteries may have disadvantages in terms of weight, space requirements in the garment structure, and the danger of overheating for some types of lithium-ion batteries [38]. The problem of efficient power supply for electrical functions is a major challenge in the design of intelligent textiles. Therefore, a lot of work has been undertaken to develop new methods for integrating energy sources [168] and textile structure batteries [169], while searching for new regenerated energy sources such as solar energy, sound wave power, human movement, or even friction energy from clothing [170,171]. Since this is a topic of important scientific interest that affects the entire field of smart textiles, the analysis of advances in flexible and portable energy storage for different types of electronic textiles requires a comprehensive study separate from the present one. Thus, the review of the literature concerning heating actuators has mainly focused on techniques for the development of electric heating elements that would offer a more efficient energy consumption with current portable energy sources, as well as a better heat input while ensuring flexible structures in order to better withstand mechanical stresses during the use and maintenance of personal protective equipment.

### 3.2. Conductive Heated Actuator

The functioning of electric heating garments is based on the Joule heating principle also known as ohmic heating [172], according to which the passage of an electric current through a conductor generates heat by affecting the integrity of the conductive body. According to Joule’s first law, the heating power of this principle is proportional to the product of the resistance of the conductive body and the square of the electric current flowing [173]. In early versions of electric heating garments, the heating element was based on an integrated electric heating wire or a 3D heating pad composed of electric wires or graphite elements [174]. Despite their advantages in terms of increased comfort in the cold, some users have pointed out disadvantages such as clutter, restriction of movement, overheating, and problems with the durability of the electrical wiring system during use and maintenance [55]. In addition, electric wire heating had technical limitations, as by restricting heating to the path of the wire, it failed to produce uniform heat over a selected area [163].

In order to circumvent these drawbacks, the design of heating elements based on conductive textile fibers or the deposition of conductive layers on the surface of textiles has been proposed by the scientific community [166]. Based on the technology used, these types of heating elements can be divided into five categories: (i) textile substrates coated with compositions based on silver particles; (ii) textile substrates coated with conductive polymers; (iii) heating elements based on carbon fiber or carbon-based compositions; (iv) heating textiles based on yarns of metallic compositions, and (v) hybrid heating textiles using simultaneously passive heating actuators and electric heating elements.

#### 3.2.1. Silver Coated Yarns

With the goal of solving the problems associated with the use of electrical wires as an integrated heating element in clothing, several works have attempted to apply metallic textile wires or wires made from metallic compositions [175]. In part of this work, heating elements were developed by sewing seams of metallic textile threads on the surface of various types of fabrics to simulate the embroidery process. In an analytical study, conductive yarns based on silver-coated Vectran^TM^ fibers (a type of aromatic polyester) [176] were sewn in serpentine shape on three stretchable knit fabric composed of cotton–elastane, polyester–elastane, and nylon–elastane in different variations. It was found that different levels of heat can be generated depending on the number of yarn passes, the spacing of the coil curves, and the type of knit, which also dictate the level of electrical power required [54]. Based on the knowledge developed on the spacing required between the coil curves and the number of yarn layers superimposed to obtain the best thermal response in terms of electrical power versus temperature [54], a prototype wrist heater providing a temperature range from 33 to 40 °C was developed using the same type of conductive textile yarn [177]. According to these studies, the creation of heating elements from embroidered conductive textile yarns could allow the generation of a much higher heat range than heating elements based on electric heating wires by applying the same power supply. According to the authors of this study, the influence of substrate fiber content, stitch configuration, and increased heating zone still requires further work [54]. Using the same technique, a heating element based on a silver wire was designed to provide heat close to body temperature with a power of 5 W supported by a portable 10 V battery with a capacity of 6000 mAh for 8 to 10 h of supply [178]. In addition, the power supply and saturation time for a given temperature were analyzed for a heating element designed by sewing a silver-coated nylon thread onto a polyester-based fabric to elucidate the power level required to achieve heat levels in the range of 27 to 43 °C [166]. All these results can contribute to the optimization of heating element design with embroidery techniques on an industrial scale.

As knitted fabrics offer flexible and stretchable structures, the creation of knitted heating elements has attracted particular attention from the scientific community in recent years [179,180,181,182]. In this context, the heat production of two silver-coated textile yarns with different electrical resistance, embedded in a traditional wool knitted fabric, has been studied by applying various levels of electrical tension for more than one hour. The results of this study showed that the total electrical resistance of the conductive knit fabric decreases significantly when the fabric is heated, as the linear resistance of the conductive yarns as well as the resistance of the contact points between the superimposed conductive yarns in the knit structure decreases with increasing temperature [182]. Studying the behavior of a silver-coated polyester yarn embedded in three different knit structures showed that the maximum equilibrium surface temperature of heated knit fabrics is strongly correlated with the energy consumption density. Furthermore, the maximum equilibrium surface temperature can be influenced by the knitting method, as the electrical resistance of some structures seems to remain more stable than others during the heating process [183].

Analysis of the design of weft knitted heating pads using three different types of conductive textile yarns embedded in two knitted fabrics of similar structure, but with different main yarns (acrylic and polyester respectively), showed that the electrical resistance of the conductive yarn and the composition of the knitted textile fibers surrounding the conductive textile yarn greatly influence the heat generated at a fixed supply voltage. The authors concluded that the acrylic yarn of the knitted fabric would have better heating and heat retention properties compared to polyester when using the same type of conductive yarn [184]. The influence of the design and the method of integrating the conductive yarn on the heat generated was also studied by integrating a silver-coated textile yarn into a fully knitted structure to compare it to stitches on the surface of a shoe insole. Depending on the design and the type of textile threads surrounding the conductive thread in the fabric, temperatures higher than the body temperature could be obtained with electrical powers as low as 1.7 Watt provided by portable low-voltage batteries [185].

#### 3.2.2. Metallic Textile Heating Elements

In addition to silver or silver-coated conductive textile yarns, other types of conductive textile yarns with a metallic composition were also considered for the design of heating elements. The study of the behavior of steel wire-based heating panels using single and multilayer steel wire integrated in clothes showed that the thermal effect obtained, and the time required to reach an equilibrium temperature at a fixed voltage, depended on the number of wire folds in the cloths [186]. In addition, the criteria for selecting conductive yarns for knitting an electric heater was explored using two types of steel yarn, two types of silver-coated polyamide yarn, and one polyester/steel blend yarn, each of which was knit in two patterns: (1) wool/polyamide knit with a 1 m long conductive yarn in three rows of loops; (2) a conductive area in a multiply knit fabric [187]. While finding that the maximum equilibrium temperature of the heating elements was influenced by the method of integration of conductive yarns, the authors concluded that an optimal heating element should contain conductive yarns with low electrical resistance and minor variations in electrical resistance to elongation, providing good temperature uniformity during the heating process while being mechanically suitable for knit structure. In this work, silver-coated polyamide yarns in a three-ply configuration were able to provide the most uniform heating zones while being technically suitable for a knit structure [187]. Analysis of the method of manufacturing flexible heating fabrics by integrating a copper coil filament between two pieces of flexible interlining fabric using the thermal adhesion process has demonstrated that reducing the copper wire spacing and the applied tension, while improving the thermal conductivity of the textile structure of the fabric, not only increases the temperature and heating rate but also helps to maintain the fabric at a uniform temperature [188].

In this context, a fabric with variable insulation properties was developed with a structure consisting of three fleece layers and two interlayers comprising copper filament spirals and Nitinol as a temperature-sensitive shape memory element. The inner layers, being heated by the passage of an electric stream, made it possible to increase the thickness of this part of the fabric during the heating process, thus ensuring the increase in the insulation of the fabric due to the increase in air present in the transverse direction of the fabric. The heat-induced physical change in the conductive spirals could be electrically adjusted, providing a means to control the overall insulation level of the fabric [159]. Finally, a heated knitted fabric was developed using a conductive elastic yarn of composite structure that included an elastane filament as a core and a steel filament combined with rayon fibers as a sheath wrapped around the core. Composite yarns of varying degrees of tension were embroidered on the surface of commercial knitted fabrics to obtain heating fabrics. According to the analysis of the thermomechanical behavior of heating fabrics based on conductive elastic yarn, despite reasonable cyclic stability in tensile tests, the temperatures obtained seemed to decrease with increasing tensile stress but still reached a stable thermal equilibrium after the application of the deformation [189].

#### 3.2.3. Mathematical Models for Metallic Heating Textiles

To facilitate the design of electric heating elements based on metallic textile wires, some research work has proposed mathematical models to better anticipate the behavior of the heating textile to be developed. In one of these studies, the thermomechanical properties of knitted structures based on silver-coated textile yarn were mathematically modeled as a function of the influence of the contact pressure at the structural bonding points on the heating level. Thus, considering the relationship of the electrothermal property of the material and the structural parameters of the knitted fabric, the resulting temperature and loop resistance of a knitted fabric of uniform width can be predicted. Practical validation of the model with a heated knitted fabric based on silver-coated polymeric yarn showed that the maximum temperature obtained at a fixed supply voltage would depend on the structure of the knitted fabric in plain, ribbed, and interlock stitches [190]. The same research group proposed a second model to predict the electrothermal behavior of a steel wire knitted structure, whose predictive accuracy was subsequently evaluated with experimental trials of integrating conductive steel wires into double-ply knitted fabrics of interlock and solid structures [191]. The results of this study showed again that the maximum temperature obtained and the reaching of a heating temperature equilibrium state at a given voltage would depend on the structure of the knitted fabric. Based on the analyses performed, steel wire-based heating elements can generate a greater amount of heat at very low power supply voltage, and therefore, its use would be recommended over silver-coated yarns when a high level of heat is required. This study also recommends an interlock structure for the design of heated knitwear due to better stability and higher temperature supplied compared to solid knitwear at the same electrical supply voltage [192].

Another theoretical model has been proposed to control the temperature of conductive knitwear of various courses and stitch yarns based on the quantitative relationship between the electrical resistance of a conductive knitwear and the temperature provided. According to this model, by knowing the initial resistance and thermal diffusivity [193] of the knitted fabric, as well as the applied voltage, it would be possible to predict the temperature provided by the knitted fabric. Experimental validation of the model with silver-coated yarns in the design of five woolen knitwear, with the same loop density but different loop arrangements, has demonstrated the dependence of the maximum temperature obtained on the type of loop arrangement [193]. Another model predicting the electrothermal properties of conductive knitwear was proposed by taking into consideration the thermal capacity of conductive and non-conductive yarns, the electrical resistance, and the thermal capacity of the heated knitwear. Experimental validation of the model, which also considered the coefficient of thermal conductivity, the mass, and the initial temperature of the fabric, showed that the coefficients of thermal conductivity and the thermal capacities of electrothermal fabrics depend on the type of conventional fiber used and the density of the loops of the knitted fabric. Experimental validation of the model using the integration of silver-coated yarns in three types of wool, acrylic, and cotton knitted fabrics with three different densities for each type of knitted fabric showed that the maximum temperature and time required to reach a stable heating temperature depend on the types of expanded textile fibers and the loop density of the knitted fabric [194].

These types of patterns have also been proposed to predict the design of heated woven fabrics. In order to express the relationship between various parameters of a heated woven fabric, an equation was proposed based on the resistance of the fabric, the heat output power, the DC voltage, the number of parallel conducting wires, the length of the single conducting wire, the resistivity of the conducting wire, and the cross-sectional area of the conducting wire. Validation experiments using the integration of silver filaments and silver-coated yarns in identical cotton fabrics concluded that the conductive yarns or filaments must be uniformly distributed in order to avoid overheating on parts of the heating fabric [195]. It was observed that silver-coated yarns would not be suitable for the design of heating fabric due to their poor thermal stability. In addition, silver filaments would be a better choice compared to steel wires in such structures to avoid wire breakage [195].

According to some of the models discussed, knowing the electrical resistance of a conductive tissue can greatly contribute to predicting its electrothermal behavior [193,195]. Therefore, theoretical models suggested by some experts to predict the overall resistance of a conductive knitted fabric can be taken into account. Studies such as the modeling of the resistance of conductive knitwear from the length-related resistance and the contact point resistance associated with the analysis of the electromechanical behavior of such knitwear [196], the modeling of the resistive network for conductive knitwear stitches [197], and the estimation of the resistance of conductive knitwear from a macroscopic view by considering the surface resistance of the conductive yarns [198], can be considered in such an approach to the design of a heated knitwear. In the same context, a derived simulation model has been developed to calculate the electrical resistance of a conductive woven fabric by considering its structure as well as the density and arrangement of the integrated conductive yarns. Once the radius of the warp yarn and the resistance of a unit of conductive yarn were known, the electrical resistance of the conductive woven fabric could be calculated. By validating the model using the integration of a silver-coated nylon wire in three woven structures with different weft density and constant warp density, the study demonstrated that for the same fabric size, the electrical resistance can be adjusted by controlling the fabric structure and the arrangement of conductive wires [199].

In order to facilitate the design of a heating element in a textile with a versatile design, and to overcome the technical challenges related to the integration of a conductive wire in a textile structure, coating techniques have been deployed to form conductive and heating zones on the surface of textile substrates. The deposition of a silver particle-based conductive ink on the surface of one polyester/cotton fabric resulted in a heating element that provided a maximum temperature of 33 °C with power supplies as low as 1.4 Watt and a time of about 10 s to reach the equilibrium heating temperature [200]. In a similar work, the deposition of a dispersion containing silver nanofilaments on a cotton woven fabric created a heating zone that could provide 50 °C heat at an applied power density as low as 0.05 W/cm^2^. Despite such performance, due to the relatively low environmental stability of silver nanofilaments, the developed heating fabrics lost their performance after two months of storage under ambient conditions. In addition, the created conductive layer was damaged during washing, and its thermal performance was significantly reduced [201]. In order to take advantage of the benefits of using silver nanofilaments in the design of a heating element, techniques such as the one proposed for the fabrication of heating membranes based on nanosilicon carbide and thermoplastic polyurethane covering the silver filaments [202] should be considered. Although these types of membranes may offer good thermal stability and better mechanical properties, their integration into textile structures remains to be explored.

#### 3.2.4. Textile Substrates Coated with Conductive Polymers

The formation of polymeric conductive layers on textile substrates has also been explored for the design of electrical heating textiles. The in situ polymerization of poly (3,4-ethylene dioxythiophene) p-toluene sulfonic acid (PEDOT-PTSA) on a polyester web by coating has allowed the development of a very flexible and lightweight heating textile with a durable and high heating potential that still required high supply voltages [203]. The deposition of a polypyrrole coating on a nylon-based knitted fabric was also used to create a textile heating element. However, voltages as high as 18 volts were required to generate temperatures in excess of 45 °C. In addition, the provided temperature appears to be altered during the elongation of the fabric [191]. Vapor-phase polymerization of poly (3,4-ethylene dioxythiophene) on a cotton fabric has made it possible to develop a heating element that can reach 28 and 45 °C with voltages of 4.5 and 6 volts, respectively. By means of a vapor-phase post-treatment for the deposition of a protective layer against moisture, it was possible to achieve better protection of the polymeric heating element against abrasion and mechanical deformation. According to the analyses performed, cutting, sewing, and partial weaving would not appear to alter the electrical conductivity and electrothermal responses of the heating layer [204]. Although these types of developments are very interesting, due to the technical challenges and high cost of scaling up vapor deposition techniques to meet the high-volume production requirements of the textile industry, it is difficult to envisage soon the use of vapor deposition processes to create textile-based electronic components [205].

#### 3.2.5. Heating Elements Based on Carbon Fiber or Carbon-Based Compositions

Carbon fibers are also very interesting candidates in the design of electric heating textiles because of their good thermal efficiency and ability to generate uniform heat quickly [206,207]. Allowing a very high rate of electricity conversion, carbon fibers can promote the design of heating elements with versatile surface temperatures depending on the desired design while providing an average lifespan of up to 100,000 h [55]. Examples of work in this context are the development of a heating element in the form of a composite layer based on recycled carbon fiber in a polyurethane resin producing heat ranging from 26 to 96 °C [208], the development of an anti-icing/de-icing device with the integration of a carbon fiber composite laminate in a multilayer structure requiring electrical currents of 2 to 4 amps to provide the desired electrical power density [209], and the evaluation of a carbon fiber-based electric blanket to warm patients during abdominal surgery, demonstrating a performance equivalent to that of forced hot air heating technologies and superior to that of hot water circulation mattresses in tests conducted in the hospital environment [210].

A few studies have also been devoted to the use of carbon fiber-based heating elements in the design of electric heating garments. The evaluation of an electric heating vest with a carbon fiber-based heating element on a thermal manikin in a cold climate chamber has shown that the application of too high temperatures can lead to a reduction in heating efficiency due to a significant loss of heat to the environment, thus demonstrating that the heating power should be adjusted according to the external temperature [211]. The influence of ambient air velocity and the influence of the suit of clothing worn on heating efficiency was also studied by testing an electric heating vest, equipped with six carbon fiber-based heating elements, on a thermal manikin. The combination of the vest with knitted underwear and a military uniform in different orders demonstrated that the order of the clothing combination can significantly influence the heating efficiency. Indeed, the best heating efficiency was obtained when the heating vest was worn as a middle layer in the middle of the other clothing. It has also been found that the heating efficiency of the heating vest decreases with increasing cold air velocity [212]. The efficiency of an electric heating garment containing seven carbon yarn-based heating pads was compared to that of a heating garment containing 14 PCM pockets during tests conducted under identical conditions using a thermal manikin operating in the thermoregulatory model control mode. According to the analyses performed, the electric heating garment can show a more efficient heating power and a significantly higher total thermal insulation compared to the PCM at low airflow velocities, whereas no significant difference was observed at high airflow velocities [162]. In addition, the analysis of different methods of applying carbon fiber in the design of an electric heating garment has shown that the use of carbon fiber can lead to a rapid temperature increase as well as a high recovery rate when disconnected from the power supply, so that such a heating element has the necessary characteristics for precise temperature control. Based on the results obtained, it was also recommended to take into consideration the human body heat dissipation principles and that of the garment surface in the design of the garment as well as a sandwich-type heating element design to promote better heat input [213]. Despite the advantages of a carbon fiber-based heating element, its integration into clothing still requires further work to optimize its resistance to washing [213] and energy consumption [208,209,211,213].

#### 3.2.6. Efficiency of Heating Clothing Based on Yarns of Metallic Compositions

In parallel with the numerous studies dedicated to the development of textile electric heating elements, some work has also been devoted to the evaluation of the efficiency of electric heating garments. The evaluation of a heated sleeping bag incorporating heating fabrics in the foot area on a thermal manikin, and subsequently on eight human subjects in the controlled conditions of a climatic chamber, has demonstrated the capacity of such a concept to keep feet and toes warm throughout the test period [214]. The optimal operating conditions for a heated glove with heating elements attached to the back of the layer adjacent to the fingers were determined by testing under controlled laboratory conditions. To this end, the study attempted to identify the heating power required to maintain the finger temperature above 15.6 °C, which is known as the minimum ergonomic design standard for space suits [215]. In a similar work, the evaluation of the performance of an electric heating glove on a thermal hand model identified the electrical power required to maintain thermo-neutral skin temperature of the hand during exposure to extreme cold. According to the observations, three additional watts was required to maintain the thermal comfort of a hand in moderate wind compared to a calm air circulation at −10 °C. This study also concluded that finger dexterity may also depend on the structure of the heating element and its flexibility as well as the glove configuration and fingertip design [161]. Another study evaluated the ability of an electric heating vest in warming up and improving the performance of elite sprint swimmers. Skin thermal imaging and measurements of tympanic temperature, heart rate, thermal comfort, and thermal sensation of male participants wearing a heated vest followed at a swim session showed a real beneficial warm-up effect compared to a group of unheated participants. However, no significant effect was observed for the female swimmers tested, suggesting a sex difference with possible links to gender differences in perceived discomfort [216].

In order to offer more comfort and ease in the execution of tasks during activities in the cold, clothing allowing control or self-regulation of the temperature has also been studied. In this context, a vest with temperature control capability was developed by combining steel wire-based heating panels, in several configurations from one to four layers, with a digital temperature sensor and a microcontroller. These components, being worn on a carrier, were subsequently attached by means of Velcro strips under a multilayer cotton/polyester/polyamide garment. A user interface on an external handheld device was also used to control and display the temperature. In a self-regulating temperature mode, the heating circuit was activated by the microcontroller if the value measured by the temperature sensor fell below a preset value [217]. Evaluation of this garment with a copper thermal manikin in a cold climate chamber showed that the maximum heating temperature would depend on the number of folds in the panels. According to these analyses, the single-layer heating elements could operate longer, while the power supply period became shorter for the high number of panels due to the lack of power supply. By comparing different types of batteries of identical capacity, the authors also concluded that nickel-metal hydride batteries would be more appropriate for cold environments with an instantaneous heating effect, while for circumstances requiring continuity, lithium-ion batteries providing stable heating would be more advantageous [217]. The effectiveness of a glove comprising an electric heating element and a temperature controller measuring the T_s_ of the fingers was examined by recording the thermal sensation of human subjects wearing the gloves in a climatic chamber. The results showed that such a glove would maintain the temperature of the back of the hand and fingers within a comfort zone. The tests showed that in addition to improved thermal sensation and comfort in the fingers, the thermal sensation and whole-body comfort sensation increased slightly with the use of electrically heated gloves in cold weather.

By applying a power switching method based on the self-monitoring of the heating element temperature, a heating textile with the ability to quickly reach various temperature levels, having a uniform temperature distribution band and ensuring the maintenance of the defined temperature, was developed. To realize such a concept, copper-coated polyurethane filaments were embroidered on a cotton fabric to design the heating element and an RTD-type temperature sensor. To ensure temperature self-regulation, an on–off control system referencing the temperature in real time was used to maintain the target temperature in the embroidered circuit, independent of the internal microclimate and external climatic conditions, as well as the battery voltage level [56]. In addition, an analytical study carried out an experimental characterization of the design parameters of a self-regulating heating garment [164]. For this purpose, a heating actuator based on serpentine stitching of silver-coated filaments was integrated into a three-layer garment comprising the heating element formed on the knitted base layer, a layer of aluminum foil in the center to improve heat retention, and a textile cover layer on the outside. In order to study the temperature control system, the garment was developed in three versions: (1) no control circuit; (2) the self-regulating garment with closed-loop T_s_ feedback using thermistors placed at various locations on the skin and a control system based on a microcontroller; and (3) the self-regulating but user-controllable garment with control of the thermistor feedback to maintain the internal temperature of the garment at a desired level and the use of an additional potentiometer to allow the user to control the set value of each actuator. According to the analyses of this study, total temperature self-regulation may be inadequate in complex thermal environments, indicating the need to consider ambient and body thermal effects in the thermal management of the temperature self-regulating system. By placing control of the system in the hands of the wearer, the self-regulating garment could overcome some of the challenges associated with complex environments by relying on the thermal sensation of the wearer [164].

#### 3.2.7. Hybrid Heating Textiles

Some studies have also looked at the combination of electric heating elements and functional heating materials to ensure better energy efficiency. In one of these studies, the influence of the use of phase change materials on the energy consumption of electrically heated garments was investigated [218]. For this purpose, several configurations of the same garment were developed by associating, or not, an electric heating element with a PCM-coated layer. Tests carried out on the different versions of the garment using a bionic skin model at 33 °C in a climatic chamber at −15 °C showed that the association of an electric heating element with a layer containing PCM can considerably optimize the distribution of heat in the garment, thus improving the thermal protection performance of the garment. In addition, the PCM coating with a melting point of 27 °C allowed the implementation of a self-regulating temperature mechanism whereby when the temperature produced by this layer fell below 27 °C, the conductive fabric was automatically energized, and conversely, when the temperature exceeded 29 °C, the conductive fabric was switched off. Such a hybrid configuration also resulted in energy savings of about 30% with the temperature control process [218].

By using textile fibers, such as cotton, polyester, or acrylic, containing metals of ceramic compounds (e.g., platinum, alumina, or silica derivatives), fabrics with the ability to absorb, reflect, and emit far-infrared waves have been developed. Using such potential, heating elements have been proposed for protective clothing against cold in recent years [160]. Some commercial products claim that their technology can capture thermal radiation emitted from body heat and then, by reacting as a reactive mirror, use thermal far-infrared rays to reflect energy back to the body [219]. The analysis of the integration of far-infrared wave reactive heating panels has shown an effect of local heat, but it is not enough to increase the temperature of fingers and toes during physical activities in the cold. However, their association with electric heating elements could still contribute to an optimization of energy consumption in electrically heated clothing [220]. In addition, a very recent study has proposed a dynamic exploitation of infrared radiation in textile structures in order to create thermal effects that are adaptive to the environment. Thus, a textile with dynamically adaptive optical properties, allowing the regulation of thermal radiation, has been designed with a structure composed of elliptically shaped dimorphic fibers of triacetate and cellulose. The fibers fused side by side were knitted and subsequently coated with multiwalled carbon nanotubes [221]. By arranging the electromagnetic spectrum and wave propagation of thermal radiation by controlling the distance-dependent electromagnetic interactions between the conductive elements of scales less than or equal to the desired wavelength, it was possible to create an adaptive aperture of IR radiation in the textile depending on the thermal response of the body against cold or in warmth with an inverse physical effect [221]. According to the authors, further research is needed to optimize the observed triggering effect and to address cost and human testing concerns.

### 3.3. Commercial Warming Clothing

The study of commercial products for heating actuators was mainly oriented toward electric heating products for sustained heating. On the other hand, few or no products were identified in the other three categories of heating garments, i.e., those based on fluid flow, phase change material, or chemical heating garments.

Indeed, commercially available electric garments use different technologies. Five types of technologies were defined in this study to classify companies and/or products, based on the review of scientific literature and information found on the websites of these heating product companies. The five types of technologies are conductive heating elements, electric heating wires, carbon fiber-based heating, graphene layer-based heating, and Positive Temperature Coefficient (PTC) conductive layer technology. Some types of technology such as conductive heating elements have been deliberately defined as quite generic, as it is often very difficult to know exactly what the technology of many products on the market consists of, as the information available on websites is often not very detailed, sometimes insufficient, or confusing. A sixth technology has been added but contains only one product that is distinct from the others. It is a face mask that warms and humidifies the air inhaled, which was first developed for people with asthma or respiratory disease (ColdAvenger). Table 2 shows the number of companies listed for the different heating technologies and the types of products they offer.

Nineteen of the companies identified were classified under the generic category of conductive heating elements because they provide very little detail on the heating technology used in their products on websites or data sheets. However, images, videos, and promotional interviews of these companies suggest that, for example, the heating elements used by some companies are based on conductive textiles (Makita, Zanier, Soleno Textile), conductive elastomers laminated to textiles (New Textile Technologies—NTT, Loomia), printed heating elements (Digitsole, which offers insole heaters, Conductive Transfers), or heating elements knitted into clothing (Odlo, Myant & Helly Hansen).

Among the 13 companies analyzed that use electric heating wires, Interactive-wear produces heating textiles made with embroidered, single-layer Litz yarns that meet automotive quality standards to minimize the risk of hot spots. The Volt Smart Yarns company manufactures garments and heating textiles using different types of yarns (stainless steel, copper, nickel, etc.). The other companies in this category produce heated clothing, but it is often difficult to have the details of the heating wire technology. For example, Gyde Wearable Technology announces that these garments contain micro-wire heating zones, but they do not provide more detail. Gerbing sells a jacket with a heat output of 77 watts, making it the warmest product Gerbing has to offer. The jacket contains more than 30 m of MicrowirePRO^®^ heating wire in seven different heating zones (front, back, collar, sleeves) for complete body heating. It also has three outlets that can power heating gloves (at different temperatures than the rest of the jacket), pants, and socks.

Twenty-nine of the companies classified use a carbon fiber-based heating system to provide warm clothing such as jackets, vests, and shirts, as well as beanies, socks, or gloves. Duran, a Chinese company, claims to be the first company to have developed and commercialized carbon fiber heating yarns, and it is the only one capable of precisely controlling fiber strength during production to ±5% (per meter). According to their website, Duran holds 14 international patents and 18 national patents for electric heating products. A heating element made from carbon fibers can quickly reach the desired temperature in just a few seconds. It can even have a long lifetime, up to more than 1000 working hours, as for the Arris company’s heating vest with a constant temperature of 40 to 80 °C. For example, the information provided by the manufacturers’ website shows that Verseo uses very thin, stretchy carbon fibers, that Heated Gear and EGEVogue use a silver-coated polyester thermal lining to reflect heat in addition to the carbon fiber heating elements and benefit from a hybrid system, Colcham offers a safe heating system by providing short-circuit protection, and that Octocool claims to use more carbon fiber (60 to 80% more) in their heating jacket than other competing brands. Vinmori, a Chinese company, states on its website that it uses Toray carbon fiber from Japan to improve the emission wavelength of the heating panel to reach values of 3 at 14 μm, with most heating wavelengths between 2 and 10 μm and can cause greater heat dissipation. This company also uses a temperature control system that ensures that the heating panel can quickly reach its highest heating temperature in 3 min. In addition, a built-in NTC-type thermistor temperature sensor can automatically detect the panel temperature every 0.3 s. Thus, the heating panel can operate at the specified temperature, with the accuracy of 0.3 °C regardless of the external ambient temperature, and avoid excessive temperature that may expose the body to the risk of burns. In order to ensure a firmer and safer circuit, carbon fibers wrapped in a polyester film were considered. In addition, to ensure the electrical connections in its products, the company has favored the use of conductive wires with a thermoplastic elastomer resistant to low temperatures in order to maintain the mechanical strength of the wire and avoid its breaking even at −40 °C.

Three of the companies listed use one heating technology based on graphene layers (Firefox Heated Coats, AGPTek, Vulpes). According to the available information, this technology allows products that are light, resistant (to traction, bending, friction, cold, washing), durable, and offering good thermal performance. Graphene elements, in addition to allowing an equal and efficient distribution of heat, can be used safely in various conditions of temperature, humidity, or exposure to water and under high mechanical stress.

Two of the listed companies, Nuova Heat and Nissha GSI Technologies, manufacture electronic textiles based on PTC technology for applications in the medical and industrial fields, such as aerospace, automotive, military, consumer goods, etc. The thermoregulatory PTC technology is based on a high temperature expanding resin layer that is loaded with conductive particles (often carbon). Such a film can control the temperature itself by regulating the heating power using its electrical resistance response to temperature, which varies with the expansion of the resin causing the distance between the conductive particles to increase. At low temperatures, its resistance is lower, so its heating power is greater, resulting in a rapid increase in temperature. As the temperature rises, its resistance increases, and therefore, its heating power decreases, thus controlling the temperature (Okutani, Yokota, Matsukawa and Someya, 2020). Once deposited on a textile structure, the PTC layer heats evenly over the entire surface of the textile and self-regulates to a specific temperature, thus reducing the possibility of overheating the garment. According to manufacturers, products using PTC technology have the potential to be safer and even more efficient compared to those using more traditional yarn or carbon fiber technologies. PTC heating elements from Nuova Heat, a U.S.-based company, are manufactured by depositing a conductive ink printed on a nylon fabric containing traces of silver as electrodes that can reach 55 °C in a few seconds with the passage of a 9 V direct current. Only one company identified uses a technology based on a conductive carbon-based PTC layer. This is Kinesix Sports, whose product, which allows self-regulation of the heating temperature, is described in detail below. This company uses flexible, lightweight heating pads made from PTC-type carbon ink encapsulated between two extra-thin layers of polyester.

In general, about 50 companies offer clothing and accessories that include heating technologies, mostly integrated in jackets and vests (sleeveless), but also in pants, body suits, gloves, socks, scarves, and beanies. Although 14 of these companies were only found on online sales platforms such as Amazon, most of them have a website where they present their products and features and sometimes explain the technology used. For jackets and vests specifically, two-thirds of the products listed have three heating zones, two of which are located on the chest and one on the upper back. In addition to these three zones, many products also offer heating zones on the collar to warm the neck, on the pockets to warm the hands, on the lower back and, for only a few products, on the sides of the body or on the arms. Most heated garments such as jackets and vests use a lithium-ion battery (4 V, 5 V, 7.4 V and 12 V), which allows the heating elements to provide heat higher than body temperature. In addition, many of the commercially available jackets and vests have USB ports that allow the battery to be used to charge mobile devices. Two-thirds of jackets and vests allow three temperature settings, for example, 25 °C/35 °C/45 °C for some products or 45 °C/55 °C/65 °C for others. These settings provide continuous heat for periods of time that can be, for example, around 15 h, 7 h, and 5 h for some products, or 4.5 h, 3 h, and 2 h for other products, depending on the temperature supplied and the power available. Usually, an LED control switch is integrated into the chest of the garment to allow the user to adjust and interpret the heat settings at different levels. Most commercially available products can be washed according to the manufacturer’s instructions. They are mainly aimed at the sports, leisure, or generic markets. A few garments and vests stand out because of their particular features or performance. For example, some companies offer heated shirts, jackets, or vests with plugs that allow the same energy source to be used to connect heating gloves (Warm & Safe Heated Gear, California Heat, Gerbing), heated pants, or heated socks (California Heat, Gerbing). Other companies offer independent heating zones to separately adjust the temperature of certain areas, such as the front, back, and hands (via garment pockets) (Arris), front and back (Vinmori), or body and hands (Ptahdus). Some companies offer continuous adjustments of the heating temperature via a variable switch (Warm & Safe Heated Gear) or with the help of a smart phone application (Odlo, Clim8, Vulpés).

In addition, this study identified three companies that have implemented systems that allow self-regulation of the heating temperature thanks to integrated thermal sensors that measure the temperature inside the garment or that of the skin. Clim8 proposes an intelligent thermal system integrated in a textile panel, in the form of a sweater adjusted to the body. This sweater is equipped with thermal sensors integrated in the fibers and controlled by a smart phone application. Once the temperature is set by the user, the sensors measure in real time the temperature of the microclimate, and the system activates when the temperature detected by the sensors is below the reference threshold and deactivates above this temperature. The mobile application of this system still allows manual activation and control of the garment heating. The company announces that the heating elements are positioned on the vital parts of the body. However, the available images and videos show that the technology seems to be present at least on the front and back of the sweater. Other companies such as Odlo and K2 also use Clim8 technology. Odlo has developed, with Clim8 and Twinery, the I-Thermic system integrated into a knitted sweater that can be worn alone or under a jacket. Although few details are provided on Odlo’s website, it seems that the heating elements are knitted in the shape of a coil. The company says that with this option for total control of the personal microenvironment, it is not necessary to wear an extra layer under the winter sports jacket. Equipped with a battery offering 4 h of autonomy, Odlo’s I-Thermic sweater seems safe, since the heating elements and software are set not to exceed 37 °C and stop immediately in case of higher temperatures, avoiding overheating.

In association with Helly Hansen, the Canadian company Myant has announced a line of active thermal workwear that provides thermal regulation for low-temperature environments. These garments feature an electronic textile layer and include a base layer top, leggings, socks, balaclava, and gloves. Equipped with textile heating elements and integrated temperature sensors, the system detects the skin temperature and the temperature of the microclimate close to the body to trigger a reaction by actively supplying heat through the textile to regulate the temperature. Being designed using advanced knitting technology, these workwears have a tailored design to better keep the sensors and actuators in contact with the body. Note that the company Myant, according to the information available on its website, seems to have the will to contribute to the future of work through smart textiles, artificial intelligence, and the Internet of Objects. In addition to a platform to measure the physiological parameters of workers with smart textiles, they want to be able to measure the environmental conditions (temperature, humidity, CO_2_ and methane levels, noise level, etc.) of a workplace.

Another Canadian company, Kinesix Sports, is working on the development of an intelligent heating jacket equipped with five thermal sensors capable of monitoring the temperature inside and outside the jacket in real time, and it includes 12 heating pads made from PTC carbon ink encapsulated between two layers of polyester. The ink used for the pads is specially designed to stop heating when the maximum temperature of 40 °C is reached, thus avoiding overheating. The system, based on a technology called ThermoAdapt, exploits artificial intelligence, more precisely automatic machine learning, to adapt to and anticipate temperature variations as the jacket is used. The heating pads, powered by an external battery, are in four independent zones of the jacket. The system constantly and independently adjusts and regulates each zone according to the temperature selected by the user. In addition, a thermal sensor located on the outside of the jacket can detect sudden temperature changes in the outside environment in order to instantly stop or activate the system. The four thermal sensors positioned inside the mantle, near each heating zone, help the system understand whether it is necessary to heat the entire body or only a specific part of the body. However, the system also allows the heating system to be activated manually if necessary. The heating pads are removable so that they can be easily replaced in the case of a malfunction.

Regarding an occupational health and safety application, this study also identified a few companies that offer products targeting workers in various industries, including construction, heavy industry, or all types of cold outdoor work (post office, airport runways, etc.). Some offer clothing that can be worn under a uniform or work clothing (Mobile Warming, Warm Fitness, Volt Smart Yarns, Techniche). Others offer high-visibility heated jackets, vests, or hoodies (Mobile Warming, Dewalt, Makita). Finally, five companies offer products dedicated to workers: Milwaukee, Dewalt, Bosch, Makita, and Myant-Helly Hansen (including an intelligent garment offering self-regulation of body temperature that was described above). Among the range of products for use in the workplace, Makita’s jacket provides 28 h of warmth with an 18 V battery.

### 3.4. Heated Actuator Challenges

Among the different categories of heated clothing designed to provide better comfort during activities in extreme cold, this study focused on electric heated clothing providing continuous heat within the limits of their energy sources, while offering the possibility of developing space-saving structures with a reduced thickness (Table 3).

Despite a very good potential, at the current state of technological advancement, PCM-based garments do not have the capacity to provide sustained and durable heat throughout a working day in a cold environment due to the temporary heating effect of PCM-based heating elements, the low thermal effect and durability problems of microencapsulated PCM coated on the textile, and the high weight and reduced sweat evacuation in PCM pocket-based garments. Therefore, significant work is still required to achieve a sustained heat effect from PCM garments.

In order to overcome the disadvantages of the conventional use of electric heating wires, heating elements based on conductive textile fibers have been developed in recent years. Within this context, several methods have been proposed to design heating elements based on metallic textile wires (fibers coated with a composition containing metallic particles) or based on metallic compositions (i.e., based on copper, steel, silver fibers, etc.). However, the analysis of the research work has shown that obtaining such textile heating elements requires the control of many parameters. Concerning the heating elements designed with the embroidery of metallic (textile) threads, the number of thread passages, the spacing between the threads, and the composition of the base fabric have an impact on the heating temperature and the level of electrical power required. Despite the advantages of heating elements embroidered with metallic threads in terms of energy consumption, research is still needed to better control the influence of the fiber content of the base fabric and the enlargement of the size of these heating elements. Despite the advantages of a flexible and stretchable structure of the knitted heating elements, their design is also a technological challenge. It has been shown that the thermal effect achieved in heated knitted fabrics depends on the type of conductive yarn, its mechanical properties, the structure of the knitted fabric, the knitting method, the composition of the textile fibers surrounding the conductive yarn in the knitted fabric, and the number of plies in a possible multilayer structure. It has also been shown that with the right design and conductive yarns with appropriate electrical resistance, knitted heating elements working with low power supplies could be developed. In a possible approach to integrating knitted heating elements in protective equipment, special attention must be paid to such parameters, in particular the structure and composition of the layers constituting the workwear.

In order to facilitate the design of electric heating elements based on metallic textile yarns, some mathematical models have been developed to predict the thermoelectric behavior of heating fabrics or knitted fabrics [223]. With these models, the maximum equilibrium heating temperature and the time required to reach it can be calculated from the thermal and structural properties of the fabric and the electrical characteristics of the conductive yarns. However, as these models have been applied to specific types of conductive yarns or fabrics, their applicability in the design of protective equipment with particular compositions and structures remains to be validated. From a general point of view, very little work has been done on the durability and characterization of the electromechanical behavior of electrical heating elements based on metallic textile wires. However, such technical information is necessary for the integration of these heating elements in protective equipment.

Heating elements with versatile designs can be formed on the surface of flexible substrates using coating techniques. Silver particle-based coatings ensure low energy consumption and very short times to reach the maximum equilibrium temperature. However, their low washout durability can be a very important shortcoming. In addition, coatings based on silver nanofilaments have poor stability in ambient air. Therefore, encapsulation techniques would be necessary to protect them in a possible integration process in protective equipment. Despite the flexibility and lightness offered by the coated layers based on conductive polymers and their ability to provide stable heat at high temperatures, they require a fairly high energy consumption and present certain failures from a mechanical resistance point of view. On the other hand, carbon fibers have been the subject of research work as well as numerous industrial developments in recent years. Indeed, due to their good thermal efficiency, rapid attainment of uniform heat, rapid recovery of the initial temperature when the power supply is switched off, and a very high electricity conversion rate, carbon fiber-based heating elements are ideal candidates for the implementation of precise temperature control. However, further research is needed to optimize the wash resistance and energy consumption of carbon fiber heating elements.

Despite the large number of studies dedicated to the development of new types of heating elements, little work has been done on the design or efficiency of heating garments. Furthermore, few studies have been devoted to the use of heating garments in a work context or to the development of protective equipment with heating elements. Indeed, most studies have been carried out in the laboratory with few human subjects. As some studies have highlighted a difference in the sensation of comfort expressed between male and female subjects when using heated clothing, more investigation is also needed in order to define the optimal heating conditions. Based on the results of previous studies, the impact of factors such as the combination of the heated garment with other clothing or environmental conditions on the performance of the heated garment in a work environment should be studied in order to obtain the best possible thermal performance. Extending the research on conventional (electric) heated gloves, the influence of the structure of the heating element as well as the design of a protective glove with heating elements on the dexterity of the fingers remains to be studied. 

In addition, the association of electric heating elements with far-infrared wave reactive heating panels or PCM-based heating elements to ensure a better energy consumption efficiency proposed in the literature is one of the concepts that remains to be explored in the structure of protective equipment and an active work context. The association of temperature-sensitive shape memory materials with electric heating elements allowing the placement of textiles with insulation properties that vary with the level of heating, used as a means to control the overall degree of insulation of the fabric, is another concept that could be applied to protective equipment to provide better protection to the worker.

As with the literature review, research on products containing heating actuators has focused mainly on electrical heating garments. As this is a dominant technological trend and there is strong industrial competition between the various players in this sector, several companies did not provide any information regarding the technology used in the design of the electric heating elements of their products. Despite all the known limitations of electric wire-based heating elements, this technology still seems to attract the attention of a significant number of manufacturers because of the simplicity of its implementation. However, because of the advantages of using carbon fibers in the design of heating elements, this technology seems to be the new trend among manufacturers. Positive temperature coefficient (PTC) heating elements are also a growing category of technology because of their ability to self-regulate the heating temperature to a specific level. Due to numerous advantages such as quick response to temperature change commands, good thermal efficiency, uniform heating capacity, etc., carbon fiber or PTC-based heating elements can form the basis for future work on the integration of heating elements in personal protective equipment.

The analysis of commercial products has also shown that more and more warming garments allow several areas of the body to be heated, while enabling the temperature to be varied using an integrated control switch or wireless temperature control. Although a number of these types of electric heat garments are also intended for workers in different industries, the heating zones are fixed, and temperature settings are often limited to three levels and restricted temperature ranges. Not only are these products unable to provide a customized solution, but such structures can also present serious overheating problems when used during intense work activities. Therefore, the few products offering independent heating zones and allowing interruption or adjustment of the temperature of each zone separately, as well as garments offering temperature control using a variable switch may be of interest for adaptation to use in work environments. In future work, it may be important to study the impact of independently controlling the heating temperature of different parts of the body based on the heat loss of different parts of the body, which can vary considerably depending on physical activities performed and the type of equipment worn (helmet, harness, etc.).

Thanks to advances in portable technologies, a limited number of products that allow self-regulation of the heating temperature using integrated thermal sensors that measure the microclimatic temperature inside the garment or the skin have been launched on the market over the last two years. As this is a very recent technology, the effectiveness of such systems, as well as their impact on the physiological aspects of people performing cold work tasks, remains to be studied. In addition, the integration of self-regulating temperature actuators into personal protective equipment structures requires significant research efforts.

## 4. Cooling Actuator

Among the various means of intelligent thermal management, cooling actuators are the technological solutions most dedicated to the occupational health and safety application. Since the evaporation of sweat is the most efficient way for the body to cool down, it is practically impossible to do so when wearing fully enclosed protective equipment such as protective clothing against chemical, biological, radiological, or nuclear CBRN hazards [224]. In addition, the weight, stiffness, and multilayer design of many protective equipment such as those used by firefighters can increase the energy cost associated with wearing them during work [225]. Increased metabolic heat production and decreased body heat dissipation under the protective layers of such equipment can lead to decreased physical performance and increased risk of heat stress [226]. In some workplaces, it is not economically viable or practically impossible to make environmental changes to reduce ambient temperatures. Such cases include hot open environments and large workplaces such as deserts, steel mills, smelters, mines, and metallurgical plants [58]. Due to the requirements for the design of protective equipment, small variations in thermal properties introduced in their design have had little or no effect on heat exchange with the environment [227]. As a result, personal cooling garments have been proposed to provide an effective method for cooling the body under protective equipment or in hot environments [58]. Based on microclimatic cooling focused on the regulation of body surface temperature, personal cooling garments have been deployed to promote the body’s heat exchange with the environment through the heat transfer by conduction, convection, radiation, and evaporation [228].

### 4.1. Cooling Garments Categories

Personal cooling garments can be divided into two main categories according to their passive or active cooling system. Passive cooling garments include conductive, phase change (PCM) cooling, and evaporative cooling elements. Active cooling garments include thermoelectric, air ventilation, and circulating fluid coolers [57,229]. While the performance of passive cooling garments is likely to be greatly affected by environmental conditions, user activity, and the resulting generation of body heat, the effect of active cooling garments is relatively stable and less likely to be affected by environmental conditions [57].

### 4.2. Phase Change Material Integration in Cooling Garments

The present study was particularly interested in the analysis of active cooling garments that could provide sustained cooling, depending on their power sources. For passive cooling garments, the detailed analysis focused instead on conductive and evaporative cooling elements. As the integration of PCM cooling elements in garments has been studied in various studies, their state of the art has been widely documented [59,60], demonstrating that their application for persistent cooling requires significant research efforts.

Indeed, PCM cooling garments use the energy of latent heat to maintain the microclimate temperature close to the skin temperature. The cooling mechanism is based on the melting of a substance going from a solid state to a liquid state that allows the absorption of body heat transported to the skin surface. This type of cooling is effective when PCMs change from their solid to liquid phase. Therefore, the cooling effect is only effective within a narrow temperature range of the microclimate that triggers a phase change of the material [57,58]. Being a relatively simple system to deploy, the PCM-containing layer requires direct contact with the skin for a superior efficacy [57]. Since the efficiency of the thermal effects and their duration depend mainly on the latent heat storage capacity of the PCM itself, the quantity of PCM used is the main factor affecting thermal efficiency and the amount of energy absorbed or released at the time of phase change [230]. In order to achieve good thermal productivity, cooling elements in the form of pockets containing PCM were integrated into the cooling garment design. However, these types of pockets have some disadvantages such as obstruction to sweat evacuation or the stiffness and weight of the pockets, reducing the mobility of the user [58]. Indeed, the conventional duration of the cooling effect of PCM embedded in the textile is 15 min and can rise to a maximum of 2 h depending on the number of layers, the mass, and the area covered by the material, but at the cost of a significant increase in the weight of the garment, which will increase the energy expenditure of the individual [230].

To overcome these problems, experts proposed the coating of microencapsulated PCM on fibers or fabrics. However, as the amount of microencapsulated PCM inserted into textiles to ensure thermal productivity increases, the permeability (to air, vapor, and moisture) of the fabric decreases. In addition, as the stiffness of the fabric increases, its softness and flexural strength decreases. Furthermore, despite efforts to improve the resistance of PCM microcapsules to washing, abrasion, and high temperature, it has been reported that the material can lose up to 60% of its heat storage capacity after a few washes [230]. With respect to their integration into personal protective equipment, the flammable structure of some PCMs would not be suitable for work environments in direct contact with fire [58,230]. In addition, the thick and sometimes multilayered structure of personal protective equipment can negatively influence the effectiveness of the PCM-based element by delaying the release of latent heat [59]. Although several research groups have attempted to overcome some of the limitations of PCM-based cooling elements by chemical, physical, and mechanical means such as improving their stability during phase change, the cooling capacity of this technology remains relatively low [59].

### 4.3. Active Cooling Actuator

Personal cooling garments were initially developed to reduce the effect of thermal stress in hostile aerospace and industrial environments. Even if the first developments date back 50 years, research on the optimization and effective integration of these devices into clothing continues [58].

According to a first observation, a large part of the work on cooling garments is dedicated to fluid cooling garments (FCG). These garments employ a conduction cooling system that circulates cooled fluid inside a garment close to the skin surface. The cooled fluid can be a liquid such as water or compressed or ambient air. A network of pipes attached to the inside of the garment conducts the cold fluid through the garment and returns it to a cooling device after conduction heat exchange with the body. The cooling system typically contains a pump, a reservoir, and a control valve [231]. To date, the main application areas for these garments have been in space suits during extra-vehicular activities, sunlit aircraft cockpits, military operations, mining, and the warm-up or cooldown phases of elite athletes. They may also be advantageous for workers working in vehicles, as it is convenient to attach the refrigeration unit or compressed air system to them [58]. As this technology has been in use since the 1960s, a significant part of the last ten years of research on FCG has been dedicated to the study of the physiological response of the body under cooling conditions.

Since the conduction mechanism requires direct and continuous contact between the tubular network of FCG and the skin, the contact pressure and the uniformity of tube distribution could have a major impact on the heat exchange between FCG and the body. In order to promote this heat exchange, the inner textile layer of FCG to be worn close to the skin should have good thermal conductivity and provide good moisture management, while ensuring a good fit to the body and good tactile properties. In addition, the material of the tubes, their thermal conductivity, overall length, internal diameter, and wall thickness, as well as the flow rate and temperature of the circulating fluid are other parameters that influence the effectiveness of FCG. The distribution of the tubes is another important factor affecting the efficiency of FCG in cooling different areas of the body or intermittent and regional cooling. In addition, liquid and air-cooled garments are limited by their required power and total system size [58,231].

#### 4.3.1. Fluid Cooling Garment Design

As a result, several studies have been devoted to the optimization of FCG design in recent years. In this context, the comparison of two water FCGs of identical tubular networks but different textile structures on a thermal manikin have shown that the type of knitted fabric used to contain the tubes greatly affects the heat transfer in the garment. For example, double Jersey fabrics with naturally curved structures that accommodate the tube would provide a better cooling effect than single Jersey fabrics that require additional material, such as foam interlining, to accommodate the tube, leading to a lower heat transfer coefficient [232]. As an interruption of liquid flow can occur with the compression of the tubes integrated in the FCG garment, an optimization of the integration of the tubes into the textile was proposed by inserting them directly into the modified structure of a specific knit fabric that included a spacer containing channels produced during the knitting process. This development, which aimed at a better ergonomic contribution, remained to be validated on human subjects or thermal manikins [233]. Based on a series of sequential tests evaluating the physiological and psychological sensations of the individual, the arrangement and fixation of the tubes, the textile materials, and the assembly of the piece were progressively improved in order to propose a process for the design and conception of an FCG garment hood. Despite the proposed methodology, the study remains limited due to the testing of only one male subject [234].

As part of the development of FCG for a space suit and in order to determine whether the capacity of the mechanical pump was appropriate for this system, the heat removal capacity of the system was determined by applying a thermodynamic heat exchange model. The equation was subsequently validated by comparing the theoretical values with the values obtained by thermocouples recording the entry and exit temperature of the FCG suit [235]. In another theoretical study, a model considering the metabolic heat, convective heat flux, and radiation heat flux of the environment was set up to analyze the effects of different factors in the performance of FCG in a warm environment and to identify the main limitations preventing optimal performance. Model validation tests on a thermal manikin and the thermal resistance analysis demonstrated that the flow rate of the liquid circulation had a greater effect on the thermal resistance between water and the environment than between water and the skin. According to the same analyses, the coolant flow rate and the ambient temperature would greatly affect the duration of action of the FCG garment [236].

Some experts have proposed the presence of a cooling control system in FCG garments to adjust the temperature and flow rate of the coolant circulation according to the microclimatic temperature changes close to the skin. Thus, with the decrease in metabolic activity, the wearer of the garment would not experience undesirable body heat loss and thermal discomfort due to excessive cooling [229]. Indeed, some work has focused on the development of devices to control the flow rate of fluid circulation, since earlier studies on human subjects had shown that intermittent cooling could reduce the effect of thermal stress in a manner equivalent to continuous cooling by FCG while allowing moderate peripheral cutaneous vasodilatation to be maintained compared with the cutaneous vasoconstriction of over-cooled skin [237]. Such methods of intermittent cooling, involving a 2-min cycle of operation and 2 min of shutdown, have also been compared to continuous cooling or alternate cooling based on a change in the direction of flow every 2 min in a water-based FCG through tests performed on a thermal manikin [238]. According to the results of this study, the risk of overcooling is very low with alternate cooling, which would also have increased system efficiency by more than 50% compared to continuous cooling. However, intermittent cooling was not considered to be very advantageous, as some of the potential efficiency gains from this mode could be lost due to off-cycle losses [238]. The controlled cooling mode of a water-based FCG, activated at a T_s_ of 34.5 °C and deactivated at a T_s_ of 33.5 °C, demonstrated longer periods of heat stress management compared to continuous and intermittent cooling modes [239], and it was included in an analytical study examining T_s_ feedback to activate an FCG when T_s_ was in the range of 33 to 35 °C [240]. Thus, it was demonstrated that in addition to reducing energy requirements, control of an FCG by the T_s_ of the individual could reduce thermal stress in the same way as constant cooling [240].

As in humid environments, water circulation in the space between the skin and the dense layers of personal protective equipment can lead to the appearance of steam and cause skin burns; thus, researchers have proposed water-based FCG garments with a self-transpiration capacity induced by oozing water from 20 pores in the tubular network for cooling with heat loss by evaporation [241]. The self-permeable FCG garment was designed with a tube attached to the outer surface of the garment to improve moisture absorption and was subsequently tested by a few male subjects to demonstrate that such a garment could effectively lower T_s_ without increasing the moisture content of the garment. However, the cooling effect was delayed until a sufficient dose of water was released and evaporated [241]. The same concept was taken up in a second study that proposed the presence of only 10 pores in the tubular network for evaporative cooling combined with control of water vaporization by the individual as an additional evaporative cooling function. Tests conducted in a climate chamber on male subjects controlling the evaporation process in the garment with a control button demonstrated the ability of a controllable perspiration FCG in reducing T_s_ without causing an increase in garment moisture from the start of cooling [242]. Despite the great potential of FCG function control systems, all the research work analyzed was limited to validation tests in a laboratory environment.

More recently, nanofluids have drawn the attention of scientists due to their high rates of heat transfer, which allows them to be used in various industrial uses. A new class of nanofluids, “hybrid nanofluids”, has recently been used to further improve the rate of heat transfer. The current phenomenon particularly concerns the analysis of the flow and heat transfer of SWCNT (single-wall CNT) MWCNT (multiwall CNT)/water hybrid nanofluid with activation energy through a moving wedge [243]. However, this technology has not yet been used in the development of PPE.

#### 4.3.2. PCM-Based Suspensions as Cooling Actuator

In order to overcome some limitations on the use of cold water in an FCG garment with respect to the weight of the cooling tank or the influence of ambient heat on the water temperature, some research has proposed the use of other liquids to be circulated in the tubular network of FCG [244,245]. Evaluation of the use of microencapsulated PCM-based suspensions as a coolant in an FCG worn on a thermal manikin has shown that the inlet temperature, the flow rate, and the concentration of the microcapsules were the most influential parameters on the heat dissipation by such a system. With proper adjustment of these parameters, significantly better heat dissipation could be achieved with the application of a suspension of PCM instead of water. In addition, the use of a PCM suspension could improve the performance of the cooling garment without an apparent increase in pump power [246]. A laboratory-scale study of a liquid carbon dioxide cooling garment worn by male subjects showed that these types of FCGs were effective in relieving thermal stress by lowering the T_s_ and Trec values of individuals, thereby enhancing worker productivity in a hot, humid environment with a relatively lighter portable cooling system compared to similarly sized FCGs operating with cold water [247].

#### 4.3.3. Air and Gas Circulation as Cooling Actuator

FCG garments using air circulation in an integrated tubular network have also been the subject of recent studies. Examination of an air FCG garment with a stationary compressor generating dehumidified air blown through a tubular network covering certain body regions under a chemical protective suit has shown that such a device would significantly reduce the effect of thermal stress. Tests carried out on human subjects have also shown that with this type of clothing, working hours could be considerably extended [248].

**Figure 5:**
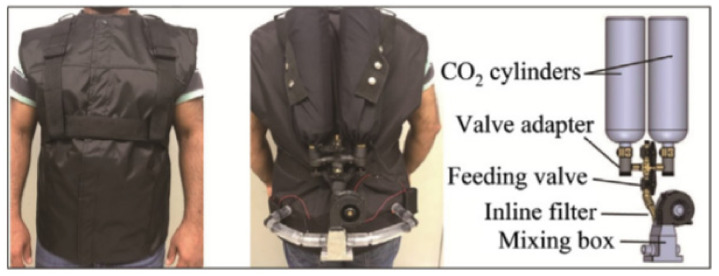
The air treatment system of the cooling garment with front view and back view. The prototype is composed of three parts: the layers forming the garment, the air treatment system, and the distribution channels. Reproduced with permission [249]. Copyright 2019, Springer Nature.

The gas expansion cooling garment is another type of personal cooling garment based on an integrated tube network distribution. Its operating principle is the endothermic vaporization of liquefied carbon dioxide (CO_2_), based on the distribution of CO_2_ at high pressure through a pressure relief valve in which the gas pressure drops to ambient pressure (Figure 5). During this thermodynamic evolution, the liquid CO_2_ is transformed into vapor and absorbs energy equal to the heat of vaporization of the gas and allows cooling of its immediate environment [229].

Despite its relatively lower total weight compared to water or air FCG garments and its high cooling capacity, the gas expansion cooling garment has a relatively short service life. In addition, the escape of CO_2_ from a closed environment can lead to hazardous gas concentrations if the device is used simultaneously by several workers in proximity [229]. In order to address some of the limitations of this type of cooling garment, a portable system using atmospheric discharge of CO_2_ at high pressure has been proposed to improve working conditions in hot and humid environments [229]. Thus, a prototype was developed. It consisted of a three-layer textile structure, an air treatment system using an atmospheric discharge of highly pressurized liquid CO_2_ to cool and dehumidify the airstream taken from the environment, two identical cylinders of saturated two-phase CO_2_ connected to a mixing chamber located inside a mixing box equipped with a heat sink, and distribution channels made of PVC tubing placed between the moisture-absorbing mesh layers of the garment, distributed at the back and front of the body. In this approach, the treated air was directed over the body to create a cool microclimate under the garment that cooled the body through convective heat transfer and assisted the evaporation of condensed sweat [229]. The evaluation of the performance of this prototype through tests carried out on male subjects in a hot and humid climate chamber demonstrated the capacity of such a concept to improve the thermal comfort of people by reducing thermal stress such as T_c_ and HR and the sensation of humidity. However, the conclusions of this study remain to be confirmed under real operating conditions and with other populations regarding the sex of participants, average age, and body weight. Some modifications should also be considered in the design of this prototype for use under personal protective equipment [229].

#### 4.3.4. Air Blast Cooling

Air blast cooling is another principle used. These types of clothing blow air onto the body and extract heat from it, improving the evaporation of sweat produced on the surface of the skin, while at the same time promoting heat exchange by convection using the speed of air passage over the body surface [58]. Most of these garments consist of two layers: an outer layer of waterproof fabric that prevents air leakage to the environment and an inner layer of air-permeable material that is directed between two layers toward the skin surface [250]. Since large air movements promote the evaporation of sweat, in some cases, the use of a compressor attached to the garment has been considered in order to project forced air. In addition, the use of a cooling device to cool the projected air could result in a greater temperature difference between the skin and its environment, thus promoting convective heat loss [227].

#### 4.3.5. Fan-Assisted Garment

This literature review has shown that from a portability perspective, most studies over the last ten years have focused on cooling by ventilation. These types of garments contain built-in fans to blow ambient air onto the skin surface to facilitate the evaporation of sweat. With the use of integrated mini fans a few centimeters in diameter, the cooling garment can remain light [251]. Although their cooling performance may be impacted by ambient air temperature or humidity, their great advantage is that they rely on the human body’s thermoregulatory mechanism to dissipate heat, thus eliminating the risk of overcooling [226,229].

In this context, tests conducted on male subjects in a climatic chamber have demonstrated the effectiveness of a ventilator-cooled garment in increasing heat loss while maintaining a constant T_s_ value during exercise in a hot and dry environment [252]. Calculation of the physiological strain index (PSI) with data collected during tests conducted in a climate chamber on male subjects wearing a cooling garment under a military suit showed that the projection of air onto the torso of individuals was more effective in a hot and dry environment compared to a hot and humid environment. However, the results showed an identical reduction in perspiration rates in both climatic conditions [253].

Some research groups have also made performance comparisons with passive cooling garments. Comparison of a jacket equipped with two ventilators on both sides of the abdomen and a vest with 21 pockets of PCM cooling under identical conditions showed no significant difference in the performance of the two garments in terms of torso T_s_ and HR of the female test subjects. However, the PCM garment provided a greater decrease in the microclimate temperature close to the skin and a better thermal sensation, while the fan-assisted garment further decreased the microclimate humidity [254]. The comparison of a cooling vest with frosted pockets and a fan-cool garment allowed the study of the subjective perceptions of workers in the horticultural and cleaning sectors when using such equipment during their workday. The data collected showed that male workers’ choice was more influenced by thermal comfort, while female workers paid more attention to tactile comfort and the feel of the fabric. This suggests that gender differences need to be considered in the design of this type of cooling clothing [255].

**Figure 6:**
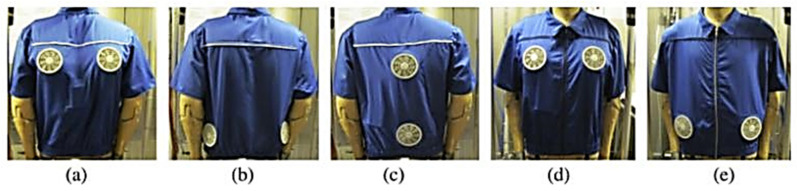
Small fans and openings on ventilated jacket located at different torso sites. The both fans are placed at (**a**) the upper back; (**b**) the lower back; (**c**) the mid back; (**d**) the chest (upper front); (**e**) the belly (lower front). Reproduced with permission [250]. copyright 2013 Elsevier.

Studies have also focused on optimizing the design of fan-assisted garments. The integration of two fans at five different locations in the upper back, lower back, middle back, upper front, and lower front of a cooling vest being examined on a breathable thermal manikin showed no significant difference in total torso cooling or total dynamic evaporation resistance of the garments (Figure 6). However, the local area corresponding to each ventilator was better cooled [250]. The effectiveness of a fan garment in providing greater comfort to workers working in offices with a warm environment was examined by wearing a short-sleeved shirt containing two ventilators on the abdomen associated with two side openings in the chest area and a third in the upper back. Tests conducted on female subjects with low physical activity in a warm laboratory environment showed that ventilation reduces T_s_ at the location of the ventilators, as well as the average T_s_ of the torso. However, a variation on the mean whole body T_s_ and T_rec_ was not observed [251].

Using numerical simulation of a series of two-dimensional models of convective and evaporative heat transfer to the skin surface, the efficiency of a fan-cooling garment was examined by considering different configurations in terms of the number and diameter of fans as well as different airflow speeds. Simulations showed that convective and evaporative heat transfer could be improved by the formation of vortex currents produced when the inlet air flows are high or when the space between the skin and the garment is wide enough [256]. Comparison of a continuous cooling mode with intermittent cooling on a 2-min operating and 2-min off cycles in a fan-cooled garment showed that constant ventilation could reduce heat stress to a greater extent during recovery phases. However, tests conducted on subjects wearing the garment cooling under a bullet-proof vest showed better perceptual benefits with intermittent ventilation during work and better perceptual benefits with constant ventilation at rest [257]. The use of ventilators has also been extended to the design of full-face respirators. A comparative examination of a conventional mask with a modified mask providing air under the mask near the forehead and a second modified mask providing air from the forehead to the eyes and into the breathing zone found that air projection through the integral ventilation reduced the T_s_ of the face and minimized the increase in T_c_ while improving the subjective assessment of comfort and thermal sensation in the test subjects [258].

#### 4.3.6. Thermoelectric Cooling

**Figure 7:**
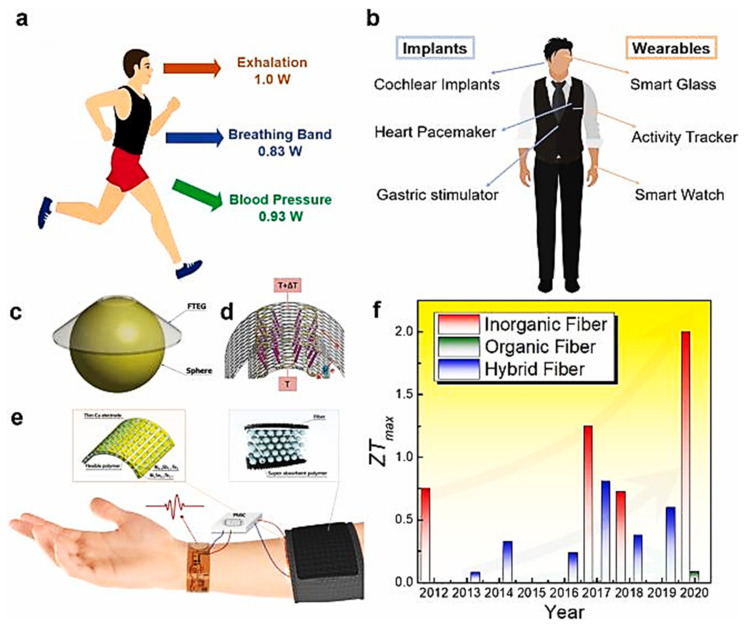
Illustrations of (**a**) the powers harvested by the human body [261]: (**b**) Several applications of wearable electronics. (**c**) A typical flexible thermoelectric generator (F-TEG) on a sphere. (**d**) The unit of the fiber-based F-TEG. Reproduced with permission [262]. Copyright 2017 WILEY. (**e**) A wearable thermoelectric power generator with a fiber-based flexible substrate. Reproduced with permission [263]. (**f**) The reported maximum ZT (ZTmax) for the fiber-based thermoelectric materials in recent years [264,265,266,267,268,269,270,271,272,273,274,275,276]. Reproduced with permission [277]. Copyright 2020, Elsevier.

Thermoelectric devices using thermoelectric cooling based on the Peltier effect [259] have also been used in the design of personal cooling garments [260], as shown in Figure 7.

A temperature-controlled glove was developed by combining thermoelectric modules with heat sinks in the form of mini-fans and a thermistor placed close to the skin. Using a feedback microcontroller of the integrated thermistor, the applied voltage could be used to cool or heat the modules. Despite the validation of the demonstrator developed on human subjects at the laboratory level, the optimization of the glove size and the area of thermoregulation remain to be investigated [278]. A cooling helmet based on thermoelectric refrigeration was proposed by implementing two air-cooled and water-cooled refrigeration modules that each included a thermoelectric element. Tests conducted on a thermal manikin revealed that the flow rate of the water circulation had a greater impact on the cooling capacity of the helmet and the coefficient of performance of the system [259]. A thermoregulatory garment was also proposed using the connection of a portable thermoelectric module to a network of air distribution tubes knitted into the garment. By changing the direction of the electric power supplied to the thermoelectric module, the modes of operation could be switched between cooling and heating. By examining the relationship between weights and thermal resistance of commercially available heat sinks, the study proposed a method to find the minimum weight of heat sinks for a portable thermoelectric system [279].

A flexible thermoelectric system has also been developed using elastomer layers, sandwiching rigid thermoelectric modules between two extensible sheets separated by an air gap to achieve low module thermal conductance and improved flexibility. Then, a demonstration vest was put in place covering the back, chest, and abdomen with more than 140 flexible thermoelectric modules [280]. Despite the small size of the thermoelectric modules allowing for portable solutions, it appears that these systems have relatively high electrical energy consumption and require the use of appropriately sized batteries [229].

#### 4.3.7. Active Evaporative Cooling Garments

**Figure 8:**
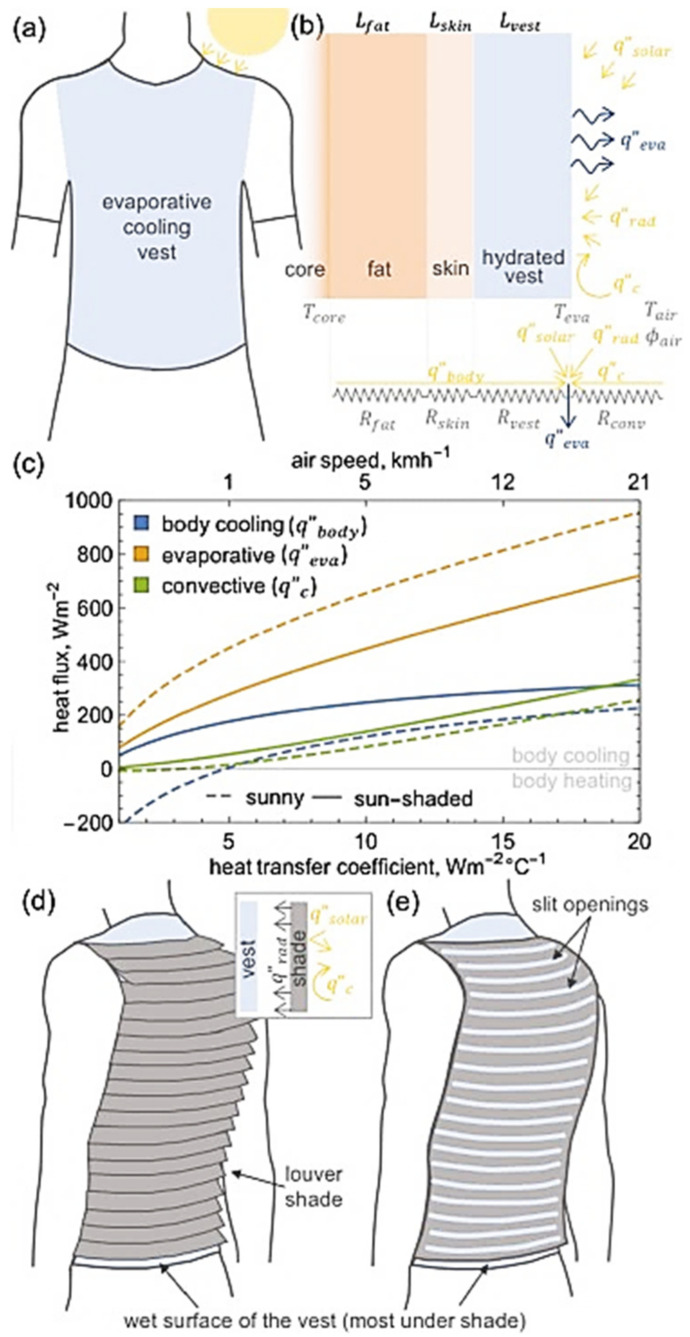
(**a**) Schematic of an evaporative cooling vest. (**b**) Corresponding cross-sectional schematic and thermal resistance network presenting different heat and mass transfer processes involved in evaporative cooling of the wearer. (**c**) A plot of body cooling, convective loss, and evaporative heat fluxes. (**d**,**e**) Schematic of evaporative vests with the (**d**) louver and (**e**) slitted shading structures. Reproduced with permission [282]. Copyright 2020 Elsevier.

The optimization of evaporation, being considered as the most efficient physiological means for heat dissipation, has also been the subject of studies on the development of cooling garments [226]. Conventional evaporative cooling garments take advantage of the high latent heat of water evaporation and provide a cooling effect by facilitating evaporation through a highly absorbent fabric structure [241,281,282,283], as shown in Figure 8. Then, the cooling effect lasts until all the moisture in the cooling garment evaporates. In this mechanism, the evaporation of water from a wet media or surface is typically used to cool the skin [53].

However, an evaporative cooling garment has the disadvantage of not being functional when worn under dense protective clothing. In addition, its effectiveness is greatly reduced with high ambient humidity [226]. One of the approaches proposed to improve the performance of evaporative cooling clothing in a humid environment has been the combination of a ventilation mechanism to wick moisture away more efficiently [284]. To circumvent some of the problems associated with evaporative cooling garments, portable and motorized evaporative cooling systems have also been explored. In this framework, a motorized vapor compression device assembled in a backpack configuration has been proposed to be combined with a cooling garment containing refrigerant lines [285,286]. Despite very satisfactory cooling rates using a motorized approach, the concept remains very cumbersome and impractical [286].

### 4.4. Comparison of Cooling Strategies

In view of the multitude of methods available for the design of personal cooling garments, some studies have focused on making comparisons between different techniques in order to propose the best cooling strategies for different conditions. Comparison of a garment containing two pockets of cooling PCM with a vest containing two fans on the front and back and a cold water FCG on human subjects under identical laboratory conditions found that for short cooling periods, active cooling techniques provided rapid initial reductions in T_c_, whereas a PCM-based device was more influential on T_c_ [287]. Evaluation of five cooling conditions for people wearing firefighter suits in a hot, humid environment showed that maximum T_c_ could be further reduced when a water-based FCG garment was combined with air ventilation from protective equipment ducts [288]. A study on a thermal manikin in combination with human testing, which compared the performance of a fan-assisted garment with two cooling PCM garments and a water-based FCG for military use, found that the fan-assisted garment also improved physiological responses in subjects to a lesser extent compared to other methods [289].

Cooling capacity, ability to keep the skin dry, operating time, and portability are characteristics that make it easier to choose the right cooling technique according to environmental conditions and activity. To this end, a comparative table has been proposed by experts [226]. Data collected from various studies in the literature show that FCG and vacuum desiccant garments provide the greatest cooling capacity. However, such comparisons are highly subjective, as depending on the climate, the number of cooling elements, and areas covered, some of the characteristics presented in Table 4 may vary.

From the perspective of the use of personal cooling garments in workplaces, universal methods have been proposed to facilitate the evaluation and selection of the most appropriate system according to the climate and the nature of the activity. Within this context, a cooling garment performance scale was proposed in order to present the potential success of an integrated system to provide thermal comfort under different environmental conditions. For this, a factor in the form of a dimensionless number between 0 and 1 was proposed, whereby the smallest value corresponds to the system’s lesser capacity to achieve thermal comfort [58]. In a related study, a method for calculating the effectiveness of a personal cooling garment in meeting the requirements of different types of work tasks has been suggested. This method considers the cooling capacity, weight, and operating time of the integrated cooling system, on the one hand, and the work rate, type of terrain, slopes, or work sites to be covered by the worker on the other hand [290]. However, in order to accurately predict the time required to complete a task, additional methods that include additional information on body heat loss with or without cooling clothing and the effect of cooling on the body and its physiology are needed [290].

### 4.5. Hybrid Cooling Garments

Due to the shortcomings of the cooling methods used in the design of personal cooling garments and the complexity of selecting the best strategy for different activities and environments, some experts have opted to implement hybrid cooling technologies [291]. Although they appear to be more efficient than those using a single technology, hybrid cooling garments can become more cumbersome than systems with a single technology [229]. The combination of frozen pads with integrated fans was one approach explored. In this context, a garment containing three frozen gel pockets and two fans mounted on the lower back was tested in a warm and humid climate chamber. The results of the tests carried out on male subjects confirmed the effectiveness of such a hybrid cooling garment in reducing physiological stress during exercise. However, the concept remains to be validated for other types of activities and with subjects of other fitness characteristics [292].

The effectiveness of garments equipped with frozen pads and integrated fans was also validated in a study of 130 Hong Kong workers in the construction, horticulture, and outdoor cleaning, catering, and airport parking sectors, who generally expressed higher levels of perceptual comfort when wearing the cooling garment [293]. A concept combining PCM pockets with cold water circulation was also studied.

For this purpose, PCM pockets integrated into a jacket to cool the torso were associated with a water pipe concealed through the PCM pads to circulate cold water from a microcooler to refreeze the PCM and extend its duration of action. Simulation work was used to optimize the parameters related to the type of PCM and the coolant circulation and to adjust the jacket’s tightness. Subsequently, tests conducted on human subjects with a prototype developed from the simulated optimizations showed that hybrid cooling would remain effective for at least two hours of work indoors without sacrificing thermal comfort [294].

Over the last five years, several studies have been dedicated to exploring hybrid cooling garments combining PCM cooling elements and integrated fans (PCM/fans) to ensure better performance in hot and humid climates [295].

To evaluate the performance of PCM/fan hybrid cooling garments, a prototype containing four fans and 24 pockets of PCM [296] and a garment with two fans and 24 pockets of PCM [297] were tested on thermal manikins. The presence of fans greatly improved evaporative heat loss compared to the situation where the fans were turned off. Although PCM actuators offer limited cooling time, a hybrid garment would provide a certain level of cooling throughout the test period due to the presence of fans in both hot/dry and hot/humid environments [296,297]. In addition, the study of a jacket with eight PCM pockets and two fans on the lower back by a sweaty thermal manikin in a hot and humid climate also showed that a higher cooling power would be achieved by hybrid cooling compared to PCM-only or fan-only cooling configurations [298]. A suit containing 24 PCM pockets and four fans distributed across the lower back of the jacket and the side pelvis of the pants was also tested on a thermal manikin in hot/dry and hot/humid climates. The results revealed that in dry conditions, the cooling speed in the initial phases was higher with the use of PCM without turning on the fans. On the contrary, in wet conditions, the cooling speed was lower without the fans. In addition, hybrid cooling provided a significant continuous cooling effect for the duration of the tests. According to activity simulation tests conducted on the thermal manikin, although the PCM alone or the fan alone can provide some degree of cooling for light work, it is indeed the hybrid cooling that leads to an optimized performance for heavy work conditions [299].

A study conducted on human subjects concluded that PCM/fan-cooling garments could effectively reduce heat stress during exercise in a warm, moderately humid environment. Indeed, the use of a suit containing 18 PCM pockets in the upper body and six thigh pockets in combination with two ventilators on the lower back of the jacket and two ventilators on the lateral pelvis of the pants reduced subjects’ T_c_, mean T_s_, HR, and PSI, while improving subjective perceptions during exercise and recovery phases [300].

A similar combination of 24 PCM pockets and four fans was also validated for a warm indoor environment simulated by a climatic chamber by demonstrating a reduction in the mean T_s_ and total sweat production of subjects, who also expressed good thermal sensations, skin moisture, and comfort compared to tests without cooling [295]. The effectiveness of a cooling jacket equipped with two fans on the lower back area and eight PCM pockets distributed on the front and back of the body was also evaluated through a series of 14 field studies conducted during the summer with 140 Hong Kong construction workers. Wearing the vest during break phases led to a significant reduction in thermal sensation, RPE (rating of perceived exertion) scale, HR, and PeSI (perceptual strain index) in the subjects compared to breaks without cooling. This PCM/fan vest also showed a good ability to attenuate workers’ perceptual heat strain index during breaks of limited duration but with much less effect for extended breaks. However, a thorough study of optimal work–rest duration with cooling by a hybrid cooling garment remains to be done [301].

#### 4.5.1. Design Optimization of Hybrid Cooling Garments

A few studies have also been devoted to optimizing the design of PCM/fan garments to ensure better management of cooling energy using an additional layer of insulation in the garment structure [302,303]. A vest with a structure of two layers of firm fabric and equipped with a pair of fans installed on the lower back and eight PCM pockets evenly distributed on the front and back of the body was tested during the rest phases of the male subjects in activities in a hot and humid climatic chamber. The results of these tests highlighted the ability of the hybrid jacket to decrease participants’ T_c_ and HR and improve their subjective perceptions [303]. For the optimization of a wetsuit with two fans in the lower back of the jacket, two fans in the side pelvis region of the pants, 18 PCM pockets placed on the front and back of the jacket, and six pockets in the thigh area of the pants, a polyethylene insulation sheet has been inserted between the PCM pockets and the outer layer of the garment. The results of tests performed on active human subjects in a hot and humid climatic chamber demonstrated that such a design could provide a relatively cool microclimate around the wearer’s body while minimizing the rise in the average T_s_. The study suggests the use of such a design for moderate physical activities in a hot environment thanks to an extended duration of the cooling of the PCM ensured with the presence of an additional insulation layer [302].

A similar study taking into consideration the same arrangement of 24 PCM pockets and four fans placed in the jacket and pants of a suit proposed the integration of an insulating layer composed of polyethylene foam on the outer surface of the PCM pockets to reduce the heat absorption of the hot environment and extend the operating life of the PCM used. Subsequently, Tanabe’s thermoregulation model coupled with a model of heat and moisture transfer through the garment was used to numerically study the performance of this new hybrid cooling garment. According to parametric digital analyses, the environmental heat absorbed by the PCM can decrease thanks to the increase in thermal resistance provided by an additional insulating layer. The validation of the model by tests carried out on males in a hot and humid climatic chamber also demonstrated that the presence of an insulating layer in the structure of a garment with PCM/fans could considerably reduce the environmental heat absorbed by the PCM. Thus, the total PCM melting time and the effective cooling time could increase [291].

#### 4.5.2. Numerical Analysis of Hybrid Cooling Garments

Some work has also been devoted to the numerical analysis of the performance of PCM/fan garments under different conditions [256,304,305]. In one of these studies, a mathematical model was proposed to calculate the transient transfer of heat and humidity through layers of clothing incorporating PCM pouches and fans. Once validated by experiments performed by a prototype placed on a hotplate, the model was integrated into a bioheating model in order to simulate an individual working in hot and dry conditions at different metabolic rates. Numerical simulation results showed that running the fans during the transient period of sweat absorption by layers of interior fabric could cause unwanted heating effects and increase the melt fraction of the integrated PCM. However, these unwanted effects were eliminated by running the fans after the end of this transitional period to achieve increased heat loss in the torso region, which therefore improved the comfort and feel levels at tested metabolic rates [306]. Another digital model has been proposed [305] to analyze heat and humidity transfer through a PCM/fan combination having 24 PCM pockets and four fans with the same arrangement described in the work of [295,300]. For this purpose, a clothing heat and moisture transfer model coupled with a multimode human thermoregulation model was developed to determine thermophysiological responses under dynamic environmental conditions. In addition, the parts covered and not covered by the PCM pockets were considered, and a method for calculating the apparent heat capacity was used to address the behavior of the PCM. The moisture barrier effect of the PCM pockets as well as evaporation and condensation on the surface of the PCM pockets were also considered in the model. Model validation of the data from [300] of PCM/fan combination showed that heat absorption from the external environment by the PCMs and condensation of moisture on the surfaces of the PCM pockets proved to be the two major problems in hybrid cooling garments. However, proper ventilation could play an important role in removing a large amount of moisture and latent heat from this clothing system [305]. The performance evaluation of a suit equipped with 24 PCM pockets and four ventilators distributed in the jacket and pants was also the subject of numerical analyses including the simulation of different types of warm environments. According to numerical analyses of T_c_ and T_s_ values, high ambient temperature and RH ≥ 70% would weaken the performance of such a suit. However, for better cooling efficiency in conditions of very high environmental temperatures or RH, the properties of the PCM used and their level of insulation should be optimized [304].

### 4.6. Advanced Material Based Passive Cooling Strategies

With the development of advanced materials and the progress made in the elaboration of conductive textiles, these types of concepts have also been exploited for the implementation of passive cooling strategies in textile structures [57,307,308]. Examples of recent work in this field are the creation of artificial leather with very high thermal conductivity by mixing silver-coated nylon yarns with polyester yarns in a laminated structure using a polyurethane and methyl cellulose resin [309], the design of thermally conductive fabric with hybrid conductive yarns made of polyester yarns combined with copper filaments in two different alignments [310], the development of thermoregulatory textiles based on thermally conductive composite fibers of highly aligned boron nitride/polyvinyl alcohol having been synthesized by 3D printing to take advantage of the in-plane thermal performance of boron nitride [311], and the numerical simulation using the finite element method of heat transfer concepts through an aligned carbon nanotube layer to be integrated between two layers of textiles to ensure partial heat redirection to a cold reservoir in the design of a firefighting garment [312].

**Figure 9:**
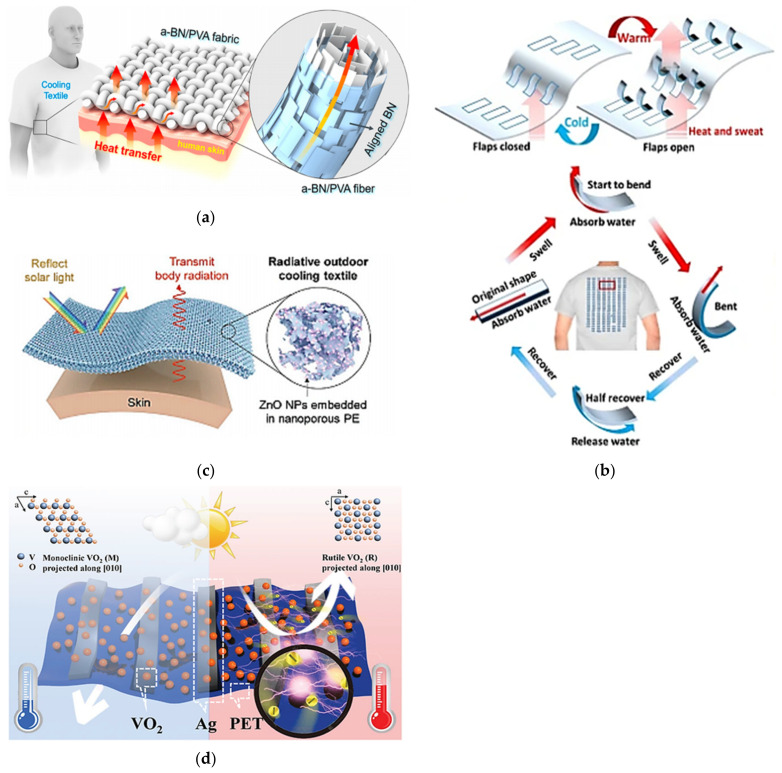
Wearable thermal textile: (**a**) Schematic illustration of the thermal regulation textile. The thermoregulation is established by conductive composite fibers. Adapted with permission [2]. Copyright 2017, American Chemical Society. (**b**) Mimic of thermo-adaptive functionality of human skin on one single Nafion flap. Reproduced with permission [4]. Copyright 2017. (**c**) Schematic of the ZnO nanoparticle-embedded textile. The spectrum was designed to be transparent to thermal radiation and reflective for sunlight for human body. Adapted with permission [5]. Copyright 2018 WILEY-VCH. (**d**) Thermal radiation management illustration of smart textiles with patterned silver strips on a PET substrate and combined VO_2_ nanoparticles. The thermal textile reversibly reflected heat at high temperature and was transparent to IR light at low temperature. Adapted with permission [6]. Copyright 2019 WILEY-VCH.

It has also been reported that mixing phase change materials with active cooling components such as metals and/or highly conductive ceramics and encapsulated soluble alcohols such as xylitol that cool in contact with water vapor could allow a PCM to repeatedly lose heat and thus create an effect similar to a recharging of the cooling effect of the PCM during exposure to heat. A study has shown that depositing a mixture of PCM/highly conductive metals on the surface of a sweater could allow the development of a textile layer creating a multistage cooling effect [313]. Nafion^®^, being a selectively and highly water-permeable, sulfonated tetra fluoro ethylene-based fluoro-polymer copolymer, has been the subject of recent work to develop a reversible moisture sensitive garment to support personal thermoregulation in warm environments. For this purpose, smart textile structures based on Nafion^®^, activated by moisture change, have been developed with the ability to rapidly and reversibly change their porosities or thermal insulation levels in response to the individual’s level of perspiration [314]. Indeed, a perspiration pore mimicking structure comprising a network of flaps on a sheet of Nafion^®^ could respond to a moisture gradient by automatically opening or closing to regulate the flow of air through the pores, thus providing humidity and temperature control. Nafion^®^ tapes inserted between two layers of variable thickness have also demonstrated the ability to adjust the air gap and change the thermal insulation between two layers of fabric [314]. Figure 9 shows some examples of smart textiles with thermal effects.

**Figure 10:**
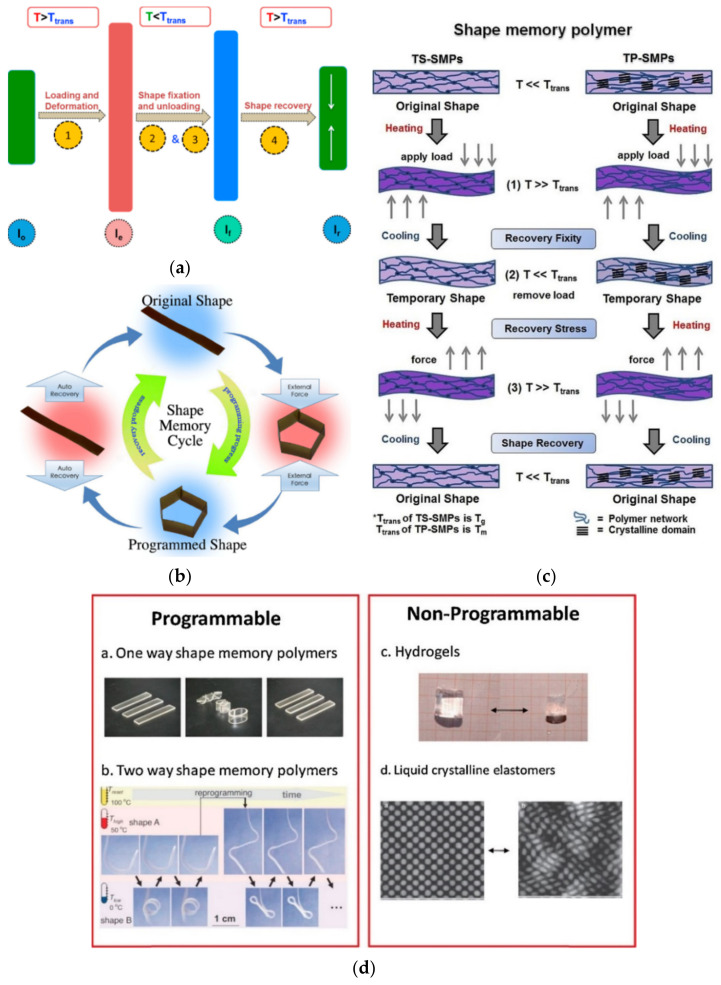
Shape memory polymers. (**a**) Schematic representation of sample deformation during shape memory testing cycle. Reproduced with permission [317]. Copyright 2015, Elsevier. (**b**) Shape memory cycle of two hot stages (red background) and two cold stages (blue background). The shape changes occur during the hot phase. Reproduced with permission [318]. Copyright 2018 Elsevier. (**c**) Main stages of thermally induced shape memory polymers [319]. (**d**) Classification of shape-changing polymers. Reproduced with permission [320]. Copyright 2015 Elsevier.

Shape memory polymers have also shown great promise in the development of thermoregulating textiles. These materials sensitive to external stimuli have the capacity to memorize a permanent macroscopic shape, be manipulated and fixed to a temporary shape under specific conditions of stress, and then later return to their original state by no longer being subjected to thermal, electrical, or environmental stress [102,315,316], as shown in Figure 10.

Concerning temperature-sensitive shape memory polymers, large changes in thermomechanical properties occur across the glass transition temperature of the melting point temperature of the crystals of their soft segment. In addition to these changes, it has also been shown that this type of material may exhibit changes in moisture permeability above and below this point [160]. For textile structures, this behavior can be very useful, as they can provide thermal insulation at cold temperatures and permeability at high ambient temperatures [102]. These materials are particularly interesting for creating cooling effects. Indeed, when a textile containing a shape memory polymer reaches the glass transition temperature, it transforms into a fabric permeable to water vapor and heat, allowing the release of body heat after intense activity or a rise in environmental temperature.

The material may return to a less permeable structure when the temperature drops [321]. Despite this potential, their cooling capacity is by no means comparable with that of the techniques presented in Table 4 [58]. The recent use of textiles containing shape memory polymers in commercial products [321] could suggest their association with techniques used in the design of personal cooling garments (Table 4), but the current literature review could not find such studies, or they must be rare.

Multilayer garments are another area for improving the performance of conventional evaporative cooling garments. This approach involves the integration of hygroscopic materials, of the desiccant or super-absorbent type to promote the absorption of the vapor produced by perspiration or by the liquid included in an internal reservoir [322]. Based on studies that have demonstrated the increased evaporation rate of water through the addition of desiccant materials, the desiccant cooling method has been combined with the vacuum cooling technique to achieve better performance. To promote the integration of desiccant elements into the garment structure, membrane technologies have also been proposed to separate the water contained in the cooling core from the desiccant material present in the absorption core. Polymeric membranes of the polyurethane or polyester type, being waterproof but permeable to water vapor, were chosen to allow water vapor to pass through while retaining the condensed water. Once a vacuum is created by a pump, the operation of these types of garments relies on the absorption of the vapor or adsorption by the desiccant in order to maintain the driving force for water evaporation [226]. Despite the high cooling capacity of vacuum desiccant cooling garments, very few studies have been devoted to them [229]. Examples of recent studies in this field are the integration of vacuum desiccant pads into a garment [125], the evaluation of an evaporative cooling garment to absorb heat and water vapor under an astronaut’s suit to be combined with a lithium chloride-based absorbent radiator to reject heat into space [323], and the development of a membrane desiccant fiber for vacuum desiccant cooling in view of the development of a vacuum desiccant garment [226]. It should be noted that the performance of desiccant systems based on evaporative cooling is much better in dry climates than in wet climates [57,322].

### 4.7. Commercial Cooling Garments

Commercially available cooling garments use different technologies. Table 5 shows the number of companies listed for each of the seven types of commercially available cooling technologies: active cooling systems such as circulating coolant (liquid and air) devices, gas expansion devices, air ventilation devices, thermoelectric devices, and passive cooling systems such as phase change materials (PCM) and evaporative cooling, as well as hybrid systems using two technologies.

Although the current study focuses primarily on active cooling systems, several commercially available products use PCM, a passive system, as the cooling technology (12 products). Most of the products are sleeveless vests, most often available in one size (or two sizes), with adjustment straps around the torso to ensure as close contact with the body as possible. The vests are equipped with PCM pockets on the front and back. Most companies do not provide details on the phase change materials used, claiming that their material is lighter and more effective than water or frozen gels and safer than ice water for the skin, which can cause frostbite. However, FlexiFreeze uses frozen water as a cooling principle but with specific packaging, claiming that for the same weight, water is a more efficient means of cooling than frozen gels. The company offers a vest with 96 ice cubes distributed on the front and back, weighing 1.4 kg. The FlexiFreeze product, similar to the AlphaCool Ice Vest, using a water-based product, has packaging that slows down the melting of the ice. Techniche uses pockets containing a non-toxic, non-flammable, and non-combustible carbon-based liquid. Overall, the majority of identified cooling vests appear to have similar properties: a temperature around 15 °C, a cooling period of approximately 2 to 3 h, a cooling capacity reactivation time of approximately 35-45 min, and a weight varying between 1.0 and 2.3 kg. There are a few exceptions. For example, AllTuff USA PCM vests are available in three charging temperatures, 5 °C, 15 °C, or 25 °C. The Ergodyne vest allows a reactivation time of 5 to 15 min. ClimaTech Safety’s CM2000 vest can provide an extension of the cooling period from 2 h (standard) to 4 h with the addition of another cooling layer attached by Velcro over the first layer.

Of the commercially available products using a passive water evaporative cooling system, three are presented herein. The first product is a thin, light, and flexible combination of a sleeveless shirt and shorts, which was developed by UNICO Swiss Tex GmbH. This close-fitting suit can be worn underneath clothing. It is made of a three-layer laminate consisting of two waterproof but breathable polyester membranes that cover a hydrophilic fabric [324]. The fabric acts as a container that can be filled with 30–60 mL of water using a syringe. This system lowers the skin temperature by 4 °C, and the cooling effect can last 40 min depending on the activity. The second product is the HyperKewl™ PLUS vest from Techniche. This vest is made of specialized fabric and fibers that allows for rapid absorption, stable water storage, and good evaporation. It is activated by soaking it in water and then removing the excess. This fabric is machine washable and can run for 150 wet/dry cycles. The third product is Ergodyne’s nylon-based Chill-Its 6687 vest that acts as a reservoir that can be filled with 400–450 mL of water. With patented technology, the vest gradually releases water by evaporation from the inside out, keeping the user cool and dry.

As a hybrid cooling system, one of the most interesting is that of the SurgeCool company, which has developed a vest using two technologies: a liquid circulation cooling system (active) combined with a frozen gel cooling system (passive). Instead of being equipped with a large ice tank (stationary or portable) and an injection pump, as most liquid circulation cooling systems are, SurgeCool replaced these elements with a gelling polymer pack. The liquid circulating through the vest tubes is cooled to a temperature of approximately 18–22 °C by the cooling pack, which will gradually melt and lose its cooling effect after 2 h. This assembly of the two technologies allows a more global cooling effect of the body, spreading the cold from the cooling pack over the whole vest in a certain way and for a longer period. The vest, with a single cooling pack, can be worn with the refrigerant pack on the front or on the back depending on the user’s work preferences. The vest weighs less than 1 kg.

UNICO Swiss Tex GmbH has developed a ballistic cooling vest together with the Empa Research Institute (Switzerland) that also uses two technologies: a passive evaporation system and an active system with fans. A panel, Coolpad, is filled with water, which evaporates through a membrane, cooling the panel. According to the company, the existing Coolpads were unsatisfactory: being subjected to high mechanical stress in the vest, they often leaked. Fortunately, a new laser diode welding technique has made it possible to produce thin, flexible, and reliable panels that do not leak, despite the mechanical stresses to which they are subjected. Two fans blow air through a spacer knit behind the Coolpad and provide additional cooling. The compression-stable, flexible spacer knit with low resistance to airflow was developed in cooperation with the Eschler company. A water refill is required for approximately three hours of use. The two fans, which can be recharged from a socket or a car cigarette lighter, can last three to four hours. This ballistic vest has been tested with the police officers of the Zurich City Police, who appreciated it. Finally, Techniche also offers a hybrid cooling product, simply combining two passive systems in the same vest: their PCM technology, CoolPaxTM, with their water evaporation technology, HyperKewlTM PLUS.

### 4.8. Cooling Actuator Challenges

Cooling actuators are the most studied technological solutions in an occupational health and safety context among the various means of intelligent thermal management (Table 6).

An important part of the research work on personal cooling garments has been devoted to fluid circulation cooling systems. Although their effectiveness has been approved by several studies, these garments are heavy and cumbersome and seem practical only for occupations in which workers do not travel frequently, such as workers working in vehicles with the refrigeration unit or compressed air system at a standstill. In addition, cooling units consisting of an ice cube tank, proposed for better portability of the system, remain limited due to the operating time and require frequent recharging of the tank. Furthermore, recent studies on the optimization of parameters such as the textile layer design of the fluid circulation cooling garments, their tubular network, their assembly, the capacity of their fluid injection pump, circulation flow rate, etc., seem to be limited to validation tests in the laboratory and on very few human subjects.

The automatic control of the appearance of steam around the tubular network to reduce the risk of skin burns in humid environments and the use of PCM suspensions to improve heat dissipation without an apparent increase in pump power are examples of other concepts proposed for optimizing the performance of cooling garments through fluid circulation, which generally operates with cold water. However, tests in operational environments are still needed to validate these concepts. Although many studies are devoted to fluid cooling garments, this analysis identified only two studies that were conducted in an operational environment.

Fluid circulation cooling garments with integrated temperature and flow rate control systems using intermittent or alternate circulation and T_s_ feedback activation would reduce system energy consumption and the risk of overcooling while improving efficiency. However, all work on these systems has been limited to tests on thermal manikins and few tests on individuals in the laboratory.

In order to take advantage of the relatively low weight and high cooling capacity of gas expansion cooling garments, while circumventing their limitations in terms of low operating time and exhaust gas, very recent studies have proposed some optimizations for use in hot and humid environments. These results have yet to be confirmed under real operating conditions. In addition, design modifications are still necessary to facilitate their use under protective equipment. The literature review also revealed that despite the greater efficiency of air blast cooling garments using an air compressor, most of the work had favored cooling by fan ventilation for better portability of the system. Although fans allow a good decrease in the humidity of the microclimate close to the skin, their performance seems to be influenced by the temperature or humidity of the ambient air and, according to some studies, their beneficial effect lies at the level of the local Ts without a remarkable influence at the level of the total T_s_ or T_c_. Despite optimization work on fan placement, additional openings in the clothing, or intermittent cooling modes, no particular benefit could be observed in the studies analyzed. On the other hand, the few studies devoted to the thermoelectric cooling garment seem to be limited to proofs of concept and show a relatively high electrical energy consumption in their current state. However, the small dimensions of thermoelectric modules, combined with recent work on flexible module design, suggest that portable thermoelectric solutions with heating and cooling capacity can be implemented. In order to overcome the low efficiency of evaporative cooling garments worn under dense protective equipment and in humid environments, the introduction of a ventilation mechanism to evacuate moisture, or motorized vapor compression devices associated with cooling lines, have been proposed. In their current state, these concepts are cumbersome and impractical to carry to work.

Vacuum desiccant cooling garments have a high cooling capacity, as they involve hygroscopic materials to improve the performance of evaporative cooling garments [341]. However, very few studies have been devoted to them. Research continues to propose new structures or new types of desiccant materials [226]. As they are passive systems, their operating time remains limited, and their performance is reduced in humid climates.

Despite comparative studies and the establishment of generic tables comparing the performance of various cooling systems, the selection of the most appropriate system is sometimes a complex task. One of the reasons for this difficulty is the shortcomings of the test methods used to evaluate the various types of personal cooling garments. Many studies have used thermal manikins. Although useful for a first draft, manikins cannot adequately simulate the spatial and transient thermal behavior of humans or realistic thermophysiological responses, such as changes in T_c_ and T_s_. They are also limited by the lack of a vasoconstrictor response initiated in human skin when cooled [58].

In addition, testing on human subjects is mostly limited to a restricted number and gender. In addition, differences in methodologies (i.e., exercise duration and intensity), subject characteristics (i.e., gender, fitness level, acclimatization, and hydration level), and cooling system properties (i.e., cooling duration, number of cooling elements, and their location) sometimes lead to confusion about the results presented in the literature. Indeed, the diversity of experiments and methods has sometimes led to different results that are not necessarily confirmed and are sometimes contradictory. For example, the impact of technology on physiological parameters may vary from one study to another. In addition, the application of laboratory results in the field may be compromised by ergonomic problems in a real work environment due to the varied and complex forms of movement in comparison with the simulated treadmill running-type tests adopted in many studies. In order to facilitate the choice of the appropriate system, some universal methods have been proposed for theoretical calculation of the performance of the cooling garment according to the climate and the nature of the activity. However, these methods do not consider the loss of body heat and the impact of cooling on the physiological aspects of the body, which can vary from person to person.

Faced with the complexity of selecting the best strategy for different environments and activities, and to circumvent the limitations of different systems, some experts have proposed hybrid cooling technologies. The combination of frozen gel pads with fans for a greater reduction of physiological constraints in the activity phases, and the circulation of cold water through PCM pockets to increase the operating time, have been the concepts explored through a limited number of studies. However, the review of the literature revealed several studies concerning hybrid cooling garments combining PCM actuators and fans (PCM/fans) in order to promote heat loss by evaporation using fans and to create a synergistic effect to obtain better performance in hot and humid climates while ensuring portability. The integration of insulating layers to reduce the environmental heat absorbed by the PCM in optimized versions of PCM/fan garments has increased the total melting time of the PCM and the effective cooling time. Despite great potential, moisture condensation on the surfaces of the PCM pockets, reducing the efficiency of the system, and the weight of the PCM pockets have proven to be two limitations of this type of hybrid cooling garment. Furthermore, a hybrid PCM/fan system has not yet been explored in a protective equipment structure.

In addition, the design of high thermal conductivity layers based on advanced materials or conductive textiles to improve the heat exchange of the garment, the mixing of PCM with conductive materials to create an effect of repeated heat loss, the use of materials reversibly sensitive to humidity or temperature have not yet been explored in association with active cooling systems in the design of hybrid cooling garments. These types of materials should also be studied in the optimization of the thermal performance of personal protective equipment. In view of the progress made and the use of new technologies in the design of personal cooling garments, the decision on the effectiveness or ineffectiveness of these new systems in reducing the body’s thermal stress requires more studies, modeling or simulations, in order to judge their performance under particular conditions.

Concerning research on products containing cooling actuators, analyses have shown that almost half of the products identified use passive cooling principles such as PCM or evaporative cooling. As in the scientific literature, most cooling garments based on liquid circulation or compressed air are stationary and less intended for workers who must move frequently. Despite the relatively simple design of fan cooling garments, no protective equipment containing integrated fans could be identified in the analyses. Gas expansion and thermoelectric systems appear to be still under development and not widely available on the market. Despite a great deal of research work dedicated to hybrid systems, there are few products combining two types of technologies. Moreover, none of these products combines an active fan system with a passive PCM system, even though they have been praised in the scientific literature.

## 5. Conclusions

Despite the standards governing working conditions and the advances in the development of more efficient protective equipment, thermal constraints remain a major occupational health problem. In such a context, thermoregulation systems, which make textiles capable of detecting, reacting, and adapting to thermal stimuli, offer great potential for improving the performance of personal protective equipment during exposure to extreme temperatures.

Therefore, the present study was conducted in order to better document the current state of knowledge on the technologies facilitating intelligent thermal management by reviewing the existing technologies currently available on the market and the developments carried out in the framework of previous research work. Particular attention was paid to the collection of scientific and technical information on systems that can potentially be integrated into personal protective equipment for intelligent and sustained thermal management throughout the execution of tasks. Based on the knowledge gathered and discussions on the current gaps in studies and marketed products, the efforts still to be done and the development or adaptation strategies to be deployed in personal protective equipment were discussed.

Indeed, the potential of smart textiles and advanced functional materials can be greatly exploited in the development of integrated temperature sensors, heating or cooling actuators, and wearable devices or in the optimization of their performance. The use of advanced functional materials in combination with active cooling or heating technologies to establish hybrid systems providing improved performance are among the most viable solutions to implement in the short term. Moreover, combining two cooling or heating technologies to create a synergistic effect to optimize the performances is one of the most interesting strategies to consider. Finally, the results of numerous laboratory studies and some products recently developed in the industry remain to be deployed in workplaces through field studies.

## Figures and Tables

**Table 1:** Temperature sensors to be integrated into textile apparels.

Technology Used	Integration Method	Operating Temperature Range	Reference
Temperature-sensing yarns incorporated in a knitted fabric	An off-the-shelf thermistor encapsulated into a polymer resin Multi-Cure^®^ 9-20801 (Dymax Inc.) micro-pod embedded within the fibers of a polyester yarn	Physiologically relevant temperature range of 25–38 °C	[78]
Electronic temperature sensing yarn	Knitted polyester-based armband demonstrator using a polyester yarn with embedded thermistor encapsulated into a polymer resin Multi-Cure^®^ 9-20801 (Dymax Inc.) and connected to an Arduino Pro Min Hardware	Tested to measure the temperature of a hot object of 65 °C	[83]
Yarn with embedded thermistor	NTC thermistor soldered to copper interconnects and encapsulated with a cylindrical micro-pod made of conductive resin (Multi-Cure^®^ 9-20801 by Dymax Inc.), then embedded in a polyester yarn	Tested in a range of 0 to 40 °C	[82]
Yarn with embedded thermistor	A commercial temperature-sensing element within a polymeric resin micro-pod embedded in the fibers of a polyester yarn	Tested in a range of heating-cooling cycle of 25–38 °C	[81]
Yarn with embedded thermistor	Commercially available NTC thermistor encapsulated in a polymer micro-pod made of UV curable resin (Multi-Cure^®^ 9001-E-V-3.5 by Dymax Inc.) embedded into the fibers of a thermoplastic monofilament yarn spun from liquid crystal polymer (Vectran^TM^)	NTC sensitive to 25–38 °C	[80]
Thermistor integrated into textiles	Embedded NTC thermistor and conductive textile yarns (Shieldex^®^ silver plated polyamide) in a belt made of soft bamboo yarns	25 to 43 °C	[79]
Embroidered hybrid resistive thread (RTD)	(1) Hybrid thread composed of three strands. Each strand contains 33 polyester fibers; only one includes one resistive stainless steel microwire, (2) The surface of the hybrid thread is covered by a silicone lubricant, (3) The sensor is embroidered in a helical meander-shaped structure into the carrier fabric made of KERMEL^®^, Lenzing^TM^ FR, Technora, and antistatic fibers	Temperature calibration (40 to 120 °C); rapid temperature cycling (−40 to 125 °C)	[92]
Embroidered resistance temperature detector (RTD)	Conductive silver R.STAT^®^ yarn as humidity and chromium–nickel austenitic stainless steel yarn as thermal sensors embroidered on a cotton substrate	Validated for 20 °C to 100 °C and 50 to 98% of RH	[90]
Temperature-sensing knitted resistance temperature detector (RTD)	Metal wire inlaid in the middle of a rib knitted structure of polyester fabric	Validated at 20–50 °C	[87]
Dip dyed yarn by PEDOT-PSS as RTD	RTD yarns fabricated by: (1) Dip dyeing cotton yarns in PEDOT-PSS solution, (2) Applying a silver paste applied at the two ends of the dyed threads to form electrical pads, (3) Creating encapsulation layer by dip dyeing the yarns in polystyrene to better protect against dust and moisture	Validated for −50 to 80 °C	[89]
Metal wires incorporated in a knitted fabric (RTD)	Knitted temperature-sensing fabric developed with two different wire inlay densities and a fine metallic filament embedded within the courses of a double-layer knitted structure made of poly acrylic/wool yarns	Validated at 20–60 °C	[88]
Flexible platinum-based resistance temperature detector (RTD) integrated into textile	Sensors manufactured by electron beam evaporation followed by photolithography on Kapton^®^ polyimide foils, then cutting the foil into stripes each containing an individual sensor and connecting lines, which are then inserted into a fabric during the weaving process	Validated for 25 to 90 °C	[142]
Optical fiber Bragg grating (FBG) based sensor integrated into textile	Encapsulating the optical fiber with polymeric (copolymerization of unsaturated methyl ethyl ketone peroxide (MEKP) and cobalt naphthenate) filled strips, then embedding it into the fabric by combining large and small pipes together in fabrication	Validated for body temperature ranging from 33 to 42 °C	[95]
Optical fiber Bragg grating (FBG)-based sensor integrated into textile	A textile structure of hollow double-wall fabric was adopted as a base, and quasi-distributed FBG sensors were embedded by the methods of cross-walls and between-walls for smart fabric sensor development	Validated in a T_env_ range of 20 to 130 °C with 10 °C steps and then decrease back to 20 °C with the same procedure	[96]
Textile thermocouple	Four different textile thermocouples: (1) Flat textile composed of pairs of textile electrodes: graphite non-woven—woven fabric with nirtil static fibers, (2) Linear textiles composed of pairs of textile electrodes: thread of Nitinol—static fibers—thread of steel fibers, (3) Flat linear thermocouple manufactured from pairs of electrodes: graphite nonwoven—silver-covered polyamide yarn, (4) Hybrid thermocouple composed of pairs of electrodes: steel knitted fabric—constantan wire	Validated for temperatures up to 70 °C and 90 °C	[86]
Thermocouple	(1) T_s_ measured by a thermocouple placed at the armpit with an elastic belt made of spandex, (2) T_env_ and the heat flux through the garment measured by modified platinum sensor array integrated into the outer garment of firefighters, (3) Sensors associated to a planar textile-based antenna made of conductive yarns	Heat flux sensor is able to operate in the range of −70 to +500 °C	[62]
Textile heat flow sensor	Insertion of a constantan wire within three different textile structures (polyamide-based knit, aramid non-woven, woven aramid-based), followed by a local treatment with polymeric resin to allow the partial copper deposition, then an electrochemical deposition of copper on the constantan wire to obtain a thermo-electrical wire and finally a post-treatment for polymer removal	Tested in a range of 30 and 80 °C and 0 to 150% moisture content	[97]
Sensorized glove/upper-arm strap	(1) A glove with two textile electrodes integrated inside in the proximal phalanx of the index and middle fingers on the inside of the glove and a temperature sensor placed in the tip of the ring finger of the glove, (2) Upper arm strap confectioned with two integrated textile electrodes and a temperature sensor placed in the inner lining of the strap	Validated for T_s_ measurements averaging 34 °C	[137]
Platinum sensor integrated into a jacket	Modified platinum sensor array (welded on Kapton^®^ polyimide foil) integrated into the outer firefighting garment (composed of external impermeable, thermal insulation Gore-Tex^®^ PTFE membrane, and internal comfort layers) to measure T_env_ and the heat flux through the jacket	Able to operate in the range of −70 to +500 °C	[134]
Working jacket with integrated sensors	Sensors and wireless communication integrated into a commercialized Wenaas^®^ working jacket, while packing sensors on the textile by vacuum molding using biocompatible silicon, and wiring external sensors to the main sensor module by conductive yarns also coated with silicon after vacuum molding	Verified in a climatic chamber −20 to 25 °C with RH 0% to 50%	[136]
Working jacket with integrated sensors	Infrared temperature sensor and two combined humidity–temperature sensors integrated into the jacket in three different areas, using two different packages: (1) sensor enclosed into a pouch made from Gore-Tex Paclite^®^ PTFE membrane, and (2) only the opening of the sensor covered with membrane made form Gore-Tex Paclite^®^	Validated at 22 °C and −5 °C	[135]
Firefighting clothing with integrated sensors	A firefighting garment with three main integrated components: physiological sensors (including the body temperature), fire-related sensors (including field temperature), and the computing node	N/A	[63]
Sailing garment with integrated sensors	The electronic system is consisted of a master system and a slave system placed inside a waterproof pocket above the cuff of a waterproof sailing top garment made of coated and laminated woven fabrics	N/A	[143]
Thermosensing armband, glove, and sock based on yarn with embedded thermistor	Temperature-sensing garments (armband and glove made of polyamide/spandex, sock made of cotton) containing thermistor soldered to copper interconnects and encapsulated with a cylindrical micro-pod made of conductive resin (Multi-Cure^®^ 9-20801 by Dymax Inc.)	Tested at 23 °C and validated for T_s_ ranging from 28 to 33 °C	[138]
Printed polymeric PTC and NTC thermistors	Carbon-based paste screen printed on Kapton^®^ polyimide foil	Validated at a range of 30 to 42 °C	[43]
Printed polymeric PTC and NTC thermistors	Resistive inks screen printed on polyethylene naphthalate and protected by a dielectric ink (CYTOP-like fluro-polymer) as a passivation layer, followed by a plasma post-treatment	Validated at a range of 20 to 90 °C	[116]
Printed polymeric NiO based NTC thermistor	Stable NiO ink (suspended in ethylene glycol aqueous solution) inkjet-printed in between two silver conductive electrodes on a polyimide substrate, then thermally cured at 200 °C for an hour	Validated at a range of 25 to 250 °C	[117]
Printed resistance temperature detector (RTD)	Silver complex ink inkjet printed on Kapton^®^ polyimide foil	Validated at a range of 20 to 60 °C	[119]
Printed smart bandage	Temperature sensor fabricated by PEDOT-PSS/CNT paste screen-printed on a nm-thick-SiO 2-coated Kapton^®^ polyimide, then cured at 100 °C for 10 min	Validated for 22 to 48 °C (normal T_s_ ≈ 29 to 31 °C)	[121]
Printed wearable resistance temperature detector (RTD)	Shadow mask printing of PEDOT-PSS/CNT suspension on SiO_2_-coated Kapton^®^ polyimide substrate and silver electrodes by screen printing	Validated at a range of 22 to 50 °C	[120]
Printed paper-based thermal sensor	(1) Ionic liquid, 1-ethyl-3-methyl imidazolium bis (trifluoromethylsulfonyl) imide ([EMIm][Tf2N]), inkjet printed on a regular paper, (2) Two gold electrodes deposited on the paper substrate through magnetic sputtering evaporation setup	Thermal responses validated at 25 and 45 °C	[123]
Printed resistance temperature detector (RTD) on paper	Silver nanoparticle ink inkjet printed on specific coated paper substrate	Validated at a range of −20 to 60 °C	[103]
Stretchable graphene-based resistance temperature detector (RTD)	(1) Silver nanowire first filtered as electrodes using polycarbonate filter membranes, (2) Graphene/nanocellulose dispersion then filtered as the detection channel to connect electrodes, (3) PDMS base and curer poured on top of the filtered films, then degassed and cured, (4) Solidified PDMS with embedded silver electrodes and graphene detection channels peeled off from the polycarbonate membrane to obtain a stretchable device	Validated at a range of 30–100 °C	[111]
Graphene-based wearable resistance temperature detector (RTD)	Graphene nanowalls deposited on a polydimethylsiloxane substrate with plasma-enhanced chemical vapor deposition technique and polymer-assisted transfer method, associated to silver paste electrodes	Validated at 35 to 45 °C	[114]
Flexible graphene-based resistance temperature detector (RTD)	Graphene oxide-based formulation printed on Kapton^®^ polyimide and polyethylene terephthalate substrates reduced by infrared heat lamp and then annealed at 200 °C	Validated in a range of 30 to 180 °C	[113]
Flexible composite-based resistance temperature detector (RTD)	Ni microparticle-filled binary polymer of polyethylene and polyethylene oxide composites with copper tape strips-based RFID antenna	Validated at a range of 35 to 42 °C	[85]
Flexible composite-based resistance temperature detector (RTD)	HCl doped poly-o-methyl aniline/Mn_3_O_4_ nanocomposite spin coated on glass substrate	RT characteristics in the temperature range of 35–185 °C with repeatability in the range of 75–185 °C	[124]
Flexible composite-based resistance temperature detector (RTD)	Dispersions of multiwall CNT drop casted onto gold electrodes fabricated on a polyimide substrate	Validated in a range of 20 to 60 °C	[127]
Flexible composite-based resistance temperature detector (RTD)	Graphite/PDMS composite dispensed on flexible polyimide films, associated to copper electrodes	Validated at 30 to 110 °C	[126]
Flexible CNT-based composite	Multiwall CNT/polyvinyl benzyl chloride derivative with trimethylamine (PVBC_Et3N) dispersions drop casted onto a gold electrode pair supported on a polyimide film	Validated for 20–40 °C	[128]
Flexible composite-based thermoelectric nanogenerator	A composite of the tellurium nanowires/poly (3-hexylthiophene) (P3HT) dropped onto a Kapton^®^ polyimide flexible substrate associated to two silver electrodes	A heat source of 24.8 °C	[125]
E-patch	A modular patch with electronics elements: (1) The thermometer prototyped by attaching a flexible adhesive-backed copper foil on a polyethylene terephthalate substrate, (2) The loop enclosed between two layers of a medical-grade adhesive dressings to attach the tag over the skin	Validated for T_s_ ranging from 32.7 to 34.7 °C	[132]
E-skin sensor	Two main technologies compared: (1) Arrays of 16 temperature sensors relying on thin serpentine traces of gold, fabricated using microlithographic techniques with thin layers of polyimide, (2) Multiplexed arrays of 64 sensors based on PIN diodes formed by patterned doping of nanoscale membranes of silicon	T ranging from 27 to 31 °C and 30.7 to 32 °C (during mental and physical stimulus tests)	[129]
Dual-heat-flux associated with two double-sensors	Two double-sensors with dual-heat-flux embedded in the neck pillow, while using rubber sheets to simulate the subcutaneous tissue layer of the neck during experiments	Tested at 32–38 °C	[144]
Heater-less deep body temperature probe	Dual-heat-flux method wired sensors placed on the skin, each probe containing the two insulators on a rubber sheet	Validated at 36.5–37.5 °C	[145]
Double-sensor thermometer	The sensor consists of two temperature probes on each side of a standardized insulator placed in a plastic shell	Validated at 36–37.8 °C	[146]
Double-sensor thermometer	Combined heat and skin sensors specially sealed in a polycaprolactone-based enclosing cover	Validated at 10, 25 and 40 °C	[147]
Double-sensor thermometer	Combined skin and heat flux sensors specially sealed in a polycaprolactone-based enclosing cover	Validated for a body temperature of 36–38 °C	[148]
Wearable thermistor	T_s_ measured by a textile strip wristband containing a NTC thermistor	16–42 °C	[149]
Wearable thermometer	Array of 4 × 4 Silicon Kelvin precise sensor thermometers integrated into a textile-based affixation aid to the arm, associated with a signal processing chain	25–41 °C	[150]
Wireless connected temperature sensor	T_s_ of the hand measured by a connected temperature sensor	0–100 °C	[151]
Wireless connected temperature sensor	The system consists of a transceiver, a microcontroller, and a digital temperature sensor enclosed in a polycarbonate covering to be placed under the subject’s arm	Validated for T_s_ (36.7 to 37.3 °C) in an ambient environment	[152]
Long-range RFID tag	RFID rigid tag based on temperature dependence of the frequency of the ring oscillator integrated in a ceramic package and assembled to a matched impedance dipole antenna designed on high-dielectric constant ceramic substrates	35 to 45 °C	[130]
Epidermal RFID-UHF tag	Tag and antenna layout with adhesive copper transferred on a polycaprolactone membrane attached on a skin with a hypoallergenic cosmetic glue	Validated at 30 to 42.5 °C	[131]
Remote HR and body temperature monitoring	A temperature sensor integrated into a polyurethane flexible substrate wearied on the left thumb, while being connected to a programmed microcontroller	Validated for body temperature range of 36.6 to 37.2 °C	[153]
Remote HR and body temperature monitoring	A portable temperature sensor connected to an analogue microcontroller measuring the body temperature, with the final product being packaged in a small lightweight polymeric package	Validated for body temperature range of 36.6 to 39.4 °C	[154]
Wireless humidity and temperature sensor	A semiconductor temperature and RH sensor affixed to the internal surface of an N95 filtering face-piece respirator made of highly hydrophobic nature of polypropylene	Validated for 30–36 °C and 60–89% RH	[155]
Wearable in-ear thermometer	(1) Thermal sensors integrated into a textile based earbag in order to measure the tympanic temperature inside the ear, T_s_, and T_env_, (2) The earbag added to a resizable headset shielding the outer ear	Validated for the body temperature range of 34.5 and 37 °C	[156]
Graphene-coated lens of IR thermopile sensors for an ear-based device	(1) Graphene/isopropyl solution drop casted over the silicon substrate of the lens of commercial IR thermopile being associated to a microcontroller collecting the temperature measured, (2) The ear hook-type enclosure 3D printed using Accura Xtreme polymeric resin, while covering the thermopile with a silicone cushion	Validated at T env of 21 °C and a body temperature range of 36.5 to 37.5 °C	[133]

**Table 2:** Number of companies listed for electric heating actuators for use in intelligent thermal management.

Type of Product Technology	Warm Clothing (Jacket, Vest, Shirts, Pants, Gloves, Scarf, Beanie, Socks)			Heated Insoles (and Socks)	E-Textile	Mask	Total
	Fabricant ^1^	Brand Sold on Online Platform ^2^	Smart Apparel ^3^				
Conductive heating elements	8	3	3	2	3		19
Electric heating wires	8	1		1	3		13
Heating based on carbon fibers	20	9					29
Graphene technology	2	1					3
Technology PTC			1		2		3
Inspired air heating						1	1
Total	38	14	4	3	8	1	68
	56						

^1^ Manufacturing company (with a website); ^2^ Brand sold exclusively on online platforms (ex.: Amazon) and whose manufacturer does not have a website; ^3^ Intelligent heating clothes offering self-regulation of the temperature.

**Table 3:** Heated actuator.

Technology Used	Integration Method	Operating Temperature Range	References
Silver ink-based printed heater	Heat-curable ink (Fabinks-C4001 silver ink) direct dispenser printed on UV-curable ink (Electra EFV4/4965 dielectric) as printing interface and untreated woven polyester/cotton fabric	Heating up to 33 °C	[200]
Ag nanowire-coated heating fabric	Heating fabric made of pre-cleaned bare cotton fabric dipped in ethanolic solutions of silver nanowires for 5 min, then dried at 80 °C for 10 min	Heated up to 50 °C under an applied power density (30–150 °C can be obtained according to the applied voltage)	[201]
Silver filament-based heating membrane	Flexible and waterproof heating nano-silicon carbide (SiC)/thermoplastic polyurethane (TPU) hybrid membranes (prepared by pouring modified nano-SiC/TPU solution into a mold with silver filaments)	Depending on the applied voltage (1.4–5.14 V), a maximum temperature of 20–160 °C	[202]
PEDOT coated-based heating fabric	In situ polymerization of poly (3,4-ethylene dioxythiophene) p-toluenesulfonic acid (PEDOT-PTSA) on a textile polyester fleece	With a surface resistance down to 10 Ω/sq can even reach 170 °C by applying 24 V	[203]
PEDOT coated-based heating fabric	Vapor phase polymerization of PEDOT coatings on the textiles (pineapple and cotton fiber-based fabrics)	Cotton-coated fabric generated 28 °C when connected to a 4.5 V battery and 45 °C when connected to a 6 V battery	[204]
Poly pyrrole-coated textiles	Polyamide knitted fabric impregnated soaked with pyrrole and then dipped into polymerization solution of the dopant (p-toluenesulfonic acid) and the oxidizing agent (Iron (III) chloride hexahydrate)	45 to 105 °C produced depending on the heated surface area	[191]
Carbon fiber-based composite as a heating element	Polyacrylonitrile-based (T-800s) recycled carbon fiber sheet with polyurethane binders (three types were used: Primal ECO-16, Resin HF-05A, and Emuldur DS 2361 PU)	Heating up to 96 °C (20 to 96 °C range)	[208]
Carbon fiber-based electroconductive heating textile	Carbon-based electro-conductive textile (from Gorix Inc.) integrated in a carbon fiber composite laminate and woven glass fiber plies	Tested at 0 °C, −10 °C, and −20 °C in an environmental chamber	[209]
Carbon fiber-based heating elements	A commercialized carbon fiber-based resistive-heating blankets (SmartCare by Geratherm Medical AG) compared with air or water-warming systems	Providing 42 °C during 120–150 min	[210]
Carbon fiber-based heating elements	A heating garment based on a carbon fiber fabric with carbon content that can be divided into surface and linear heating	N/A	[213]
Vest based on a Carbon polymer heating element	Electrically heated vest (six strips of carbon polymer heating elements made from the ultrathin, biothermal carbon fiber inserted into six front and back sacks inside a polyester woven vest) worn with cotton knit underwear and a military uniform (polyamide/cotton) in different sequences	Heating up to 24 to 26.5 °C depending on the placement of the elements	[212]
Vest based on a carbon polymer-based heating element	Electrically heated vest (carbon polymer fabric-heating element in a polyester vest with polyamide batting) of four-layer structure with protection layer, heat-insulating layer, heat-generating layer, and base layer	Providing 34 °C around torso skin and 38 °C on the outside surface of the electrically heated vest, tested at 0 °C and −10 °C; 30% RH; 0.4 m/s of air velocity	[211]
Electrically heated garment based on carbon heating wire-based garment versus chemically heating garment	Two heating technologies compared: (1) Two types of heated ensembles by embedding seven heating elements into the vest (each heating pad was manufactured by sandwiching carbon heating wire between two layers of high-density woven polyester fabrics), (2) polyester-based ensembles with 14 chemical body warmers	Validated at 2.0 ± 0.5 °C and 85 ± 5%; 44 °C by the electrically heated garment and 46 °C by the PCM garment	[162]
Stitched heating actuator	A single-trace serpentine pattern of silver-coated Liberator40^®^ conductive fiber (by Sysco Advanced Materials, Inc.) that has a polyester Vectran^TM^ core (Kuraray Co. Ltd.) stitched on an elastomeric knit fabric	Heating up to 33–40 °C	[177]
Stitched heating actuator	Electrical heating system using Liberator 40^®^ conductive fiber with a polyester Vectran^TM^ core stitched on stretch knit fabrics (cotton/spandex, polyester/spandex, nylon/spandex)	20–140 °C heat generated depending on the number of thread layers, the thread spacing, and the knit fabric type and fabric covering	[54]
Sewn silver-based yarn	Heating element based on conductive yarns made from stainless steel fibers or polymer yarns that have been coated with silver or copper	A maximum temperature of 37–39 °C	[178]
Stitched silver-coated heating actuator	Heating actuator made of stitching silver-coated polyamide yarn over polyester plain woven fabric	Heat generated in a range of 27 to 43 °C	[166]
Silver-coated yarn vs. Stainless steel	Two types of stainless steel and two types of silver-coated polyamide with different linear density and yarn structures	A maximum temperature of 38–55 °C depending on the knit structure	[187]
Silver-coated yarn-based woven fabric	A simulation model derived to compute the resistance of conductive woven fabric, validated with two silver-coated conductive polyamide 6 and polyamide 6.6-based yarns blended with cotton in three woven structures	N/A	[199]
Silver-coated polymeric yarn-based heating element	Thermo-mechanical properties of knitted structures mathematically modeled and validated on an elastomeric and silver yarn knitted structure	27.4 °C, 30.1 °C, and 31.6 °C depending on the plain, rib, and interlock structures while applying 3 V	[190]
Silver plating yarn-based heating knit	Silver plating compound yarns fabricated by twisting three silver filaments and polyester staple fiber spun yarns utilized in three types of knit (plain stitch, ribbed stitch, and interlock knit)	25–70 °C can be produced depending on the applied voltage and the knit structure	[183]
Ag nanowire-coated heating fabric	Two conductive yarns (silver-coated yarn with polyamide 6 and polyamide 6.6 inner fibers) embedded into normal knitted woolen fabrics	25–55 °C produced depending on the applied voltage	[182]
Woven silver filaments or coated silver yarns-based heating element	Relation of function of parameters of the heating fabric expressed by an equation for a design prediction, validated on woven fabrics made of cotton/Tencel™ lyocell blend using different conductive components such as silver filament, silver-coated yarn, and coated silver knitted fabric	Three different fabrics with set up resistance of 10 Ω, 14 Ω, and 18 Ω, providing different levels of temperature	[195]
Steel-based fiber panels	Panels construction made of continuous stainless steel filament yarns based on metal fibers with polyester yarns	30–50 °C depending on the amount of the ply of the pad	[186]
Fine copper wire and fusible interlining fabrics	Non-woven and woven interlining as substrates, bonded fabrics of nylon and cotton, copper wires all bonded by thermal fusing	21–95 °C produced depending on the applied voltage	[188]
Heat-insulated shape-memory element-based EHG	The fabric made of three layers of non-wovens from the blends of flax and steel fibers and the two interlayers included spirals, made from Nitinol (NiTi) or copper (Cu) wire	34–40 °C produced depending on the applied voltage	[159]
Conductive-coated yarn-based knitted or sewn fabrics	Knitted structures by using different conductive yarns made of stainless-steel fibers covered by polyester fibers (DA5393, DA5340; Bekitex 50/1)	35–60 °C produced depending on the design and the fiber type	[185]
Weft knitted heating pads	Acrylic, polyester as main yarns and three different conductive yarns (Copernic non-insulated (9 Ω), Thermaram hybrid (5.8 Ω), Thermotech-N non-insulated (9.6 Ω))	Copernic (35.2–48.8 °C)/Thermaram (33.4–60.28 °C)/Thermotech-N (35.4–48.4 °C) depending on the main yarn composition	[184]
Conductive knitted fabric based on elastic-conductive composite yarn	A spandex filament as the core and a stainless-steel filament combined with rayon fibers as a helically wound sheath around the spandex core, embroidered on fabric knit with spandex content	Tested at 20 °C, 65% RH and heat generated in a range of 30 to 90 °C depending on the applied voltage	[189]
Conductive knitted fabric based on stainless steel yarn	A physical model in order to predict the electrothermal behavior of stainless-steel knitted structure, validated by a stainless-steel heating fabric associated to bus-bars of highly conductive silver-coated polymeric yarn	Produced heat depends on the knit structure: 1.5 V applied: 35.6 °C (plain) 42 °C (interlock); 3 V applied °C (plain) 84 °C (interlock) 99 °C	[192]
Conductive knitted fabric based on silver-coated yarns	A theoretical model proposed to control the temperature of conductive knitted fabrics, validated by conductive knits made of two types of conductive yarns (a monofilament of 68.6 Ω/cm and a silver-coated yarn of 1 Ω/cm embedded into different knitted wool fabrics)	25 to 60 °C depending on the applied voltage and the loop arrangement	[193]
Conductive knitted fabric based on silver-coated yarns	An electrothermal model considering thermal conductivity coefficient, specific heat capacitance, fabric mass, and initial temperature, validated by the average temperature of the knitted fabric of wool, cotton, and acrylic blended with silver-coated conductive yarns	45 to 70 °C, depending on the blend type and the loop density	[194]
Conductive knitted fabric based on silver-coated yarn	The resistance of conductive knitted fabrics modeled by contact resistance and the superposition of the length-related resistance and contact resistance, validated on two overlapped conduct yarns and conductive knitting stitches (composed of silver coating yarn and a cotton yarn) under unidirectional extension	Initial resistance of two overlapped yarns varying from 2 to 6 Ω	[196]
Conductive knitting stitches	Equivalent resistance of a knitted stitch with different courses and different wales modeled, validated on knitting materials that included one non-conductive yarn made of wool and three Statex-conductive silver-coated yarns, designed in two types of knitting stitches (jersey and intarsia)	The global resistance depends on the course/wale’s configuration	[197]
Conductive knitted fabric based on silver yarn	A sheet resistance method to compute the resistance of conductive fabrics from a macroscopic view, validated on a knitted fabric (wool associated with two conductive silver-coated yarns resistance of 1 Ω/cm and 4.7 Ω/cm)	An equivalent lump resistor of the conductive fabric paths is modeled	[198]
Electrical heated sleeping bags	Heating sleeping bag was developed by incorporating heating fabrics into the feet region of the bag (no precision on the heating element composition)	Tested at 5.5 °C and −0.5 °C, 80% RH; 0.4 m/s wind speed, with a heating capability from 22 to 34 °C	[214]
Electrical heated glove	Heating plates fixed in the back side of the limiting layer of the fingers in glove (no precision on the heating element composition)	(a) Tested in an environmental temperature of −130 °C; (b) the gloves are supplied active heating to keep the finger temperature higher than 15.6 °C	[215]
Electrical heated garment	A jacket with integrated heated elements (Powerlet rapidFIRe Proform Heated Jacket Liner by Warren)	Produced heat of 50 °C tested on subjects after swimming in the pool water temperature of 27.6 °C (Air temperature 23.4 °C, 56% RH)	[216]
Controlling the heating temperature of the vest based on a steel-based fiber panel	Heating vest composed of a heating system based on pads using stainless steel yarns with single-, double-, three-, and four-ply configuration. The heated panels were mounted onto the carrier using Velcro tapes worn under a garment made of cotton as outer layer, polyester as the lining, and polyamide as the net-like fabric	Depends on the amount of the ply pads and the power source	[217]
Temperature-regulated clothing	A newly developed metal composite embroidery yarn made of polyurethane-coated copper filaments for both temperature sensing and heating textile	Operating temperature set to 20 to 40 °C	[56]
The self-regulating garment	Heating garment composed of: (1) The actuator based on silver-coated polyester Vectran™ multifilament yarn stitched in a serpentine pattern, (2) The garment designed in a three-layer assembly: the heating element on the outside of the polyester/spandex knit base layer; an aluminized biaxially-oriented polyethylene terephthalate film layer above to improve heat retention; and a textile cover layer on the outside, (3) The self-regulated garment device with integrated closed-loop T_s_ feedback using NTC thermistors placed immediately underneath each zone and a microcontroller-based control system; (4) The user-controllable self-regulated garment with the thermistor feedback	Generated heats from 20 to 80 °C depending on the applied power	[164]
PCM associated with heating textile	Clothing system consisting of four layers: (1) Cotton fabric, (2) Non-woven polyester fabric treated with/without PCM enclosed in small polymer micrometric spheres with or without conductive heating fabric, (3) Non-woven polyester fabric, (4) Waterproof breathable fabric as the outermost layer	25–33 °C depending on the structure	[218]
CNT-coated triacetate cellulose-based fibers	Metatextile with dynamically adaptive infrared optical properties to directly regulate thermal radiation. Each fiber is elliptically shaped, with triacetate and cellulose components fused side by side, knitted, and then coated by few-walled CNTs in a process similar to solution dyeing	N/A	[221]
Water-perfused trousers	Water-perfused trousers with an adjusted water temperature of 43 °C	Tested in an ambient environmental temperature	[222]

**Table 4:** Characteristics comparisons of various types of cooling apparel [58,226].

Personal Cooling Garment	Cooling Capacity (Watt)	Average Weight (Kg)	Average Operating Time
By liquid circulation	50–600	3–5	3 to 6 h
By air circulation	270–320	4–5	2 to 6 h
By ventilation	75–350	0.5–1	2 to 8 h
By evaporation	50–70	1–3	1 to 2 h
By vacuum desiccator	320–370	3–4	2 to 3 h
For PCM materials	50–140	4–5	20 to 40 min

**Table 5:** Number of companies listed regarding cooling actuators used in thermal management.

Type of Product Technology	Vest ^1^	Jacket	Leggings (Chaps)	Other Clothing ^2^	Ballistic Vest	Gloves	Helmet	Total
By liquid circulation	3			2				5
By air circulation	1		1			1		3
By air ventilation	2	1		1			1	5
By gas expansion	1							1
Thermoelectric				1				1
By PCM ^3^	11		1					12
By evaporation	2			1				3
Hybrid system	2				1			3
Total	22	1	2	5	1	1	1	33

^1^ Some products (3/20) are sold exclusively by distributors. ^2^ Clothing can be vests, shirts, short or long pants, leggings, short or long-sleeved jackets. ^3^ Phase change material.

**Table 6:** Cooling actuator.

Technology Used	Integration Method	Operating Temperature Range	References
High thermal conductive artificial leather	Silver-plated polyamide yarn blended with polyester yarn (base layer)/dry or wet laminated resin (PU resin, solvent, methyl cellulose)	N/A	[309]
CNT-based fabric	The concept of heat transfer through a layer of aligned CNT stacked between two textile layers (insulation material)	Simulation conditions: (1) Hot environment (40 °C) and light work (332 W); (2) Hot environment/strenuous work (889 W); (3) Firefighting environment (58 °C) and light work; (4) Firefighting environment and strenuous work	[312]
Thermally conductive copper filament	Hybrid conductive yarns made of polyester yarn pooled with copper filaments of different diameters using cover yarn technique	N/A	[310]
Thermally conductive composite fibers	Thermally conductive and highly aligned boron nitride/polyvinyl alcohol composite fibers synthesized by 3D printing	Simulation conditions: T_s_ (37 °C); T_env_ (25 °C)	[311]
Nafion-based interlayer for adaptive insulation	Nafion^®^ N117 polymer from Dupont (polymeric chains including both hydrophobic polytetrafluorethylene backbone and hydrophilic perfluoroether sulfonic acid side chains) dried and annealed at 130 °C before using	Tested at 32 °C, 90% RH	[314]
Blend of PCM/highly conductive metals	UnderArmour^®^ polyester/spandex shirt with PCM/ACC (ACC, i.e., active cooling component blend: highly conductive metals and or/ceramics, encapsulated dissolvable alcohols such as xylitol) micro-printed inside the shirt	Tested in a climate chamber: 35 ± 1 °C; 55 ± 6% RH	[313]
PCM	Cooling vest made of polyester and separate pockets containing 21 PCM packs	Tested at T = 55 °C, RH = 30%	[325]
Peltier effect created by conductive fabrics	Direct current applied across two dissimilar polypyrrole-coated fabrics	Temp drops from 40 to 22 °C during 30 min while thermoelectricity decreases from 0.16 to 0.1mV	[326]
Temperature-controlled glove	A modified polyester glove with integrated thermistor placed closed to the skin and thermoelectric coolers attached to the textile with thermally conductive epoxy	Tested at 21 °C, 9 °C, −9 °C	[278]
Flexible thermoelectric device (cooling and heating)	Double elastomer layer design, sandwiching thermoelectric pillars between two stretchable sheets separated by an air gap	(1) From heating temperature change of 10 °C to the cooling temperature change of −8 °C depending on the applied current; (2) T_s_ kept at 32 °C in a T_env_ varying from 22 to 36 °C	[280]
Potable thermoelectric device (cooling and heating)	The thermoelectric unit conversion unit supplying cool or warm air through a tree-like rubber tube network knitted into an undergarment	T_s_ of the manikin fixed at 34 °C, tests performed at 21 °C	[279]
Thermoelectric cooling helmet	Helmet based on both air-cooled and liquid-cooled thermoelectric refrigeration using polyvinyl tubing network	Tested at 30, 32, 34, 36, 38, and 40 °C, while maintaining the average temperature of the thermal manikin at 32 to 34 °C	[259]
Air-cooling garment (ventilation)	A ventilated vest blowing ambient air using flexible vented polymeric ducts woven into the vest across the front and back of the garment	Tested in hot (45 °C), dry (10% RH), ambient	[252]
Air-cooling garment (ventilation)	Air-cooling garment composed of textile materials and flexible polymeric tubing, and environmental air ventilation along the torso	Tested in 40 °C–30% RH; 30 °C–70% RH	[253]
Air-cooling full-face piece respirator (ventilation)	Silicone-based modified full face piece respirator supplying air into the mask using an axial fan, flexible PVC tubing, and customized ports	Tested at 32 °C dry bulb (TAIR) and 50–60% RH	[258]
Air-cooling garment (ventilation)	Short sleeve jacket made of cotton/polyester with two integrated ventilation units	Approved at T = 34 °C, RH = 60%, air velocity = 0.4 m/s.	[250]
Air-cooling garment (ventilation)	A short-sleeve shirt with two integrated ventilation units	Climate chamber (38 °C, 45% RH, 3 kPa water vapor pressure, 0.4 m/s air velocity)	[251]
Air-cooling garment (ventilation) versus frozen pads	Two cooling vests are compared: vest A (flame-resistant fabric containing four pieces of frozen gel pads) and vest B (inflaming retarding fabric with two small fans and three pieces of frozen gel pads)	N/A	[255]
Air-cooling garment (ventilation)	A polyester-based jacket with two integrated small fans compared with a polyester-based vest incorporated with 21 PCM packs	Tested at 32 °C, RH = 50%	[254]
Forced-air ventilation	A forced air ventilation built into a textile body armor	Tested at 40 °C, 20% RH	[257]
Numerical modeling of ACG (ventilation)	Series of micro-fans, placed in a textile ribbon and attached to a woven textile garment	Simulation performed at 27–30 °C. 40% RH	[256]
Vacuum desiccant cooling garment	Garment with 12 vacuum desiccant cooling pads based on a semi-permeable and a microporous hydrophobic PTFE membrane, polypropylene honeycomb spacer, and multilayered polyamide/polyethylene bag	Validated at 40 °C and 50% relative humidity	[322]
Wearable engine-driven evaporative cooling system	The cooling system consists of an engine-driven vapor-compression system coupled with a cooling garment including refrigerant lines	Tested at 37.7–47.5 °C	[285]
Wearable engine-driven evaporative cooling system	Engine-driven vapor compression system assembled with a cooling garment consisted of the insulation, the heat transfer surface, and the refrigerant tube layer	Performs over a range of ambient temperatures (37.7–47.5 °C), evaporator refrigerant temperatures (22.2–26.1 °C), and engine speeds (10,500–13,300 RPM)	[286]
Evaporative cooling garment	The cooling generated by evaporation of water from a porous, hydrophilic pad sandwiched between a Nafion pocket and a hydrophobic expanded PTFE laminate	Tested on a simulated skin at a temperature of 33.2 °C	[323]
Evaporative cooling vest	A quilted polyamide outer layer, a water-repellant polyamide liner, and an elastic trim of cotton/polyester	Tested at 36 °C/33% RH, 36 °C/67% RH, 40 °C/27% RH, 40 °C/54% RH	[284]
Liquid cooling clothing	A vest with a network of fine PVC tubes sandwiched between two-layer polyester mesh, a backpack storing a pump, batteries, and an ice pack cooling reservoir	Tested at 36 °C/33% RH, 36 °C/67% RH, 40 °C/27% RH, 40 °C/54% RH	
Liquid cooling garment	Two cooling garments compared: (1) A light-weight vest (polyester mesh inside and PU laminated polyester fabric in pocket area) filled with superabsorbent acrylic resin pads, (2) a PVC tubed vest connected to a cold liquid reservoir placed in a sealed bag	Tested at 30 °C, 50% RH	[225]
Liquid cooling garment	Two types of polyethylene spandex-based garments with different PVC tubing length for the cooling liquid circulation	Tested at 35 °C and 50% RH	[327]
Liquid cooling garment	Two types of polyethylene spandex-based cooling garments with different PVC tubing length	Tested at 35 °C and 50% RH	[328]
Liquid cooling garment	A long-sleeved T-shirt (Coolmax^®^ polyester knitted fabric) and a vest (Coolmax^®^ polyester knitted fabric) constituting the insulation layer of the coolant PVC-based tubing system	Tested in climatic chamber 26 °C–30% RH and 35 °C–30% RH	[329]
Liquid cooling garment	Long-sleeve underwear made of a specially developed two-layer knitted fabric (polyester/elastomer as the inner layer and cotton/elastomer as the outer layer) with a spacer module for PVC-based tubing	Climatic chamber at 30 °C, of 40% RH, and 0.4 m/s of air velocity	[330]
Liquid cooling garment	Tube-lined (PVC-based) perfusion vest (polyester based) using field-portable cooler	Tested at 33 °C, 60% RH	[331]
Liquid cooling garment (water-perfused suit)	A commercially available water-perfusion vest made of polyester and laminated around silicone tubing connected to a backpack made of silicon, polyamide, and polyester	Tested at 33 °C, 60% RH	[231]
Liquid cooling garment	Three cooling vests compared: an ice-based cooling vest, PCM cooling vest, and water-perfused suit	Tested at 35.2 °C; 49.2% RH; <1 m/s	[224]
Liquid cooling garment (water-perfused suit)	Water-perfused suit compared to PCM and ice vest	Tested at 35 °C and 50% RH	[224]
Liquid cooling garment	(1) Cotton shell liquid cooling vest with flexible tubing routed throughout vest, (2) cotton vest shell with four PCM packs, (3) polyester vest with 22 PCM packs, (4) cotton shell vest with five gel ice packs	Tested at 32 °C and 92% RH	[332]
Numerical simulation of a liquid cooling garment	Numerical simulation using a finite element method. The model validated thermal manikin, chiller, and liquid cooling	Simulated at body temperature of 40 °C and an external temperature of 23 °C	[333]
Fittable liquid cooling clothing	A cooling garment composed of polyvinyl tubing attached with silicone rubber tubing on the trunk area and adjustable with Velcro straps	Tested at 35.89 ± 1.25 °C, 35% RH	[334]
Liquid cooling garment	A vest covering the chest and composed of heat exchanger polyvinyl silicon tube line, an ice-water backpack reservoir, and a small battery-operated motor pump	39.4 °C dry bulb temperature; 41.2% RH; 32.7 °C wet bulb globe temperature	[335]
Liquid cooling garment	Two different liquid cooling garments (outer layer single jersey wool knitted fabric with plain weave and fusible interlining versus 10 × 3 rib wool knitted fabric; with any interlining) but the same tubing lengths and the inner layers	Tested on manikin temperature of 40 ± 1 °C and a test cabin temperature of 23 ± 1 °C	[232]
Liquid cooling garment	A knitted fabric used for the front and back of the cooling (two-layer piece for the sides of the garment made of polyester Coolmax^®^/spandex, spacer piece for tubing made of polyester Coolmax^®^/Spandex, pieces for the top and bottom of the garment made of cotton/spandex, channel for tube implementation made of polyester Coolmax^®^/spandex	Tested at 20 °C and 65% RH	[233]
Liquid cooling hood	Flexible PVC tubing distributed based on the thermal sensitivity of different body areas in a garment made of cotton or polyester/spandex	Tested at 24 °C with RH of 24 + 2%	[234]
Liquid cooling garment for NDX-1 space suit	Polyester spandex-based garment with a tubing network of flexible PVC tubes	Tested when T_s_ between 30 and 37 °C	[235]
Heat transfer model of liquid cooling garment	A spandex/cotton garment including flexible PVC cooling tubing system, the check valve, the switch, the micro-pump, the portable power supply, the ice pack, and the liquid reservoir	Tested on manikin surface temperature of 35 °C	[236]
Liquid cooling garment with PCM suspensions	Microencapsulated PCM (particles wrapped by a thin polymer shell, Microtek ^®^) suspensions used as the cooling fluid compared to a water liquid cooling garment made of cotton	Tested at an inlet temperature of the cooling garment of 11, 13, 15 °C; and the T_c_ of the thermal manikin 37 °C	[246]
A thermoregulatory model implanted for the liquid cooling garment	Fiala’s thermoregulatory model implemented in a liquid cooling garment environment	Validated at a 700 W metabolic rate	[336]
Liquid cooling garment	Spandex clothing without any cooling device compared with: (1) a liquid cooling and ventilation garment integrating a vinyl tube knitted to spandex underwear, (2) a liquid cooling made of elastic spandex with self-perspiration induced by water permeation from pores created on the vinyl-based tubes	Tested at 27 °C and 47% RH	[241]
Liquid cooling garment controlled by T_s_	Liquid cooling garment made of cotton or Nomex^®^ aramid fabric woven or laminated around small-diameter Tygon^®^ flexible polymeric tubing	Tested at 30 °C and 30% RH	[239]
Liquid cooling garment controlled by a T_s_ feedback	Modeling several studies using a water-perfused liquid cooling garment (T_s_ controlled, constant and pulse cooling methods)	Tested at 30 °C, 30% RH	[240]
Liquid cooling garment	Liquid cooling garment made of cotton or Nomex^®^ aramid fabric woven or laminated around small Tygon^®^ flexible polymeric tubing (intermittent and continuous cooling methods)	Tested at 30 °C and 30% RH	[237]
Liquid cooling garment controlled by different algorithms	A mobile liquid cooling garment made of spandex fabric and vinyl tubing tested at continuous, alternating, and pulsed cooling	T_s_ of the manikin is varying from 27 to 35 °C depending on the cooling control strategy	[238]
Liquid CO_2_-based liquid cooling garment	A cooling garment based on the endothermic vaporization of liquefied CO_2_ (Porticool, Inc) with vaporized cool and dry CO_2_ vented over a thin cotton layer	30 °C WBGT	[247]
Air-diffusing garment (tubing)	A dry air ventilation provided with an air-diffusing garment made of 3D space knitted fabric and stellate tubing worn between an underwear and impermeable chemical protective clothing	Tested at 25 °C, 50% RH, 0.2 m/s wind	[248]
CO_2_-based air cooling garment (gas expansion garment)	The air treatment system using an atmospheric discharge of highly pressurized liquid CO_2_ to cool and dehumidify the constant stream of air in a cooling garment made of polyester outer layer, moisture-wicking fabric middle layer, polyester mesh inner layer, and PVC tubes	Tested at 35.7 °C dry bulb and 86% RH	[337]
CO_2_-based air cooling garment (gas expansion garment)		Tested at T env = 22 °C and 40% RH and climate chamber with a dry-bulb temperature of 30 ± 1 °C and 60% RH	[249]
CO_2_-based air cooling garment (gas expansion garment)	Air-cooling systems analyzed by calculating the cooling capacity of the gaseous CO_2_-free jet expansion by three different approaches in a cooling garment made of polyester outer layer, moisture-wicking fabric middle layer, polyester mesh inner layer, and PVC tubes	CO_2_ used to cool a constant hot and humid airflow set at 37 ± 0.5 °C (dry bulb) and 69 ± 1% RH	[338]
Wearer-controlled vaporization garment	Two cooling systems compared: (1) a simulated liquid cooling and ventilation garment integrating a vinyl tube knitted to a spandex underwear, (2) a liquid cooling made of elastic spandex with self-perspiration induced by water permeation from pores created on the vinyl-based tubes	Tested at 27 °C and 47%, RH	[242]
Hybrid cooling garment (liquid cooling/air cooling)	Fiberglass-based helmet containing solution-associated air cooling and water cooling	The cooling capacity validated for the temperature changing in the helmet (25–40 °C) and (25–35 °C) for the temperature changing of LED driving modules	[339]
Hybrid cooling garment (liquid cooling/air cooling)	(1) Liquid cooling and ventilation garment made of vinyl tubing and spandex fabric, (2) liquid cooling garment made of elastic spandex and polyester	Validated in a typical laboratory environment	[340]
Hybrid cooling garment (PCM–liquid cooling)	Combining PCM with water pipes buried in the PCM in a cooling garment made of cotton lining, porous polyester support fabric, floss insulation vest, and PVC tubes	N/A	[294]
Hybrid cooling garment (gel pads–air cooling)	A hybrid cooling vest with light taffeta as the shell fabric integrating two fans and three gel packs	Tested at (1) 25 ± 1 °C/60 ± 3% RH (standardize the initial body condition); (2) outdoor WBGT (26.31 to 35.60 °C)	[293]
Hybrid cooling garment (frozen pack–air cooling)	A commercially available hybrid cooling vest (airproof outer fabric and meshed inner fabric) integrating three frozen gel packs made of water-based gel and fire-retardant fabric and two small detachable electronic fans	33 °C and 75% RH with partial water vapor pressure of 3750 Pa	[292]
Hybrid cooling garment (PCM-Air cooling)	24 PCM packs and four fans embedded in a cooling garment made of polyester	Approved at 34.0 °C, RH = 75%, and 28%	[297]
Hybrid cooling garment (PCM–air cooling)	Cooling uniform (cotton/polyester) containing two ventilation units and 24 PCM packs inserted into separate pockets and vertical ventilation pathways	Tested at air temperature of 22 °C, 50% RH; and evaporative resistance tests performed at 40% RH	[297]
Hybrid cooling garment (PCM–air cooling)	A jacket with polyamide taffeta based outer layer and mesh spacer inner fabric containing eight PCM packs and two fans inserted at the lower back of the vest	Tested at 34.0 °C, 60% RH, and V = 0.4 m/s	[298]
Hybrid cooling garment (PCM–air cooling)	Four ventilation fans and 24 PCM packs integrated into a cotton/polyester-based cooling uniform	Tested at (1) 30 °C, 47% RH (three different air velocities of 0.4 m/s; 0.15 m/s; 1 m/s)	[299]
Hybrid cooling garment (PCM–air cooling)	Long-sleeved jacket including a polyamide outer and a mesh liner layers with 24 PCM packs and four integrated ventilation fans	Tested at 36 ± 0.5 °C and RH = 59 ± 5%	[300]
Hybrid cooling garment (PCM–air cooling)	Long-sleeve cotton/polyester jacket and pants containing 24 PCM packs and two ventilation fans installed at the lateral pelvis area	Tested at T = 34.0 ± 0.5 °C, RH = 65 ± 5% and V = 0.15 ± 0.05 m/s	[295]
Hybrid cooling garment (PCM–air cooling)	Two fans and eight PCM packs inserted inside a jacket with a polyamide outer layer and mesh spacer inner fabric	Tested in environmental temperature ranging from 29.2 to 31.3 °C	[301]
Hybrid cooling garment (PCM–air cooling)	Vest with polyester inner and polyamide taffeta outer layers, containing two ventilation fans and eight PCM packs	Tested at 37 °C, 60% RH, and V = 0.3 m/s; 450 W/m^2^ solar radiation	[301]
Hybrid cooling garment (PCM–air cooling–insulation)	Cooling uniform with polyester mesh fabric lining and cotton outer layer containing four fans, 24 PCM packs, and one expanded polyethylene insulation layer between PCM packs and the outer clothing layer	Approved at 36 °C, RH = 59%	[303]
Hybrid cooling garment (PCM–air cooling)	Cooling uniform with polyester mesh inner fabric and cotton outer layer containing four fans, 24 PCM packs, and one insulation layer made up of expanded polyethylene foam placed onto the outer surface of PCM packs	Simulation and experimental validation: 36 °C and 59% RH (and air velocity in experiments of 0.10 ± 0.05 m/s)	[302]
Hybrid cooling garment (PCM–air cooling)	A mathematical model developed for transient heat and moisture transfer through clothing layers incorporating PCM packs and ventilation fans for a cooling garment made of polyester inner and cotton outer layers	The simulation cases of the planned parametric study: 25 °C and 50% RH (ambient); 40 °C and 35% RH (hot, dry)	[291]
Hybrid cooling garment (PCM–air cooling)	A numerical model developed to analyze heat and moisture transfer through the hybrid personal cooling garment made of polyester inner fabric and a cotton outer layer containing four fans and 24 PCM packs	Validated with data collected at T env = 36.0 ± 0.5 °C, 59% RH	[306]
Hybrid cooling garment (PCM–air cooling)	Cooling garment made of polyester inner and cotton outer layers containing 24 PCM packs and four ventilation fans	Conditions used in the numerical parametric study: (1) RH = 50% and T = 32, 34, 36, 38, and 40 °C; (2) T = 36 and 40 °C and 30, 50, 70, and 90% RH	[305]
Air cooling garment compared to a PCM garment and a liquid cooling garment	Three different cooling garments compared: PCM garment; air cooling garment; and liquid cooling garment	Tested at 31.20 (0.20) °C and 70 (1.90)% RH	[304]
Air cooling garment (ventilation) versus PCM versus liquid cooling garment	Four different commercial cooling garments compared: Ventilation Vest (Entrak), PCM Cool Under Vest (Steele), PCM PCVZ-KM Vest (Polar), and liquid cooling garment Hummingbird II (CTS)	Thermal manikin (35 °C, 40% RH); Human subjects (42 °C, 20% RH)	[287]
Air cooling garment (ventilation) versus PCM versus vapor compression	Four commercial cooling garments compared: Ventilation Vest (Entrak), PCM Cool Under Vest (Steele), PCM PCVZ-KM Vest (Polar), and a direct-expansion vapor-compression refrigeration garment Hummingbird II (CTS)	Air (dry bulb) temperature = 42.2 °C; 20% RH; Mean radiant temperature = 54.4 °C	[289]

## Abbreviations

CNT	Carbon nanotube
CPC	Chemical protective clothing
CPE	Chlorinated polyethylene
LED	Light-emitting diode
HR	Heart rate (number of heartbeats per unit of time)
HPPE	High-performance polyethylene
FCG	Fluid cooling garment (cooling clothing by circulation of fluid)
NTC	Negative temperature coefficient
PB	Poly benzimidazole
PEDOT-PSS	Sodium poly (3,4-ethylenedioxythiophene)-poly (styrene sulfonate) polymer complex
PCM	Phase change material
PDMS	Polydimethylsiloxane
PeSI	Perceptual strain index
PPE	Personal protective equipment
PSI	Physiological strain index
PTC	Positive temperature coefficient
PU	Polyurethane
PTFE	Polytetrafluoroethylene
PVC	Polyvinyl chloride
PVDC	Polyvinylidene chloride
RFID	Radio-frequency identification
RH	Relative humidity
RPE	Rating of perceived exertion (scale of perception of effort)
RTD	Resistance temperature detector (electric resistance temperature detector)
T_c_	Core (internal) body temperature
T_s_	Skin temperature
T_rec_	Rectal temperature
T_env_	Environmental temperature
UHF	Ultra-high frequency
